# Loss of Super-Enhancer-Regulated circRNA Nfix Induces Cardiac Regeneration After Myocardial Infarction in Adult Mice

**DOI:** 10.1161/CIRCULATIONAHA.118.038361

**Published:** 2019-04-05

**Authors:** Senlin Huang, Xinzhong Li, Hao Zheng, Xiaoyun Si, Bing Li, Guoquan Wei, Chuling Li, Yijin Chen, Yanmei Chen, Wangjun Liao, Yulin Liao, Jianping Bin

**Affiliations:** 1 Department of Cardiology, State Key Laboratory of Organ Failure Research, Nanfang Hospital, Southern Medical University, Guangzhou, China (S.H., X.L., H.Z., X.S., B.L., G.W., C.L., Yijin Chen, Yanmei Chen, Y.L., J.B.).; 2 Department of Oncology, Nanfang Hospital, Southern Medical University, Guangzhou, China (W.L.).; 3 Department of Cardiology, Guizhou Medical University, Affiliated Hospital, China (X.S.).; 4 Department of Cardiology, Guizhou Provincial People’s Hospital, Guiyang, China (B.L.).

**Keywords:** enhancer elements, myocardial infarction, regeneration, RNA, circular

## Abstract

**Background:**

circRNAs (circular RNAs) are emerging as powerful regulators of cardiac development and disease, but their roles in cardiac regeneration are still unknown. This study used superenhancers to distinguish key circRNAs in the regulation of cardiac regeneration and explored the mechanisms underlying circRNA functions.

**Methods:**

We used integrated bioinformatics analysis of RNA sequencing data and superenhancer catalogs to identify superenhancer-associated circRNAs. Quantitative polymerase chain reactions and in situ hybridization were performed to determine the circRNA expression patterns in hearts. Gain- and loss-of-function assays were conducted to detect the role of circRNAs in cardiomyocyte proliferation and cardiac repair after myocardial infarction. Chromatin immunoprecipitation (ChIP) and electrophoretic mobility shift assays were used to determine the binding of Meis1 (Meis homeobox 1) on circNfix-associated superenhancers. RNA pulldown and luciferase reporter assays were used to study circRNA interactions with proteins and miRNAs (micro RNAs).

**Results:**

We identified a circRNA, Nfix circRNA (circNfix), that was regulated by a superenhancer and overexpressed in the adult heart in humans, rats, and mice. The transcription factor Meis1 bound to the superenhancer at the circNfix locus, and increased its expression. In vitro and in vivo, cardiomyocyte proliferation was increased by knockdown of circNfix, whereas it was inhibited by circNfix overexpression. Moreover, circNfix downregulation promoted cardiomyocyte proliferation and angiogenesis and inhibited cardiomyocyte apoptosis after myocardial infarction, attenuating cardiac dysfunction and improving the prognosis. Mechanistically, circNfix reinforced the interaction of Ybx1 (Y-box binding protein 1) with Nedd4l (an E3 ubiquitin ligase), and induced Ybx1 degradation through ubiquitination, repressing cyclin A2 and cyclin B1 expression. In addition, circNfix acted as a sponge for miR-214 to promote Gsk3β (glycogen synthase kinase 3 β) expression and repress β-catenin activity.

**Conclusions:**

Loss of superenhancer-regulated circNfix promotes cardiac regenerative repair and functional recovery after myocardial infarction by suppressing Ybx1 ubiquitin-dependent degradation and increasing miR-214 activity and thus may be a promising strategy for improving the prognosis after MI.

## Abstract 2

Supplemental Digital Content is available in the text.

**Clinical Perspective**

**What Is New?**

- Silencing circRNA (circular RNA) Nfix promotes cardiomyocyte proliferation and angiogenesis by suppressing Ybx1 (Y-box binding protein 1) ubiquitin-dependent degradation and rescuing miR-214.
- Superenhancers mark abundant, tissue-specific, and developmental stage-specific circRNAs.
- circNfix-associated superenhancer regulates circNfix expression by recruiting transcriptional factor Meis1 (Meis homeobox 1).

**What Are the Clinical Implications?**

- The findings that circRNA Nfix negatively regulates cardiac regeneration provides a novel therapeutic target to improve the prognosis after myocardial infarction.
- Understanding the role of superenhancers on controlling mouse cardiac regeneration may uncover the epigenetic modifications restricting the regenerative ability of adult human heart, enabling the development of potential therapeutic avenues.

Activation of cardiomyocyte proliferation in situ is a promising approach for replacing lost cardiomyocytes after injury or disease, such as myocardial infarction (MI) and heart failure.^1^ ncRNAs (noncoding RNAs) play vital roles in cell-cycle regulation, and incorporating them is a powerful strategy for promoting cardiomyocyte proliferation.^2,3^ circRNAs (circular RNAs) are a novel class of ncRNAs that are circularized by joining the 3′ end of the RNA to the 5′ end.^4^ The circular structure maintains their stability and strengthens their miRNA (microRNA)/protein-binding capacity.^5,6^ Thus, circRNAs may be more effective than noncircular RNAs in regulating the cardiac gene regulator network and inducing cardiomyocyte proliferation. Moreover, emerging evidence indicates that circRNAs may play fundamental roles in the regulation of cardiac regeneration.^7^ Certain kinds of circRNAs have been found to control the regulation of cell proliferation, differentiation, development, and even tissue regeneration.^8^ circRNAs also participate in biological processes and pathological conditions of the cardiovascular system, including the development of cardiomyopathy, cardiomyocyte hypertrophy, senescence, and apoptosis.^9–11^ In addition, high-throughput RNA sequencing has recently revealed circRNAs that are frequently differentially expressed between neonatal and adult heart, and these deregulated circRNAs are likely involved in heart regeneration.^12^ Therefore, targeting circRNAs might be an attractive strategy for promoting a cardiac regenerative response because of their important roles in cell proliferation and cardiomyocyte function. However, the role of circRNAs in cardiac regeneration has not been previously investigated.

Identification and validation of the key circRNA function in the acquisition of regenerative capability is also critical for seeking possible therapeutic targets. Recently, circRNAs that are differentially expressed between neonatal and adult hearts have been uncovered by RNA sequencing,^12^ but further recognizing the subgroups essential for cardiac regeneration from a fairly large number of candidates is difficult. Superenhancers are large clusters of active enhancers that are enriched for the binding of key master transcription factors.^13^ These clusters of enhancers are developmental and cell-type specific and act as causal mechanisms of cell differentiation, specification, and development by controlling cell-identity genes or ncRNA expression.^14,15^ Because superenhancers are frequently identified near genes or ncRNAs that are important for controlling cell identity and differentiation, such superenhancers may be used to quickly identify key nodes regulating cell differentiation and development.^14,16,17^ Importantly, superenhancer-associated ncRNAs have been found to drive cardiogenesis and cardiomyocyte differentiation,^18,19^ both of which are key links during cardiomyocyte proliferation. Here, we postulated that superenhancers are likely to mark circRNAs that control cardiomyocyte differentiation and development, thereby helping to identify key circRNAs regulating cardiac regeneration.

In line with this hypothesis, we first performed a comprehensive analysis of the relationship between superenhancers and circRNA networks in hearts. We further identified circRNA Nfix (circNfix) as a cardiomyocyte-enriched circRNA that was regulated by the binding of Meis1 (Meis homeobox 1) to its associated superenhancer in the adult heart. Of crucial importance, downregulation of circNfix promoted adult cardiomyocyte proliferation and cardiac regeneration by rescuing Ybx1 (Y-box binding protein 1) and miR-214 (microRNA-214), significantly decreasing the fibrotic area and promoting functional recovery after MI.

## Methods

The data, methods and materials related to this study are available to other researchers on reasonable request.

### Animal Model Establishment

C57BL/6 mice and SD rats were purchased from Guangdong Medical Laboratory Animal Center. The Cas9 knock-in mouse model was obtained from Shanghai Model Organisms Center, Inc. Mouse MI was performed as described.^20^ All animal experiments were approved by the Animal Research Committee of Southern Medical University and performed in accordance with the National Institutes of Health *Guide for the Care and Use of Laboratory Animals*.

### Cardiomyocyte Isolation and Culture

Neonatal cardiomyocytes were isolated from 1 day-old (P1) and 7-day-old (P7) C57BL/6 mice or P1 SD rats, and adult cardiomyocytes were isolated from ≈8 week-old adult mouse as previously described.^2^

### RNA Quantification

Total RNA from cell or tissue lysates was isolated using TRIzol reagent (R6830-01E.Z.N.A, OMEGA). Quantitative reverse transcription–polymerase chain reaction (QRT-PCR) was performed by using SYBR Green PCR Master Mix (Takara, Dalian, China) in a LightCycler 480 System (Roche, Germany). The primers are listed in Table I in the online-only Data Supplement.

### Immunohistochemical and Immunofluorescent Analysis

After collection, hearts were fixed with 10% formalin or embedded in optimum cutting temperature embedding medium (Sakura) as appropriate. Immunohistochemical and immunofluorescent analyses were performed as previously described.^21^

### Statistical Analysis

Results were analyzed with SPSS 18.0 software. All data are presented as the mean±SD. Unless mentioned otherwise, after normality testing using the Shapiro-Wilk test, Student *t* test (2-group comparison) or 1-way/2-way repeated-measurements ANOVA, followed by Bonferroni post hoc testing (multi-group comparison), were used to examine the difference as appropriate. The survival rate was determined using the Kaplan-Meier method. The difference between survival curves was determined using the log-rank (Mantel-Cox) test. A value of *P*<0.05 was considered statistically significant.

More detailed materials and methods used in this study are provided in the Methods in the online-only Data Supplement.

## Results

### Superenhancers Mark Abundant, Tissue-Specific, and Developmental Stage-Specific circRNAs

Using an enhancer catalog generated in 23 human tissues, defined by the presence of H3K27ac (acetylation of histone H3 lysine 27) marks, we assigned enhancers to the most proximal promoters of circRNAs. A variety of circRNAs were spatially associated with superenhancers and typical enhancers (TEs), which were named superenhancer-associated circRNAs (SE-circRNAs) and typical enhancer–associated circRNAs (TE-circRNAs), respectively. We characterized SE-circRNAs in diverse human tissues, including heart, lung, brain, colon and gastric tissue. SE-circRNAs showed higher expression levels than TE-circRNAs or other circRNAs in these tissues (Figure IA in the online-only Data Supplement). Moreover, SE-circRNAs were more likely to be tissue-type specific than TE-circRNAs (Figure IB and IC in the online-only Data Supplement), and tissue-specific superenhancers are likely to be associated with tissue-specific circRNAs (Figure ID in the online-only Data Supplement).

We next investigated the role of SE-circRNAs and their conservation at different stages of development. The catalog of H3K27Ac superenhancers in 4 mouse organs covering the embryonic and adult stages revealed widespread changes in superenhancer distribution across different developmental stages (Figure IE in the online-only Data Supplement). A gene ontology analysis of proximal genes around embryonic-specific superenhancers in the heart showed that they are related to biological processes characteristic of cardiogenesis and angiogenesis, whereas adult-specific superenhancers are linked to the downregulation of signaling pathways essential for cardiogenesis (Figure IF in the online-only Data Supplement). These results suggested that superenhancers might be critical drivers or inhibitors of cardiac regeneration.

### CircNfix is a Cardiac SE-circRNA That is Enriched in Adult Cardiomyocytes

**Figure 1.**
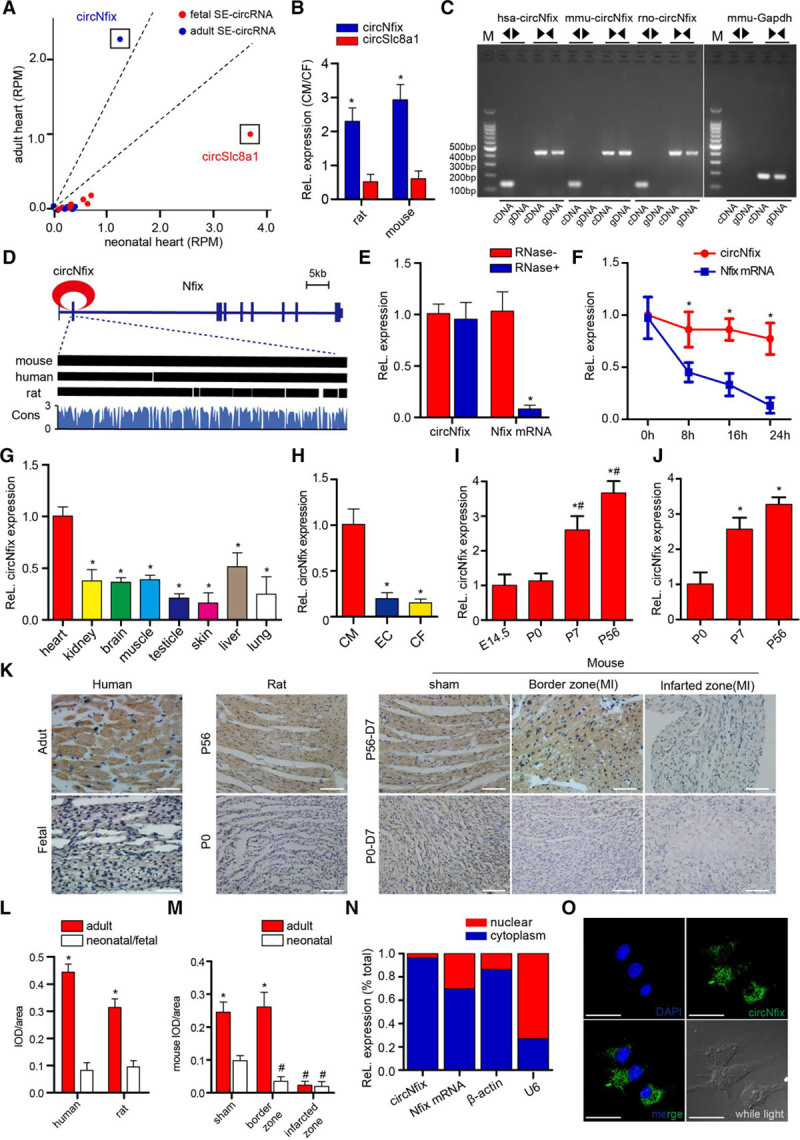
**circNfix is a conserved cardiac-circRNA.** **A**, Superenhancer-associated circRNAs that are differentially expressed in adult and neonatal (P0) rat hearts. circRNA expression is expressed in RPM (reads per million mapped reads). Dashed lines indicate the interval of the ±1.5-fold change; n=3. **B**, circNfix and circSlc8a1 expression in isolated P7 mouse and rat cardiomyocytes (CMs) versus that in cardiac fibroblasts (CFs). n=6; *P<0.05 versus circSlc8a1. **C**, Divergent primers amplify circNfix in cDNA but not genomic DNA (gDNA); divergent and convergent primers are indicated by the direction of the arrow. **D**, The mouse genomic loci of circNfix in Nfix genes. The expression of circNfix was validated by reverse transcription–polymerase chain reaction (RT–PCR) followed by Sanger sequencing. Its conserved analogs in humans and rats, as well as the per base conservation score, were also depicted. **E**, The abundances of circNfix and Nfix mRNA in P0 CMs treated with RNase R, which were normalized to the amount measured in the mock treatment. n=6; *P<0.05 versus RNase- treatment. **F**, The amount of circNfix and Nfix mRNA in P0 CMs treated with actinomycin D at the indicated time points. n=4; *P<0.05 versus Nfix mRNA at each time point. **G**, Quantitative RT–PCR (QRT-PCR) for the abundance of circNfix in multiple adult mouse tissues. n=4; *P<0.05 versus heart. **H**, QRT-PCR assays for detecting circNfix expression in several cardiac cell types isolated from adult mice hearts. n=4; *P<0.05 versus CM. **I**, Gene expression of circNfix in hearts from E14.5, P0, P7, and P56 mice. n=6; *P<0.05 versus E14.5, #*P*<0.05 versus P0. **J**, Gene expression of circNfix in CMs isolated from P0, P7, and P56 mice. n=6; *P<0.05 versus P0. **K** through **M**, Detection of circNfix expression in human, rat and mouse heart sections by in situ hybridization (**K**) and the corresponding quantitative analysis (**L** through **M**). n=2 for adult human hearts, n=6 for other groups; *P<0.05 versus neonatal or fetal sample, #*P*<0.05 versus sham group. **N**, QRT-PCR for the abundance of circNfix and Nfix mRNA in either the cytoplasm or nucleus of P7 CMs. **O**, RNA fluorescense in situ hybridization for circNfix in P7 CMs; bar=50 µm. CF indicates cardiac fibroblast; DAPI, 4′,6-diamidino-2-phenylindole; and EC, endothelial cell.

A previous study identified circRNAs that are differentially expressed between adult and neonatal (P1) rat hearts.^12^ Using this transcriptomic data set, we filtered circRNAs for those that are conserved among humans, rats, and mice (Figure IIA in the online-only Data Supplement). We mapped these murine orthologs to stage-specific heart superenhancers to identify SE-circRNAs (Figures IA and IIA in the online-only Data Supplement). circRNA_Nfix (circNfix) and circRNA_Slc8a1 (circSlc8al) were found to be the predominant SE-circRNAs that are differentially expressed in neonatal and adult hearts (Figure 1A). circNfix was more enriched in cardiomyocytes than was circSlc8a1 (Figure 1B). Moreover, Nfix mRNA expression levels tended to be decreased in adult mouse hearts and cardiomyocytes compared with neonatal mouse hearts and cardiomyocytes, respectively (Figure IIIA in the online-only Data Supplement). The inverse trend for circular and linear transcript expression might indicate an important biological function of circRNA.^22^ Next, we focused on circNfix for detailed expression profiling and functional characterization.

Divergent primers amplified circNfix in cDNA but not in genomic DNA, indicating that this RNA species is circular in form (Figure 1C). The results of the Sanger sequencing analysis of PCR products were in accordance with the circNfix sequence shown in circBase (a database for circRNAs) where the circRNA ID numbers were hsa_circ_0005660 and mmu_circ_0001704. The circNfix nucleotide sequence is strongly conserved, with >95% homology among humans, rats, and mice (Figure 1C; Figure IVA and IVB in the online-only Data Supplement). We then demonstrated that circNfix had greater stability than the linear transcript (Figure 1E and 1F).

QRT-PCR assays showed that circNfix was expressed in several tissues in adult mice and was enriched in the heart (Figure 1G). Moreover, we detected circNfix expression in several cardiac cell types isolated from adult mouse hearts. CircNfix was highly expressed in cardiomyocytes compared with other cardiac cell types (Figure 1H). Importantly, circNfix expression was significantly increased in cardiomyocytes during heart development (Figure 1L through 1M). Furthermore, MI in P0 mice, which induces robust cardiomyocyte proliferation at day 7 after MI, was associated with significant downregulation of circNfix in the border zone (Figure 1K, 1M; Figure VA in the online-only Data Supplement). By contrast, circNfix expression was modestly increased following MI at P56, a time point at which cardiomyocytes have a limited proliferation capacity (Figure 1K, 1M; Figure VA in the online-only Data Supplement). In addition, circNfix was observed to be located mainly in the cytoplasm of P7 cardiomyocytes (Figure 1N, 1O).

### Loss of circNfix Promotes Cardiomyocyte Proliferation in Vitro

**Figure 2.**
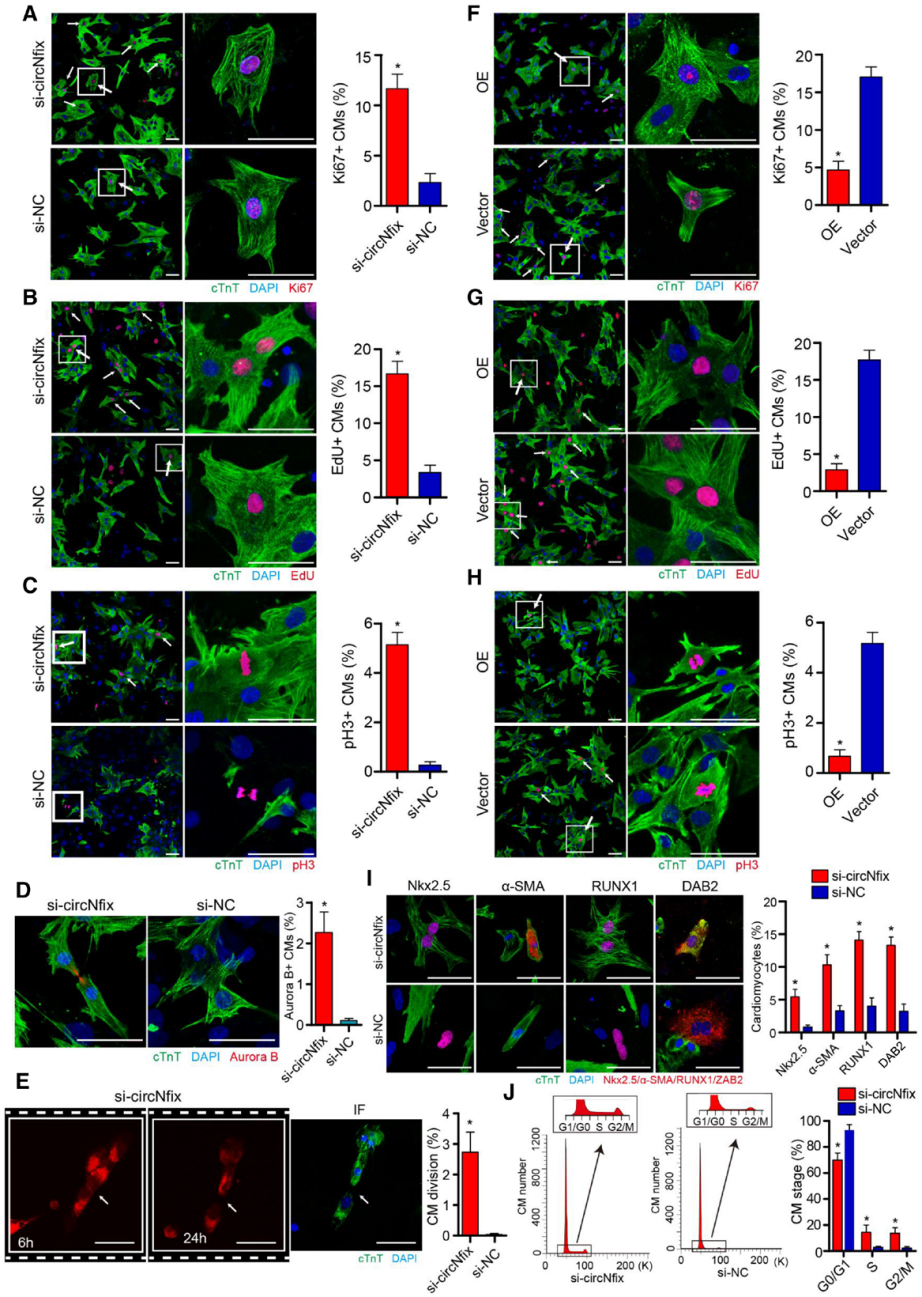
**circNfix downregulation promoted cardiomyocyte (CM) proliferation in vitro.** **A**, Ki67 immunofluorescence staining in isolated P7 CMs transfected with siRNA-circNfix (si-circNfix) or siRNA-negative control (si-NC) and quantification of Ki67-positive CMs (432 CMs from 8 mice in the si-circNfix group and 380 CMs from 8 mice in the si-NC group). Ki67-positive CMs are indicated by arrows. *P<0.05 versus si-NC group; bar=50 µm. **B**, EdU staining in P7 CMs transfected with si-circNfix or si-NC and quantification of EdU-positive CMs (389 CMs from 7 mice in the si-circNfix group and 371 CMs from 7 mice in the si-NC group). EdU-positive CMs are indicated by arrows. *P<0.05 versus si-NC group; bar=50 µm. **C**, PH3 immunofluorescence staining in P7 CMs transfected with si-circNfix or si-NC and quantification of pH3-positive CMs (467 CMs from 8 mice in the si-circNfix group and 461 CMs from 8 mice in the si-NC group). PH3-positive CMs are indicated by arrows. *P<0.05 versus si-NC group; bar=50 µm. **D**, Aurora B immunofluorescence staining in P7 CMs transfected with si-circNfix or si-NC and quantification of Aurora B-positive CMs (413 CMs from 8 mice in the si-circNfix group and 321 CMs from 7 mice in the si-NC group). n=6; *P<0.05 versus si-NC group. Bar=50 µm. **E**, Quantification and representative images from a 24h time-lapse movie of P7 CMs transfected with si-circNfix. The 3rd panel presents immunofluorescence (IF) staining for cTnT in the 24h cells. n=473 CMs from 10 mice in the si-circNfix group and n=450 CMs from 10 mice in the si-NC group; *P<0.05 versus si-NC group. Arrow indicates CMs undergoing cell division; bar=50 µm. **F**, Ki67 immunofluorescence staining in P0 CMs transfected with adenovirus -circNfix (OE) or adenovirus-vector (vector) and quantification of Ki67-positive CMs (408 CMs from 10 mice in the OE group and 317 CMs from 10 mice in the vector group); Ki67-positive CMs are indicated by arrows. *P<0.05 versus vector group; bar=50 µm. **G**, EdU staining in P0 CMs transfected with adenovirus-circNfix or adenovirus-vector and quantification of EdU-positive CMs (428 CMs from 9 mice in the OE group and 331 CMs from 9 mice in the vector group). EdU-positive CMs are indicated by arrows. *P<0.05 versus vector group; bar=50 µm. **H**, PH3 immunofluorescence staining in P0 CMs transfected with adenovirus-circNfix or adenovirus-vector and quantification of pH3-positive CMs (361 CMs from 8 mice in the OE group and 297 CMs from 8 mice in the vector group). PH3-positive CMs are indicated by arrows. *P<0.05 versus vector group; bar=50 µm. **I**, P7 CM dedifferentiation analysis by immunofluorescence staining for Nkx2.5 (359 CMs from 8 mice in the si-circNfix group and 294 CMs from 7 mice in the si-NC group), α-SMA (α-smooth muscle actin; 289 CMs from 7 mice in the si-circNfix group and 245 CMs from 7 mice in the si-NC group), RUNX1 (runt-related transcription factor 1; 367 CMs from 8 mice in the si-circNfix group and 341 CMs from 8 mice in the si-NC group), and DAB2 (285 CMs from 7 mice in the si-circNfix group and 248 CMs from 7 mice in the si-NC group). Dedifferentiated CMs are indicated by arrows. *P<0.05 versus si-NC group; bar=50 µm. **J**, Flow cytometry analysis of P7 CMs transfected with si-circNfix or si-NC. *P<0.05 versus si-NC group, n=10^4 cTnT^+^ cells. DAB2 indicates DAB adaptor protein 2; DAPI, 4′,6-diamidino-2-phenylindole; and Nkx2.5, NK2 homeobox 5.

We used siRNAs (small interfering RNAs) targeting back-splicing of circNfix to specifically knock down circNfix expression in vitro (Figures IIIA through IIID, Table II in the online-only Data Supplement). Notably, knockdown of circNfix significantly increased the percentage of P7 cardiomyocytes expressing cell-cycle activity markers (Figure 2A through 2D; Figure VIA in the online-only Data Supplement). The time-lapse imaging of P7 cardiomyocytes labeled with the fluorescent mitochondrial dye tetramethylrhodamine ethyl ester showed that P7 cardiomyocytes in the si-circNfix group underwent cell division, whereas no cardiomyocyte in the control group was observed to go through cell division (Figure 2E; Movie I and Movie II in the online-only Data Supplement). Moreover, we investigated the effects of circNfix overexpression on P0 cardiomyocyte proliferation. Overexpression of circNfix was found to reduce the ratio of P0 cardiomyocytes expressing proliferation markers (Figure 2F through 2H). We observed that silencing circNfix had no effect on P7 cardiomyocyte proliferation (Figure IIIB through IIIE in the online-only Data Supplement).

Moreover, we observed more rounded cells, with disorganization and a reduced number of sarcomere structures in P7 cardiomyocytes when circNfix was knocked down (Figure VIB in the online-only Data Supplement). This observation was in accordance with an increase in dedifferentiation markers’ expression (Figure 2I). Flow cytometry assays further revealed that more P7 cardiomyocytes were accumulated at the synthesis and gap 2/mitotic phases of the cell cycle. (Figure 2J). In addition, the number of apoptotic cardiomyocytes after H_2_O_2_ treatment was significantly reduced by circNfix silencing, whereas circNfix overexpression increased the number, suggesting that circNfix expression might be associated with cardiomyocyte survival (Figure VIIA and VIIB in the online-only Data Supplement).

### Loss of circNfix Promotes Adult Cardiomyocyte Proliferation in Vivo

**Figure 3.**
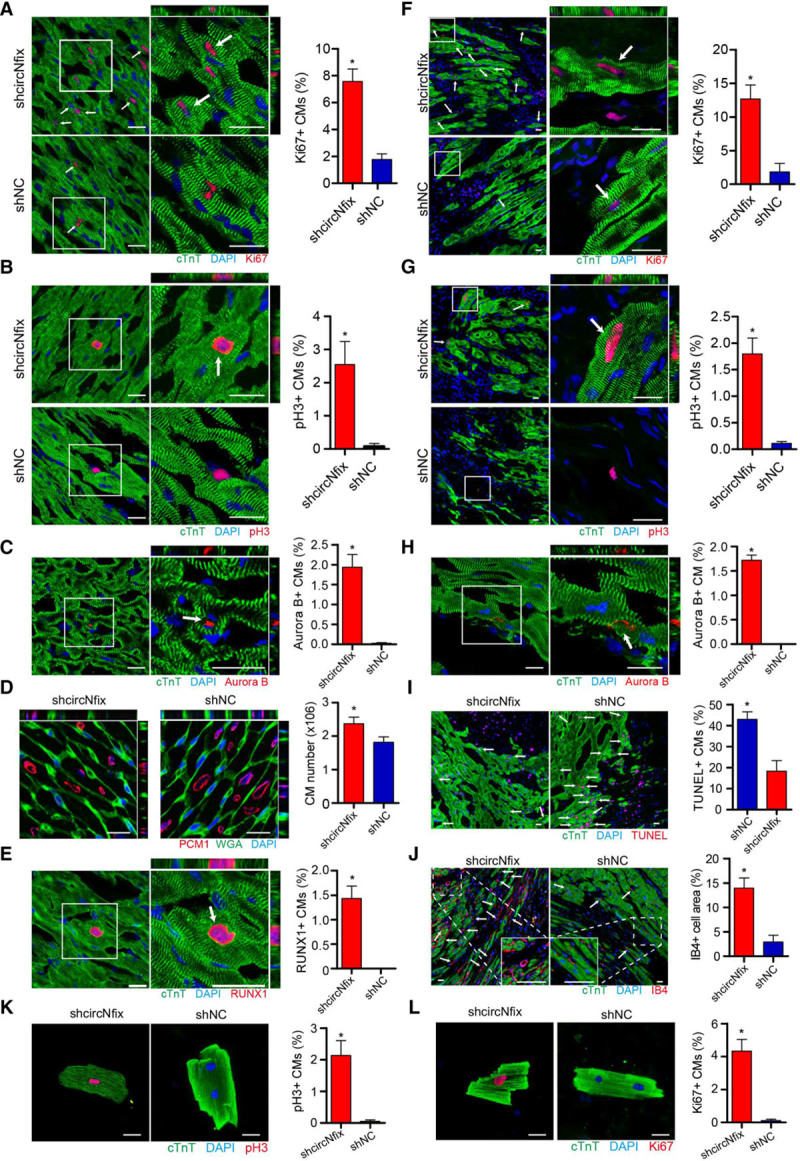
**circNfix downregulation promoted adult cardiomyocyte (CM) proliferation.** **A**, Ki67 immunofluorescence staining in adult mouse hearts 14 days after transfection with AAV9-shRNA-circNfix (adeno-associated virus-9 short-hairpin RNA; shcircNfix) and AAV9-shRNA-NC (shNC) and quantification of Ki67-positive CMs (3051 CMs from 6 mice in the shcircNfix group and 2864 CMs from 6 mice in the shNC group). Ki67-positive CMs are indicated by arrows. *P<0.05 versus shNC group; bar=25 µm. **B**, PH3 immunofluorescence staining in adult mouse hearts 14 days after transfection with AAV9-shcircNfix and AAV9-shNC and quantification of pH3-positive CMs (2919 CMs from 6 mice in the shcircNfix group and 2458 CMs from 6 mice in the shNC group). pH3-positive CMs are indicated by arrows. *P<0.05 versus shNC group; bar=25 µm. **C**, Aurora B immunofluorescence staining in adult mouse hearts 14 days after transfection with AAV9-shcircNfix and AAV9-shNC and quantification of Aurora B-positive CMs (2084 CMs from 6 mice in the shcircNfix group and 1954 CMs from 6 mice in the shNC group). aurora B-positive CMs are indicated by arrows. *P<0.05 versus shNC group; bar=25 µm. **D**, The labeling strategy used to identify cardiomyocyte nuclei and the number of cardiomyocytes per sample. Cardiomyocyte nuclei were labeled with antibodies against PCM-1 (red), and the cardiomyocyte cell borders were labeled with antibodies against WGA (green). n=5 mice; *P<0.05 versus shNC group; bar=25 µm. **E**, Immunofluorescence analysis of RUNX1 (runt-related transcription factor 1) in the shcircNfix and shNC groups 14 days after virus infections (3156 CMs from 6 mice in the shcircNfix group and 3082 CMs from 6 mice in the shNC group). RUNX1-positive CMs are indicated by arrows. *P<0.05 versus shNC group; bar=25 µm. **F**, Ki67 immunofluorescence staining in the shcircNfix and shNC groups 14 days post–myocardial infarction (MI) and quantification of Ki67-positive CMs (3024 CMs from 6 mice in the shcircNfix group and 2947 CMs from 6 mice in the shNC group). Ki67-positive CMs are indicated by arrows. *P<0.05 versus shNC group; bar=25 µm. **G**, PH3 immunofluorescence staining in the shcircNfix and shNC groups 14 days after MI) and quantification of pH3-positive CMs (2896 CMs from 6 mice in the shcircNfix group and 2843 CMs from 6 mice in the shNC group). pH3-positive CMs are indicated by arrows. Bar=25 µm. **H**, Aurora B immunofluorescence staining in the shcircNfix and shNC groups 14 days after MI and quantification of Aurora B-positive CMs (2684 CMs from 6 mice in the shcircNfix group and 2597 CMs from 6 mice in the shNC group). aurora B-positive CMs are indicated by arrows. *P<0.05 versus shNC group; bar=25 µm. **I**, TUNEL immunofluorescence staining in the shcircNfix and shNC groups 7 days post- MI and quantification of TUNEL-positive CMs (2231 CMs from 6 mice in the shcircNfix group and 2195 CMs from 6 mice in the shNC group). TUNEL-positive CMs are indicated by arrows. *P<0.05 versus shNC group; bar=25 µm. **J**, IB4 staining in the shcircNfix and shNC groups 7 days post-MI and quantification of IB4-positive capillaries. IB4 (isolectin B4)-positive capillaries are indicated by arrows. n=6 mice; *P<0.05 versus shNC group; bar=25 µm. **K**, PH3 immunofluorescence staining in adult CMs isolated from the shcircNfix and shNC groups 14 days after virus infection and quantification of pH3-positive CMs (221 CMs from 6 mice in the shcircNfix group and 198 CMs from 6 mice in the shNC group). *P<0.05 versus shNC group; bar=25 µm. **L**, Ki67 immunofluorescence staining in adult CMs isolated from the shcircNfix and shNC groups 14 days after virus infection and quantification of Ki67-positive CMs (218 CMs from 6 mice in the shcircNfix group and 216 CMs from 6 mice in the shNC group). *P<0.05 versus shNC group; Barbar=25 µm. DAPI indicates 4′,6-diamidino-2-phenylindole.

To assess the in vivo effect of circNfix downregulation, we delivered AAV9-shRNA (adeno-associated virus-9 short hairpin RNA; circNfix) to adult mice and detected the proliferation of cardiomyocytes. The transfection efficiency of AAV9-shcircNfix-GFP (green fluorescent protein) was ≈80% by GFP and cardiomyocyte marker costaining, and circNfix expression was significantly reduced (Figure VIIIA through VIIIF in the online-only Data Supplement). We also demonstrated the specificity of AAV9-mediated circNfix knockdown for cardiomyocytes (Figure IXA through IXF in the online-only Data Supplement). In addition, knockdown of circNfix was observed to markedly elevate the ratio of proliferative cardiomyocytes and increase the total cardiomyocyte number (Figure IIIA through IIID, Figure XA in the online-only Data Supplement). Moreover, QRT-PCR assays showed that circNfix downregulation significantly increased the expression of the dedifferentiation markers in vivo (Figure XIA in the online-only Data Supplement). Western blotting and immunofluorescence analysis further confirmed that circNfix knockdown increased RUNX1 (runt-related transcription factor 1) expression in adult hearts (Figure 3E; Figure XIB in the online-only Data Supplement). Additionally, circNfix downregulation resulted in transient sarcomere disassembly and reduction in vivo (Figure XIC in the online-only Data Supplement). Disrupted cardiomyocyte adhesion, suggested by the disordered arrangement of cardiomyocytes and enlarged intracellular space, further confirmed the transient dedifferentiation of adult cardiomyocytes (Figure XID in the online-only Data Supplement). All the above results indicated that loss of circNfix was able to induce cardiomyocyte dedifferentiation and proliferation followed by redifferentiation.

Moreover, we isolated cardiomyocytes from adult mouse hearts injected with AAV9-shcircNfix or AAV9-shNC. Ex vivo studies showed that total cardiomyocyte number and the percentages of pH3^+^ (phosphorylated histone H3 positive), Ki67^+^ (marker of proliferation Ki-67 positive), and mononuclear cardiomyocytes were significantly higher in the AAV9-shcircNfix group than in the AAV9-shNC group (Figure 3K, 3L; Figure XIIA in the online-only Data Supplement). The expression of dedifferentiation markers was also significantly higher in the AAV9-shcircNfix group than in the AAV9-shNC group (Figure XIIA through XIIE in the online-only Data Supplement). We also found that circNfix loss resulted in transient sarcomere disassembly and reduction in isolated adult cardiomyocytes (Figure XIIB through XIIE in the online-only Data Supplement). Additionally, downregulation of circNfix had no effect on cardiac fibroblast proliferation (Figure XIIIA in the online-only Data Supplement).

CRISPR/Cas9 technology was used to knock out circNfix within cardiomyocytes both in vitro and in vivo. We successfully constructed a circNfix knockdown cell line using HL-1, a mouse cardiomyocyte cell line, with the CRISPR/Cas9 method as previously described^23^ (Figure XIVA and XIVB,; Table III in the online-only Data Supplement). Deletion of circNfix was observed to increase the proliferation of HL-1 cardiomyocytes (Figure XIVC and XIVD in the online-only Data Supplement). Moreover, we successfully decreased circNfix expression in vivo using CRISPR/Cas9 knock-in mice, inducing a significant increase in cardiomyocyte proliferation in these circNfix-deficient mice (Figure XVA through XVK, Table III in the online-only Data Supplement).

### Loss of circNfix Promotes Adult Cardiac Regeneration After MI

Next we studied whether knockdown of circNfix could elevate myocardial repair and preserve cardiac function after MI. Knockdown of circNfix was performed in an MI model by injection of AAV9-shcircNfix or AAV9-shNC in the peri-infarcted area. As expected, knockdown of circNfix markedly increased the ratio of proliferative cardiomyocytes in the infarct border zone at 14 days post-MI (Figure 3F through 3H; Figures XB and XVIA in the online-only Data Supplement).

We observed a 2.4-fold decrease in apoptotic cardiomyocytes in the AAV9-shcircNfix group at 7 days post-MI compared with the control group (Figure 3I). We found circNfix knockdown increased IB4^−^ (isolectin B4 negative), CD31^−^ (cluster of differentiation 31 negative), and vWF (von Willebrand Factor)-stained vessel density in the infarcted or border area (Figure 3I; Figure XVIIA through XVIID, and XVIIF in the online-only Data Supplement). We also observed a markedly increased proliferative rate of cardiac endothelial cells after circNfix knockdown in vivo, using cTnT (cardiac troponin)/CD31/Ki67 triple-staining on heart sections (Figure XVIIE and XVIIG in the online-only Data Supplement). Immunofluorescence analysis of α-SMA (α-smooth muscle actin) and CD31 revealed that arteriolar density was significantly greater in the AAV9-shcircNfix group than in the AAV9-shNC group (Figure XVIIIA and XVIIIB in the online-only Data Supplement). These results suggested increased angiogenesis during circNfix knockdown, inducing cardiac regenerative processes.

**Figure 4.**
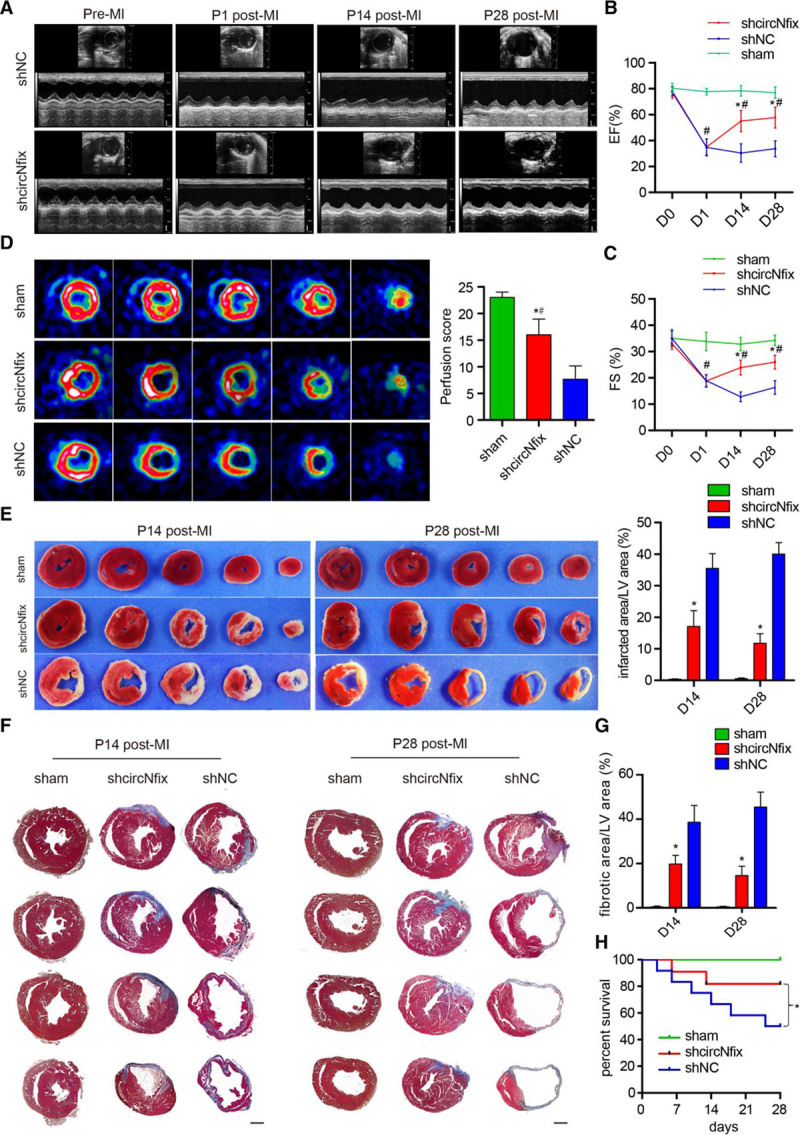
**Downregulation of circNfix induces cardiac regeneration after myocardial infarction (MI).** **A**, Cardiac function analyzed by echocardiography pre-MI and 1, 14, and 28 days post-MI. Time stamps, 33 ms, *x* axis; bar=1 mm, *y* axis. **B** and **C**, Quantitative analysis of ejection fraction (EF) and fractional shortening (FS). n=10 per group; *P<0.05 versus shNC, #*P*<0.05 versus sham at each time point. **D**, Positron emission tomography–computed tomography of sham, shcircNfix-transfected, and shNC-transfected mouse hearts 28 days post-MI. n=4 per group; *P<0.05 versus shNC, #*P*<0.05 versus sham. **E**, Triphenyltetrazolium chloride staining of mouse ventricular cross-sections at 14 and 28 days post-MI, *P<0.05 versus shNC, #*P*<0.05 versus sham; n=5 per group. **F**, Representative images of Masson trichrome-stained heart section at 14 and 28 days post-MI. Serial sectioning was performed at 500 µm intervals; Barbar=1 mm. **G**, Quantitative analysis of the fibrotic areas in heart sections, *P<0.05 versus shNC, #*P*<0.05 versus sham; n=8 per group. **H**, Kaplan-Meier survival curves in the shcircNfix and shNC groups post-MI (n=30 mice per group).

We further explored the effect of circNfix silencing on functional recovery after MI. Echocardiography revealed that ejection fraction and fractional shortening of cardiac functions were significantly preserved in the AAV9-shcircNfix groups compared with the control group (Figure 4A through 4C). Positron emission tomography–computed tomography, TTC (triphenyltetrazolium chloride) staining, and Masson trichrome staining revealed that the infarcted area and cardiac fibrosis were significantly reduced in the AAV9-shcircNfix group compared with the control group (Figure 4D through 4G). These responses are associated with a significant increase in survival rate after MI in the circNfix knockdown group compared with that in the control group (Figure 4H).

### Overexpression of circNfix Inhibits Neonatal Cardiomyocyte Proliferation in Vivo

**Figure 5.**
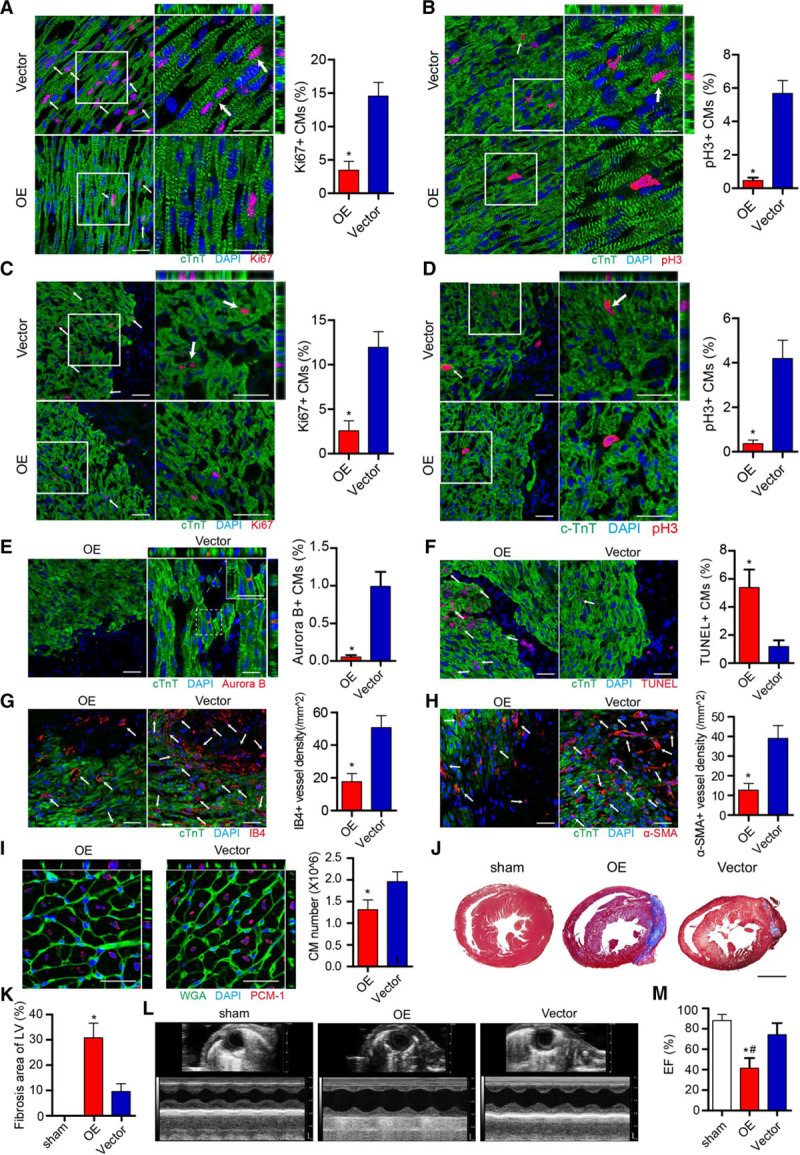
**circNfix overexpression inhibits neonatal cardiac regeneration post–-myocardial infarction (MI).** **A**, Ki67 immunofluorescence staining in P0 neonatal mouse hearts 4 days after transfection with adenovirus-circNfix (OE) and adenovirus-vector (vector) and quantification of Ki67-positive cardiomyocytes (CMs; 2015 CMs from 6 mice in the OE group and 2149 CMs from 6 mice in the vector group). Ki67-positive CMs are indicated by arrows. *P<0.05; bar=25 µm. **B**, PH3 immunofluorescence staining in the OE and vector groups and quantification of pH3-positive CMs (2919 CMs from 6 mice in the OE group and 2458 CMs from 6 mice in the vector group). pH3-positive CMs are indicated by arrows. *P<0.05; bar=25 µm. **C**, Ki67 immunofluorescence staining in the OE and vector groups 7 days post-MI and quantification of Ki67-positive CMs (2015 CMs from 6 mice in the OE group and 2149 CMs from 6 mice in the Vector group). Ki67-positive CMs are indicated by arrows. *P<0.05; bar=25 µm. **D**, PH3 immunofluorescence staining in the OE and vector groups 7 days post-MI and quantification of pH3-positive CMs (2896 CMs from 6 mice in the OE group and 2843 CMs from 6 mice in the vector group). pH3-positive CMs are indicated by arrows. *P<0.05; bar=25 µm. **E**, Aurora B immunofluorescence staining in the OE and vector groups 7 days post-MI and quantification of Aurora B-positive CMs (2684 CMs from 6 mice in the OE group and 2597 CMs from 6 mice in the vector group). *P<0.05; bar=25 µm. **F**, TUNEL immunofluorescence staining in the OE and vector groups 7 days post-MI and quantification of TUNEL-positive CMs (2231 CMs from 6 mice in the OE group and 2195 CMs from 6 mice in the vector group). TUNEL-positive CMs are indicated by arrows. *P<0.05; bar=25 µm. **G**, IB4 staining in the OE and vector groups 7 days after MI and quantification of IB4-positive capillaries, IB4-positive capillaries are indicated by arrows. n=6; *P<0.05; bar=25 µm. **H**, α-SMA (α-smooth muscle actin) staining in the OE and vector groups 7 days post-MI and quantification of α-SMA-positive capillaries, α-SMA-positive capillaries are indicated by arrows. n=6; *P<0.05; bar=25 µm. **I**, The labeling strategy used to identify cardiomyocyte nuclei and the number of cardiomyocytes in the OE and vector groups 7 days post-adenovirus transfection. Cardiomyocyte nuclei were labeled with antibodies against PCM-1 (red), and the cardiomyocyte cell borders were labeled with antibodies against WGA.n=6; *P<0.05; bar=25 µm. **J** and **K**, Masson staining of mouse ventricular cross-sections in the OE and vector groups 7 days post-MI and quantitative analyses of infarct size (n=6 mice per group). *P<0.05; bar=1 mm. **L**, Cardiac function analyzed by echocardiography for the sham, OE, and vector groups 7 days post-MI (time stamps, 33 ms, *x* axis; bar=1 mm, *y* axis). **M**, Quantitative analysis of ejection fraction (EF) in the OE and vector groups 7 days post-MI. n=7 per group; *P<0.05 versus OE, #*P*<0.05 versus sham. DAPI indicates 4′,6-diamidino-2-phenylindole; PCM-1, pericentriolar material 1; and WGA, wheat germ agglutinin.

To determine whether forced circNfix overexpression impairs neonatal cardiomyocyte proliferation and cardiac regeneration, we injected adenovirus-expressing circNfix or control vector into P1 mouse hearts. We observed a high transfection efficiency and specificity of adenovirus -circNfix for cardiomyocytes (Figures XIXA through XIXE and XXA through XXI in the online-only Data Supplement). Furthermore, circNfix overexpression significantly reduced the proportion of proliferative cardiomyocytes and the total cardiomyocyte number (Figure 5A through 5I). In addition, the percentage of TUNEL^+^ cardiomyocytes in the infarct border zone was significantly higher in mice overexpressing circNfix than in the control mice (Figure 5F). IB4, α-SMA, and CD31 immunostaining showed that circNfix overexpression inhibited angiogenesis after P1 mouse MI (Figure 5G and 5H; Figure XXIA in the online-only Data Supplement). Accordingly, echocardiographic ejection fraction at 7 days after MI was significantly lowered in the adenovirus -circNfix group compared with the control group (Figure 5L and 5M). Histological analysis showed a significant increase in the infarct size in circNfix-overexpressing mice compared with that in the control mice (Figure 5J and 5K). Collectively, these findings demonstrated that repression of cardiomyocyte proliferation by circNfix overexpression could impair cardiac regenerative repair in a neonatal mouse model.

We also assessed the effect of circNfix on the regulation of cardiomyocyte hypertrophy. Neither circNfix silencing nor circNfix overexpression was found to significantly alter cardiomyocyte size in vivo or cardiomyocyte volume ex vivo (Figure XXIIA through XXIID in the online-only Data Supplement).

### Meis1 Controls circNfix Expression by Binding to the Superenhancer at the Nfix Locus

**Figure 6.**
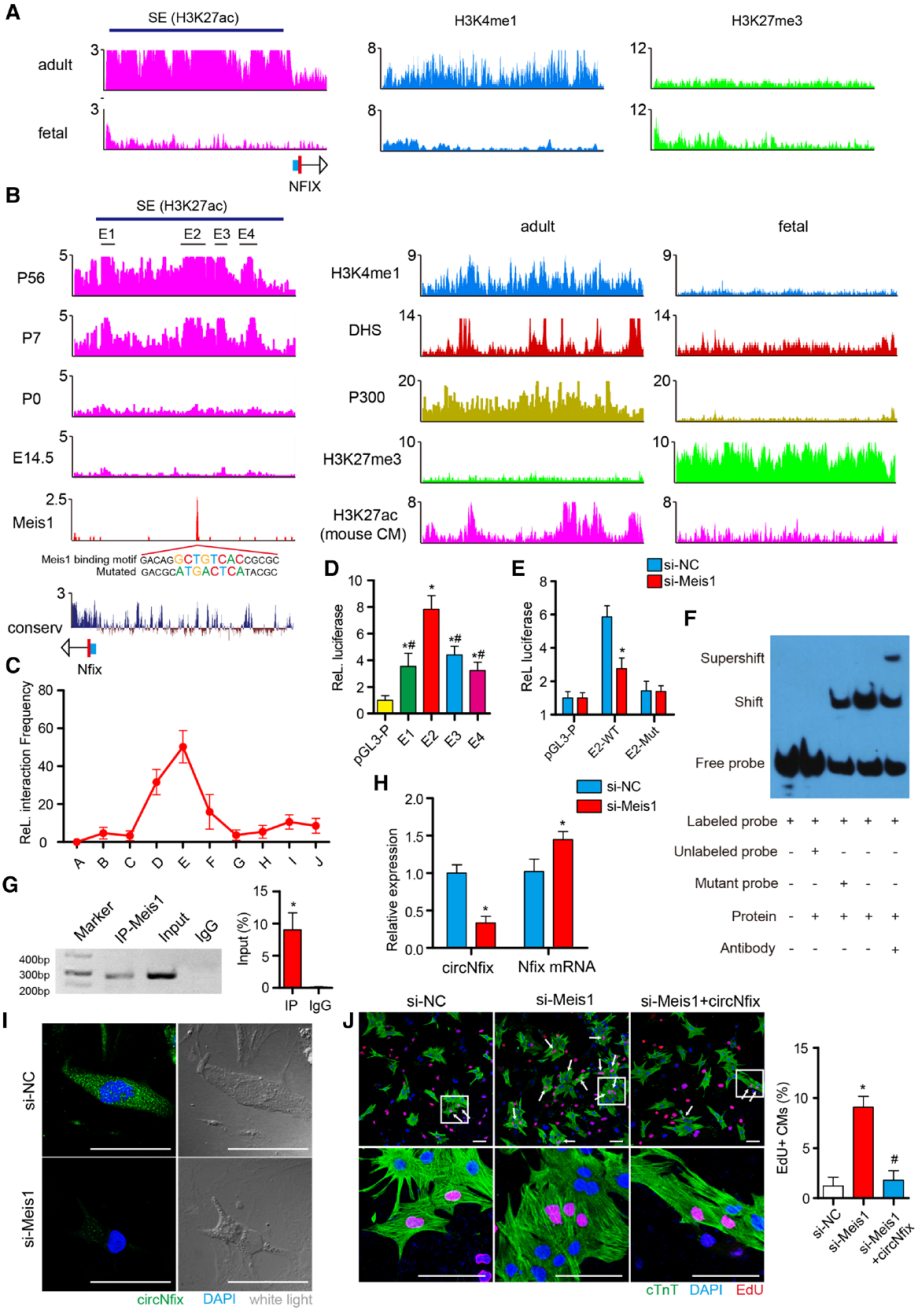
**Meis1 (Meis homeobox 1) drives circNfix expression by binding to an superenhancer (SE) at the Nfix locus.** **A**, Chromatin immunoprecipitation–sequence (ChIP-seq) profiles of H3k27ac, H3k4me1 and H3k27me3 at the Nfix locus in human heart tissues. Predicted superenhancers are depicted as blue bars. The *y* axis indicates reads per million. **B**, ChIP-seq profiles of H3k27ac, H3k4me1, H3k27me3, P300, DNAse hypersensitivity (DHS), Meis1, and phylogenetic analysis with space/time models conservation score at the Nfix locus in mouse heart tissues, as well as the ChIP-seq profile of H3k27ac in mouse cardiomyocytes (CMs). Predicted SEs are depicted as blue bars. The mutated sequence is related to pGL3-E2-mut that was used in Figure 6E. **C**, 3C-qPCR (quantitative polymerase chain reaction) analysis of the looping events between the superenhancer and circNfix promoter region. The relative interaction frequency was normalized to the closest Mbo1 digestion site. The *x* axis shows the primers used whose locations are presented in Figure IXC in the online-only Data Supplement. **D**, Luciferase assay of the pGL3-promoter reporter vector constructed with 4 constituent enhancers (E1, E2, E3, and E4). n=5; *P<0.05 versus pGL3-p, #*P*<0.05 versus E2. **E**, The luciferase activities of wild-type E2 and mutated E2 were measured in P7 CMs on Meis1 silencing. The mutated Meis1 binding sites are presented in Figure 6B. n=5; *P<0.05 versus si-NC. **F**, Electrophoretic mobility shift assay result for nuclear proteins extracted from P7 CMs after incubation with individual DIG-ddUTP-labeled oligonucleotide probes of the Meis1 binding site in circNfix-SE. **G**, ChIP–quantitative PCR assay showing amplification of the Meis1 binding site in circNfix-SE using an antibody against Meis1. n=4; *P<0.05 versus IgG. **H**, Quantitative reverse transcription–polymerase chain reaction for circNfix and Nfix mRNA in P7 CMs on Meis1 knockdown. n=5; *P<0.05 versus si-NC. **I**, RNA fluorescense in situ hybridization for circNfix in P7 CMs on Meis1 knockdown; bar=50 µm. **J**, EdU staining in P7 CMs after Meis1 and circNfix interference. n=6; *P<0.05 versus si-NC, #*P*<0.05 versus si-Meis1; bar=50 µm.

The cardiac superenhancer at the Nfix locus is present in human and mouse hearts (Figure 6A and 6B). We validated that this superenhancer region is enriched in other superenhancer marks, including H3K4me1 (monomethylation of histone H3 at lysine 4), H3K27me3 (trymethylization of histone H3 at lysine 27), P300, and DNase hypersensitive site, using ChIP-sequence (ChIP-seq) data (Figure 6A and 6B). Consistent with the increase in circNfix expression in adult hearts, active histone marks at the Nfix locus were markedly increased, whereas inactive histone marks were reduced in adult hearts (Figure 6A and 6B). We also found that the superenhancer from which circNfix is derived is uniquely active in adult heart tissue compared with other tissues (Figure XXIIIA and XXIIIB in the online-only Data Supplement). Importantly, the enhancer H3K27ac histone marks were also noticeably increased in adult cardiomyocytes compared with embryonic cardiomyocytes (Figure 6B). Superenhancers often recruit transcription factors to fulfill their enhancer activity. We therefore used ChIP-seq to map the genome-wide occupancy of Meis1, a transcription factor controlling the cell-cycle arrest of adult cardiomyocytes, inside the circNfix-associated superenhancer. The ChIP-seq profile of Meis1 revealed a prominent peak that overlapped with circNfix-SE (Figure 6B). Moreover, a Meis1 motif was observed in the circNfix-associated superenhancer sequence, which is highly conserved among species (Figure 6B). Chromosome 3C assays showed chromatin looping between the circNfix promoter and superenhancer, fully supporting the superenhancer as a regulatory element for circNfix (Figure 6C; Figure XXIIIC and Table IV in the online-only Data Supplement). We next cloned individual constituent enhancers (E1, E2, E3, E4) of circNfix-SE into enhancer-reporter vectors and measured their activities by using a luciferase reporter system. E2 was shown to be strongly active in cardiomyocytes (Figure 6D). Using luciferase, electrophoretic mobility shift assay and ChIP–quantitative PCR assays, we confirmed that Meis1 bound to E2 in P7 cardiomyocytes (Figure 6F and 6G). Moreover, Meis1 silencing significantly reduced circNfix expression, although it increased Nfix mRNA abundance (Figure 6H and 6I). We further observed that Meis1 silencing increased the percentage of P7 cardiomyocytes exhibiting proliferation kinetics indexes, and these effects could be abrogated by circNfix overexpression (Figure 6J).

We used CRISPR-Cas9 knock-in mice to generate loss-of-function mutations in the Meis1 gene as previously described (Figure XXIVA through XXIVD, Table III in the online-only Data Supplement).^10,24^ These Meis1 mutations significantly decreased circNfix expression and increased cardiomyocyte proliferation in vivo, which could be reversed by AAV9-mediated circNfix overexpression (Figure XXVA through XXVG in the online-only Data Supplement). We also demonstrated that circNfix knockdown could reverse the inhibitory effect of Meis1 on cardiomyocyte proliferation (Figure XXVIA through XXVIF in the online-only Data Supplement). Additionally, we failed to observe any synergistic effect between Meis1 and circNfix knockdown (Figures XXVA through XXVG, and XXVIIA through XXVIID in the online-only Data Supplement). The above results indicated that circNfix expression is tightly regulated by the interaction between Meis1 and circNfix_SE.

### circNfix Promotes Ybx1 Degradation via Ubiquitination-Proteasome Pathways

**Figure 7.**
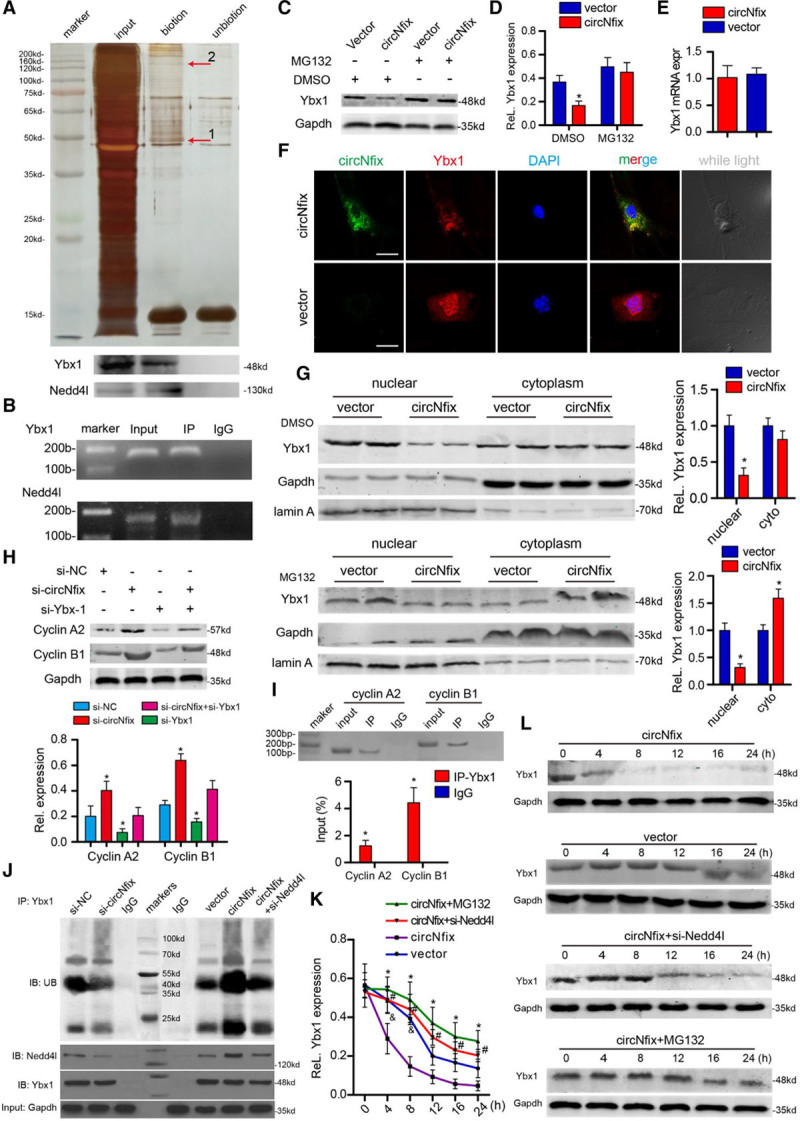
**circNfix binds to Ybx1 (Y-box binding protein 1) and promotes its degradation.** **A**, Protein immunoprecipitated by the probe against circNfix and the control. The numbers indicate the region of the gel excised for mass spectrum determination. **Bottom**, Ybx1 and Nedd4l (an E3 ubiquitin ligase) protein were detected by Western blotting. Biotin: biotinylated probe targeting the circNfix back-spliced site; Unbiotin: unbiotinylated probe. **B**, RNA binding protein immunoprecipitation experiments were performed using Ybx1, Nedd4l, or negative IgG antibody. The purified RNA was used for quantitative reverse transcription–polymerase chain reaction (QRT-PCR) assays. n=5; *P*<0.05 versus IgG. **C** and **D**, Ybx1 protein levels in P0 cardiomyocytes transfected with adenovirus-circNfix (circNfix) or adenovirus-empty vector (vector). n=5; *P<0.05 versus vector. **E**, Ybx1 mRNA expression level in P0 cardiomyocytes detected by QRT-PCR assays; n=5. **F**, RNA fluorescense in situ hybridization showed colocalization of circNfix and Ybx1 in P0 cardiomyocytes with and without circNfix overexpression. Bar=50 µm. **G**, Fractionation assays showed that circNfix overexpression promoted cytoplasmic translocation of Ybx1 and increased Ybx1 degradation in the cytoplasm. n=5; *P<0.05 versus vector. **H**, Cyclin A2 and cyclin B1 protein levels after circNfix and Ybx1 interference. n=6, *P<0.05 versus si-NC. **I**, Chromatin immunoprecipitation quantitative PCR assay showing amplification of the Ybx1 binding site in cyclin A2 and cyclin B1 promoters using an antibody against Ybx1. n=4; *P<0.05 versus IgG. **J**, Cell lysates were immunoprecipitated with antibody against Ybx1 and analyzed by immunoblotting with ubiquitin (Ub)-specific antibody, anti-Ybx1 antibody, or anti-Nedd4l antibody. **Bottom**, Input from cell lysates. **K** and **L**, Ybx1 protein expression level in cardiomyocytes that overexpressed circNfix and were treated with MG132 (20 µg/mL), si-Nedd4l or control treatment or the indicated periods of time after 20 μg/ml cycloheximide treatment. *P<0.05 circNfix+MG132 versus circNfix, #*P*<0.05 circNfix+si-Nedd4l versus circNfix, &*P*<0.05 vector versus circNfix at each time point; n=4. DAPI indicates 4′,6-diamidino-2-phenylindole; and DMSO, dimethyl sulfoxide.

To better explore the function of circNfix in cardiomyocytes, we performed RNA pulldown assays of circNfix using biotinylated probes targeting the circNfix back-spliced sequence (Figure 7A; Figure XXVIIIA in the online-only Data Supplement), and the precipitates were subjected to mass spectrometry analysis. Several RNA-binding proteins were identified by mass spectrometry analysis, and the most abundant among them was Ybx1 (Table V in the online-only Data Supplement). Bioinformatics analysis revealed that Ybx1 and circNfix are likely to interact (Figure XXVIIIB in the online-only Data Supplement), and several Ybx1 motifs were observed in the circNfix sequence (Figure XXVIIIC in the online-only Data Supplement). We then confirmed the interaction between circNfix and Ybx1 using RNA pulldown followed by Western blotting and radioimmune precipitation (Figure 7A and 7B).

We next investigated the molecular consequence of the interaction between circNfix and Ybx1. CircNfix knockdown significantly decreased Ybx1 protein expression in cardiomyocytes, which could be attenuated by proteasome inhibitor MG132 (Figure 7C and 7D,; Figure XXVIIIE in the online-only Data Supplement). Ybx1 mRNA expression levels were not altered when circNfix was overexpressed (Figure 7E). Moreover, circNfix inhibited nuclear translocation of Ybx1 in P0 cardiomyocytes (Figure 7F and 7G).

**Figure 8.**
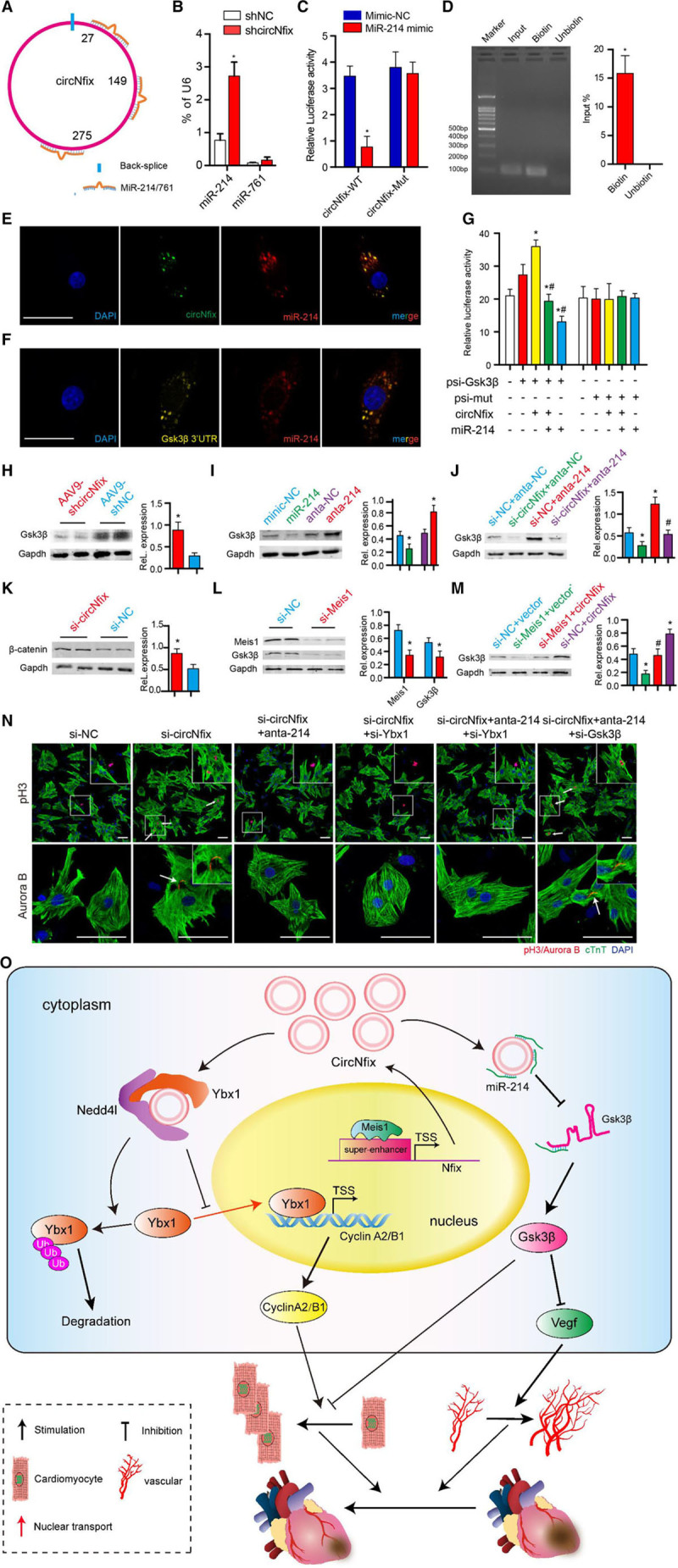
**Interaction of circNfix and miR-214.** **A**, The predicted binding of miR-214 and miR-761 on circNfix. **B**, Expression of miR-214 and miR-761 after knockdown of circNfix in adult mouse hearts. n=5; *P<0.05 versus shNC. **C**, Luciferase activity of cardiomyocytes transfected with luciferase-circNfix-wt or luciferase-circNfix-mut. n=5; *P<0.05 versus Mimic-NC. The mutated sequences are shown in Figure XIA in the online-only Data Supplement. **D**, Coprecipitation of circNfix and miR-214 by RNA pulldown assay. Input group (10% of samples were loaded); targeting the circNfix back-spliced site with a biotinylated probe (Biotin) or an unbiotinylated probe (Unbiotin) (100% of samples were loaded). n=5; *P<0.05. **E**, Colocalization of miR-124 and circNfix was observed by RNA fluorescense in situ hybridization in P0 cardiomyocytes; bar=50 µm. **F**, Colocalization of miR-124 and Gsk3β (glycogen synthase kinase 3 β) 3′UTR was observed by RNA flurescense in situ hybridization in P0 cardiomyocytes; bar=50 µm. **G**, Luciferase activity of cardiomyocytes transfected with luciferase-Gsk3β 3′ UTR-wt or luciferase-Gsk3β 3′ UTR-mut. n=5; *P<0.05 versus Mimic-NC. The mutated sequences are shown in Figure XIC in the online-only Data Supplement. **H**, Western blotting and quantitative analyses of Gsk3β protein levels in hearts infected with AAV9 (adeno-associated virus-9)-shcircNfix and AAV9-shNC, n=4 mice per group, *P<0.05 versus AAV9-shNC. **I**, Gsk3β (glycogen synthase kinase 3 β) protein level after inhibition and overexpression of miR-214. n=6; *P<0.05 versus mimic-NC or inhibitor NC. **J**, GSK3β protein level after circNfix and miR-214 interference (anta-214) in cardiomyocytes. n=5; *P<0.05 versus si-NC+anta-NC group, #*P*<0.05 versus si-NC+anta-214 group. **K**, β-Catenin expression in cardiomyocytes transfected with siRNA (small interfering RNAs)-circNfix (si-circNfix) or siRNA-NC (si-NC). *P<0.05; n=6. **L**, Meis1 (Meis homeobox 1) and Gsk3β expression in cardiomyocytes transfected with siRNA-Meis1 (si-Meis1) or si-NC. n=5; *P<0.05 versus si-NC. **M**, GSK3β protein level after Meis1 and circNfix interference in cardiomyocytes. n=6; *P<0.05 versus si-NC+vector group; #*P*<0.05 versus si-NC+circNfix group. **N**, Immunostaining for pH3 and Aurora B in P7 cardiomyocytes after circNfix, Ybx1, miR-214, and Gsk3β interference. White arrows indicate pH3-positive or Aurora-positive cardiomyocytes. The results of statistical analysis are shown in Figure XXIXC in the online-only Data Supplement. **O**, Illustration of how circNfix regulates cardiac regeneration by binding to Ybx1 (Y-box binding protein 1) and miR-214. Nedd4I indicates an E3 ubiquitin ligase; TSS, transcription start site; and Vegf, vascular endothelial growth factor.

Nuclear Ybx1 has been reported to bind to the promoters of Ccna2 (cyclin A2) and Ccnb1 (cyclin B1) and activate their transcription.^25^ We also found that Ybx1 knockdown could markedly decrease Ccna2 and Ccnb1 expression, which could abrogate circNfix knockdown-induced overexpression (Figure 7G). Subsequent ChIP–quantitative PCR assay confirmed the interactions of Ybx1 with the promoters of Ccna2 and Ccnb1 (Figure 7I). Moreover, Ybx1 overexpression significantly increased cardiomyocyte proliferation (Figure XXVIIID in the online-only Data Supplement), whereas Ybx1 silencing could block circNfix knockdown-induced cardiomyocyte proliferation (Figure 8N; Figure XXIXA through XXIXC in the online-only Data Supplement).

Furthermore, circNfix knockdown and overexpression could reduce and increase the ubiquitination of Ybx1 in cardiomyocytes, respectively (Figure 7J). Moreover, Nedd4l, an E3 ubiquitin ligase that interacts with its substrates for ubiquitination, was also among the proteins identified by mass spectrometry analysis following circNfix RNA pulldown (Table VI in the online-only Data Supplement). Notably, a protein docking program predicted that Ybx1 and Nedd4l were likely to interact (Figure XXVIIIF in the online-only Data Supplement), and coimmunoprecipitation assays confirmed the interaction between Ybx1 and Nedd4l (Figure 7J). Next, we observed the association of circNfix with Nedd4l by using RNA pulldown and radioimmune precipitation assays (Figure 7A and 7B). The altered expression of circNfix did not affect Nedd4l expression (Figure XXVIIIG in the online-only Data Supplement) but significantly affected the interaction of Ybx1 with Nedd4l (Figure 7J). We further confirmed that silencing of Nedd4l increased Ybx1 protein stability by decreasing ubiquitination, which could abrogate the effect of circNfix overexpression (Figure 7K and 7L). Collectively, these results indicated that the inhibitory effect of circNfix on cardiomyocyte proliferation may be achieved by enhancing Ybx1 ubiquitin-dependent degradation.

### circNfix Acts as a Sponge for miR-214 in Cardiomyocytes

We then explored whether circNfix functions as a miRNA sponge to regulate gene expression. Bioinformatics prediction analysis using the Segal Laboratory program showed that several miRNAs may interact with circNfix. Among them, miR-214/761 has the most binding sites on circNfix, reaching 3 conserved binding sites (Figure 8A; Figure XXXA in the online-only Data Supplement). QRT-PCR assays showed that miR-214 had much higher expression levels in the myocardium than miR-761 had (Figure 8B). CircNfix downregulation significantly increased miR-214 gene expression levels (Figure 8B). The results of luciferase, RNA pulldown, and RNA fluorescense in situ hybridization assays confirmed the binding of miR-214 on circNfix (Figure 8C through 8E).

Next, we explored the effects of miR-214 mimic transfection on several candidates that have been associated with cardiac regeneration (Figure XXXB in the online-only Data Supplement). QRT-PCR revealed that the miR-214 mimic significantly decreased Gsk3β (glycogen synthase kinase 3 β) expression (Figure XXXB in the online-only Data Supplement). Furthermore, the binding site of miR-214 in the 3′UTR of Gsk3β is highly conserved across species (Figure XXXC in the online-only Data Supplement), suggesting Gsk3β as a common target gene of miR-214. Consistently, RNA fluorescense in situ hybridization experiments in cardiomyocytes showed overlap between miR-214 and Gsk3β 3′UTR (Figure 8F). The 3′UTR of the Gsk3β gene was also cloned into a luciferase vector and transfected into cardiomyocytes. Transfection with miR-214 mimic significantly reduced luciferase activity, although mutation of the miR-214 binding site eliminated this reduction (Figure 8G; Figure XXXC in the online-only Data Supplement). In addition, circNfix overexpression increased luciferase activity, which could by abrogated by miR-214 mimic transfection (Figure 8G). Similarly, Gsk3β expression was significantly decreased in hearts transfected with AAV9-shcircNfix compared with that in hearts transfected with AAV9-shNC (Figure 8H). Gsk3β expression was significantly reduced by miR-214 mimics but increased by miR-214 inhibitors (Figure 8I). A miR-214 inhibitor abolished the repressive effect of circNfix downregulation on Gsk3β (Figure 8J). Gsk3β is known to regulate cardiomyocyte proliferation via degrading β-catenin.^26^ Consistently, we also found that β-catenin expression was significantly reduced in cardiomyocytes transfected with siRNA-circNfix (Figure 8K). In addition, we did not find an obvious interaction between Ybx1 and miR-214 (Figure XXXD and XXXE in the online-only Data Supplement). Moreover, Meis1 knockdown significantly repressed Gsk3β expression (Figure 8L), which was reversed by circNfix interference (Figure 8M), suggesting that the Meis1-circNfix-miR-214-Gsk3β regulatory axis is active in cardiomyocytes. Then, we investigated whether such a regulatory axis was involved in regulating cardiomyocyte proliferation. As expected, the miR-214 mimics significantly increased the percentage of proliferating cardiomyocytes (Figure XXXF in the online-only Data Supplement). Additionally, miR-214 inhibition counteracted the effect of circNfix downregulation on cardiomyocyte proliferation, which was reversed by Gsk3β knockdown (Figure 8N; Figure XXIXA through XXIXC in the online-only Data Supplement). Furthermore, the inhibitory effect of losing either Ybx1 or miR-214 is similar to the loss of both, abolishing circNfix-induced cardiomyocyte proliferation (Figure 8N; Figure XXIXA through XXIXC in the online-only Data Supplement). Our results demonstrated that circNfix requires both Ybx1 and miR-214 to regulate cardiomyocyte proliferation.

Considering the important role of Gsk3β on angiogenesis, we also detected VEGF (vascular endothelial growth factor) secretion via ELISA after altering this regulatory axis. Inhibiting miR-214 was found to abolish the facilitating effect of circNfix knockdown on VEGF secretion, which could be restored by Gsk3β downregulation (Figure XXXIA in the online-only Data Supplement). To clarify the link between circNfix, Gsk3β, and angiogenesis, we performed rescue experiments in adult and neonatal mice after MI. Coinjection of AAV9-Gsk3β and AAV9-shcircNfix substantially abolished the angiogenic effects of circNfix downregulation in adult mice after MI (Figure XXXIB through XXXID in the online-only Data Supplement). Moreover, the Gsk3β inhibitor SB216763 was used to perform rescue experiments in neonatal mice after MI as previously described.^27^ Interestingly, SB216763 treatments significantly restored the IB4^+^ capillary density in the peri-infarcted zone of circNfix-overexpressing mice (Figure XXXIE in the online-only Data Supplement). These results indicated Gsk3β as the main downstream effector mediating the function of circNfix in angiogenesis.

## Discussion

In the current study, we identified a cardiomyocyte-specific circRNA, circNfix, whose expression level is controlled by the interaction of its proximal superenhancer and Meis1. Downregulation of circNfix promotes cardiac regenerative repair and functional recovery after MI. Mechanistically, circNfix blocks cardiomyocyte proliferation by promoting Ybx1 ubiquitination degradation and acting as a miR-214 sponge.

In this study, we demonstrated that circRNAs are involved in cardiac regeneration, as superenhancer-associated circNfix negatively regulates cardiomyocyte proliferation both in vitro and in vivo by affecting the cardiomyocyte cell-cycle reentry and dedifferentiation processes. We further revealed that circNfix downregulation induced 2% to 4% cardiomyocyte proliferation in the peri-infarcted myocardium, which was sufficient to facilitate functional recovery after MI.^2,3,26,28^ Along with cardiomyocyte proliferation, circNfix downregulation promoted angiogenesis and inhibited cardiomyocyte apoptosis, which are also essential for cardiac repair and improvement of cardiac function after MI.^29^ These circNfix-induced beneficial effects decreased the fibrotic scar area by nearly 60% and restored cardiac function to almost normal levels after MI, indicating a potent therapeutic effect of attenuating postinfarction cardiac remodeling and subsequent heart failure. Recently, studies have indicated that ncRNAs, including miRNAs and lncRNAs (long noncoding RNAs), have important roles in regulating cardiomyocyte proliferation and cardiac regeneration.^3,28^ However, the use of these ncRNAs are limited by rapid degradation,^30^ poor conservatism^31^ and uncertain effects on humans.^32^ By contrast, we discovered that circNfix has structural robustness and a highly conservative nature. More importantly, circNfix expression in adult human myocardium was remarkably higher than that in fetal human myocardium. These characteristics suggest circNfix might overcome the stated ncRNA limitations and have clinical translational value. Notably, circNfix is clearly enriched in cardiomyocytes under basal conditions. Targeting circNfix might be less likely to have a significant negative effect on other organs and thus represents a promising strategy for improving prognosis after MI.

In this study, we proposed and confirmed a novel concept in which superenhancers could be used to identify key circRNAs during cardiac regeneration by marking the cell-identity subgroups. During the cardiac development process, the embryonic and adult stages of mouse hearts are quite different regarding cardiogenic potency.^33^ We compared their differences in the superenhancer catalog and found that most of their superenhancers are developmental specific, indicating the important role of superenhancers in cardiogenic potency. Similarly, we revealed that superenhancer-associated circRNAs have unique characteristics, including higher expression levels and developmental stage and tissue specificity, which are linked to regulators essential for cell differentiation and development. Based on these findings, we conducted an integrated analysis of superenhancer-associated circRNAs and their expression profiles in embryonic and adult hearts and further filtered out adult cardiomyocyte-specific circNfix. Functional assays confirmed that circNfix downregulation was required and sufficient for cardiac regeneration. Moreover, we observed that circNfix-associated superenhancers regulated circNfix expression by recruiting Meis1, a key transcription factor controlling cardiomyocyte cell-cycle arrest. Our results are similar to previous studies showing that superenhancers are involved in causal mechanisms underlying the induction of cell differentiation and proliferation by recruiting master transcription factors.^13^ Collectively, superenhancer-associated circRNAs might be the key regulators of cardiac regeneration because of their specific expression profiles and causal functions in inducing cell differentiation and proliferation. The identification of ncRNAs with these features from traditional genome-wide profile analyses is time and labor intensive, and, more importantly, susceptible to false-positive discovery.^34^ By contrast, integrated analysis of superenhancers and their associated circRNA expression profiles might serve as a powerful filter to quickly identify the key regulators in heart regeneration processes according to RNA expression and genetic alteration.

In this study, we investigated the mechanisms through which circNfix blocks cardiomyocyte proliferation and found that they are related to Ybx1. circRNAs often interact with RNA-binding proteins to fulfill their biological functions. The RNA pulldown, radioimmune precipitation, and fluorescense in situ hybridization assays confirmed that circNfix interacts with Ybx1. As an important transcription factor associated with cell proliferation, Ybx1 is involved in embryogenesis,^35^ muscle regeneration,^36^ oncogenic cell transformation,^37^ and cardiomyocyte differentiation/dedifferentiation.^38,39^ In the current study, Ybx1 induced cardiomyocyte proliferation by increasing cyclin A and cyclin B1 transcription, suggesting Ybx1 as an essential regulator of cardiac regeneration. Moreover, our studies showed that the interaction between circNfix and Ybx1 prevented nuclear translocation of Ybx1, causing cytoplasmic retention and degradation. The E3 ubiquitin ligases have been demonstrated to induce polyubiquitination and proteasomal degradation of Ybx1.^40^ Nedd4l is a member of the E3 ubiquitin ligases that are required for heart development.^41^ Our study revealed that Ybx1 is a substrate for E3 ubiquitin ligase Nedd4l in the presence of circNfix. The formation of the circNfix/Ybx1/Nedd4l ternary complex arrests Ybx1 in the cytoplasm and promotes Ybx1 degradation through ubiquitination-proteasome pathways. Although the exact molecular mechanism for the associations among circNfix, Ybx1, and Nedd4l remains to be clarified, we speculate that the interaction of Ybx1 with circNfix might change the conformation of Ybx1 and thus exposes its concealed ubiquitination sites to Nedd4l.

To understand the critical role of circNfix in cardiac regeneration, we further explored another mechanism by which circNfix exerts its functions. Bioinformatic tools predicted that circNfix contains 3 binding sites for miR-214, which has been demonstrated to be essential for cardiomyocyte proliferation.^3^ In the current study, we found that miR-214 was involved in circNfix knockdown, promoting cardiomyocyte proliferation by inhibiting Gsk3β expression. Moreover, our study revealed that the circNfix/miR-214/Gsk3β pathway regulates VEGF release from cardiomyocytes. We also observed that circNfix downregulation significantly promoted angiogenesis after MI. Previous studies have indicated an association between miR-214 and Gsk3β and their important roles in endothelial cell angiogenesis.^42–44^ Thus, the circNfix/miR-214/Gsk3β pathway might play an important role in regulating angiogenesis after MI. Compared with other ceRNAs (competing endogenous RNAs) shown to sponge miR-214, circNfix might be more efficient in modulating miR-214 activity attributable to its abundance, stability, and the numbers of miR-214 binding sites. These mechanistic studies further confirmed that circNfix is a crucial regulator of cardiac regeneration by regulating cardiomyocyte proliferation and angiogenesis.

Our study has certain limitations. First, although the effect of circNfix on Ybx1 and miR-214 functions are rather evident, more detailed experiments in vivo might help to further verify their roles. Second, we used CRISPR-Cas9 knock-in mice to generate Meis1-deficient and circNfix-deficient mice. Although Meis1 and circNfix were not completely removed, our results from these deficient mice are quite reliable to determine the function of Meis1 and circNfix. Confirming our findings in complementary conventional genetic models would be interesting.

## Conclusions

The cardiac-specific circRNA Nfix, which is regulated by an superenhancer, inhibits cardiomyocyte proliferation by promoting Ybx1 ubiquitin-dependent degradation and repressing miR-214 activity. Loss of circNfix induced cardiac regeneration and angiogenesis and inhibited cardiomyocyte apoptosis after MI, which significantly restored cardiac function and improved the prognosis. Therefore, the newly discovered circNfix might be a potent therapeutic target for restoring cardiac function and preventing heart failure after MI.

## Sources of Funding

This work was supported by grants to Dr Bin from the National Natural Science Foundation of China (No. 81771857, No. 81571698, and No. 81271640).

## Disclosures

None.

## Supplementary Material

## References

[1] Karra R, Poss KD (2017). Redirecting cardiac growth mechanisms for therapeutic regeneration.. J Clin Invest.

[2] Chen Y, Li X, Li B, Wang H, Li M, Huang S, Sun Y, Chen G, Si X, Huang C, Liao W, Liao Y, Bin J (2019). Long non-coding RNA ECRAR triggers post-natal myocardial regeneration by activating ERK1/2 signaling.. Mol Ther.

[3] Li X, He X, Wang H, Li M, Huang S, Chen G, Jing Y, Wang S, Chen Y, Liao W, Liao Y, Bin J (2018). Loss of AZIN2 splice variant facilitates endogenous cardiac regeneration.. Cardiovasc Res.

[4] Qu S, Yang X, Li X, Wang J, Gao Y, Shang R, Sun W, Dou K, Li H (2015). Circular RNA: a new star of noncoding RNAs.. Cancer Lett.

[5] Hansen TB, Jensen TI, Clausen BH, Bramsen JB, Finsen B, Damgaard CK, Kjems J (2013). Natural RNA circles function as efficient microRNA sponges.. Nature.

[6] Du WW, Yang W, Liu E, Yang Z, Dhaliwal P, Yang BB (2016). Foxo3 circular RNA retards cell cycle progression via forming ternary complexes with p21 and CDK2.. Nucleic Acids Res.

[7] Li M, Ding W, Sun T, Tariq MA, Xu T, Li P, Wang J (2018). Biogenesis of circular RNAs and their roles in cardiovascular development and pathology.. FEBS J.

[8] Li L, Guo J, Chen Y, Chang C, Xu C (2017). Comprehensive circRNA expression profile and selection of key circRNAs during priming phase of rat liver regeneration.. BMC Genomics.

[9] Wang K, Long B, Liu F, Wang JX, Liu CY, Zhao B, Zhou LY, Sun T, Wang M, Yu T, Gong Y, Liu J, Dong YH, Li N, Li PF (2016). A circular RNA protects the heart from pathological hypertrophy and heart failure by targeting miR-223.. Eur Heart J.

[10] Zeng Y, Du WW, Wu Y, Yang Z, Awan FM, Li X, Yang W, Zhang C, Yang Q, Yee A, Chen Y, Yang F, Sun H, Huang R, Yee AJ, Li RK, Wu Z, Backx PH, Yang BB (2017). A circular RNA binds to and activates AKT phosphorylation and nuclear localization reducing apoptosis and enhancing cardiac repair.. Theranostics.

[11] Du WW, Yang W, Chen Y, Wu ZK, Foster FS, Yang Z, Li X, Yang BB (2017). Foxo3 circular RNA promotes cardiac senescence by modulating multiple factors associated with stress and senescence responses.. Eur Heart J.

[12] Werfel S, Nothjunge S, Schwarzmayr T, Strom TM, Meitinger T, Engelhardt S (2016). Characterization of circular RNAs in human, mouse and rat hearts.. J Mol Cell Cardiol.

[13] Whyte WA, Orlando DA, Hnisz D, Abraham BJ, Lin CY, Kagey MH, Rahl PB, Lee TI, Young RA (2013). Master transcription factors and mediator establish super-enhancers at key cell identity genes.. Cell.

[14] Hnisz D, Abraham BJ, Lee TI, Lau A, Saint-André V, Sigova AA, Hoke HA, Young RA (2013). Super-enhancers in the control of cell identity and disease.. Cell.

[15] Suzuki HI, Young RA, Sharp PA (2017). Super-enhancer-mediated RNA processing revealed by integrative microRNA network analysis.. Cell.

[16] Micheletti R, Plaisance I, Abraham BJ, Sarre A, Ting CC, Alexanian M, Maric D, Maison D, Nemir M, Young RA (2017). The long noncoding RNA *Wisper* controls cardiac fibrosis and remodeling.. Sci Transl Med.

[17] Adam RC, Yang H, Rockowitz S, Larsen SB, Nikolova M, Oristian DS, Polak L, Kadaja M, Asare A, Zheng D, Fuchs E (2015). Pioneer factors govern super-enhancer dynamics in stem cell plasticity and lineage choice.. Nature.

[18] Ounzain S, Micheletti R, Arnan C, Plaisance I, Cecchi D, Schroen B, Reverter F, Alexanian M, Gonzales C, Ng SY, Bussotti G, Pezzuto I, Notredame C, Heymans S, Guigó R, Johnson R, Pedrazzini T (2015). CARMEN, a human super enhancer-associated long noncoding RNA controlling cardiac specification, differentiation and homeostasis.. J Mol Cell Cardiol.

[19] Anderson KM, Anderson DM, McAnally JR, Shelton JM, Bassel-Duby R, Olson EN (2016). Transcription of the non-coding RNA upperhand controls Hand2 expression and heart development.. Nature.

[20] Li B, Hu Y, Li X, Jin G, Chen X, Chen G, Chen Y, Huang S, Liao W, Liao Y (2018). Sirt1 antisense long noncoding RNA promotes cardiomyocyte proliferation by enhancing the stability of Sirt1.. J Am Heart Assoc.

[21] Huang C, Huang S, Li H, Li X, Li B, Zhong L, Wang J, Zou M, He X, Zheng H, Si X, Liao W, Liao Y, Yang L, Bin J (2018). The effects of ultrasound exposure on P-glycoprotein-mediated multidrug resistance in vitro and in vivo.. J Exp Clin Cancer Res.

[22] Ahmed I, Karedath T, Andrews SS, Al-Azwani IK, Mohamoud YA, Querleu D, Rafii A, Malek JA (2016). Altered expression pattern of circular RNAs in primary and metastatic sites of epithelial ovarian carcinoma.. Oncotarget.

[23] Piwecka M, Glažar P, Hernandez-Miranda LR, Memczak S, Wolf SA, Rybak-Wolf A, Filipchyk A, Klironomos F, Cerda Jara CA, Fenske P (2017). Loss of a mammalian circular RNA locus causes miRNA deregulation and affects brain function.. Science.

[24] Platt RJ, Chen S, Zhou Y, Yim MJ, Swiech L, Kempton HR, Dahlman JE, Parnas O, Eisenhaure TM, Jovanovic M, Graham DB, Jhunjhunwala S, Heidenreich M, Xavier RJ, Langer R, Anderson DG, Hacohen N, Regev A, Feng G, Sharp PA, Zhang F (2014). CRISPR-Cas9 knockin mice for genome editing and cancer modeling.. Cell.

[25] Jurchott K, Bergmann S, Stein U, Walther W, Janz M, Manni I, Piaggio G, Fietze E, Dietel M, Royer HD (2003). YB-1 as a cell cycle-regulated transcription factor facilitating cyclin A and cyclin B1 gene expression.. J Biol Chem.

[26] D Uva G, Aharonov A, Lauriola M, Kain D, Yahalomronen Y, Carvalho S, Weisinger K, Bassat E, Rajchman D, Yifa O (2015). ERBB2 triggers mammalian heart regeneration by promoting cardiomyocyte dedifferentiation and proliferation.. Nat Cell Biol.

[27] Chen Z, Xie J, Hao H, Lin H, Wang L, Zhang Y, Chen L, Cao S, Huang X, Liao W, Bin J, Liao Y (2017). Ablation of periostin inhibits post-infarction myocardial regeneration in neonatal mice mediated by the phosphatidylinositol 3 kinase/glycogen synthase kinase 3β/cyclin D1 signalling pathway.. Cardiovasc Res.

[28] Eulalio A, Mano M, Dal Ferro M, Zentilin L, Sinagra G, Zacchigna S, Giacca M (2012). Functional screening identifies miRNAs inducing cardiac regeneration.. Nature.

[29] Li X, Sun Y, Huang S, Chen Y, Chen X, Li M, Si X, He X, Zheng H, Zhong L, Yang Y, Liao W, Liao Y, Chen G, Bin J (2019). Inhibition of AZIN2-sv induces neovascularization and improves prognosis after myocardial infarction by blocking ubiquitin-dependent talin1 degradation and activating the Akt pathway.. EBioMedicine.

[30] Clark MB, Johnston RL, Inostroza-Ponta M, Fox AH, Fortini E, Moscato P, Dinger ME, Mattick JS (2012). Genome-wide analysis of long noncoding RNA stability.. Genome Res.

[31] Carla AK, Johanna CS, Lauren ES, Robert KB, Paul AF, Matthew LS, Ding H, Vincent LB, Torrey L, Haas S (2013). Braveheart, a long noncoding RNA required for cardiovascular lineage commitment.. Cell.

[32] Romaine SP, Tomaszewski M, Condorelli G, Samani NJ (2015). MicroRNAs in cardiovascular disease: an introduction for clinicians.. Heart.

[33] GuimarãesCamboa Nuno, Stowe Jennifer, Aneas Ivy, Sakabe Noboru, Cattaneo Paola (2015). HIF1α represses cell stress pathways to allow proliferation of hypoxic fetal cardiomyocytes.. Dev Cell.

[34] Hu X, Feng Y, Zhang D, Zhao SD, Hu Z, Greshock J, Zhang Y, Yang L, Zhong X, Wang LP, Jean S, Li C, Huang Q, Katsaros D, Montone KT, Tanyi JL, Lu Y, Boyd J, Nathanson KL, Li H, Mills GB, Zhang L (2014). A functional genomic approach identifies FAL1 as an oncogenic long noncoding RNA that associates with BMI1 and represses p21 expression in cancer.. Cancer Cell.

[35] Lu ZH, Books JT, Ley TJ (2006). Cold shock domain family members YB-1 and MSY4 share essential functions during murine embryogenesis.. Mol Cell Biol.

[36] Fuke M, Narita M, Wada Y, Seto T, Okada K, Nakayama J, Izumi H, Ito KI (2018). Increased expression of Y-box-binding protein-1 in hind-limb muscles during regeneration from ischemic injury in mice.. Tohoku J Exp Med.

[37] Evdokimova V, Tognon C, Ng T, Ruzanov P, Melnyk N, Fink D, Sorokin A, Ovchinnikov LP, Davicioni E, Triche TJ, Sorensen PH (2009). Translational activation of snail1 and other developmentally regulated transcription factors by YB-1 promotes an epithelial-mesenchymal transition.. Cancer Cell.

[38] Wen J, Xia Q, Lu C, Yin L, Hu J, Gong Y, Yin B, Monzen K, Yuan J, Qiang B (2010). Proteomic analysis of cardiomyocytes differentiation in mouse embryonic carcinoma P19CL6 cells.. J Cell Biochem.

[39] David JJ, Subramanian SV, Zhang A, Willis WL, KR, Leier CV, Strauch AR (2012). Y-box binding protein-1 implicated in translational control of fetal myocardial gene expression after cardiac transplant.. Exp Biol Med.

[40] Chibi M, Meyer M, Skepu A, G Rees DJ, Moolman-Smook JC, Pugh DJ (2008). RBBP6 interacts with multifunctional protein YB-1 through its RING finger domain, leading to ubiquitination and proteosomal degradation of YB-1.. J Mol Biol.

[41] Gao S, Alarcón C, Sapkota G, Rahman S, Chen PY, Goerner N, Macias MJ, Erdjument-Bromage H, Tempst P, Massagué J (2009). Ubiquitin ligase Nedd4L targets activated Smad2/3 to limit TGF-beta signaling.. Mol Cell.

[42] Li HL, Liang S, Cui JH, Han GY (2018). Targeting of GSK-3β by miR-214 to facilitate gastric cancer cell proliferation and decrease of cell apoptosis.. Eur Rev Med Pharmacol Sci.

[43] Kaga S, Zhan L, Altaf E, Maulik N (2006). Glycogen synthase kinase-3beta/beta-catenin promotes angiogenic and anti-apoptotic signaling through the induction of VEGF, Bcl-2 and survivin expression in rat ischemic preconditioned myocardium.. J Mol Cell Cardiol.

[44] van Balkom BW, de Jong OG, Smits M, Brummelman J, den Ouden K, de Bree PM, van Eijndhoven MA, Pegtel DM, Stoorvogel W, Würdinger T, Verhaar MC (2013). Endothelial cells require miR-214 to secrete exosomes that suppress senescence and induce angiogenesis in human and mouse endothelial cells.. Blood.

