# Supplementary material for: Loss of Super-Enhancer-Regulated circRNA Nfix Induces Cardiac Regeneration After Myocardial Infarction in Adult Mice
Source: Circulation. 2019 Apr 5;139(25):2857–76. doi: 10.1161/CIRCULATIONAHA.118.038361 (PMC6629176; doi:10.1161/CIRCULATIONAHA.118.038361)
Supplement: Supplementary file 1 [file cir-139-2857-s001.pdf]

SUPPLEMENTAL MATERIAL

Supplemental Methods and Materials

Animal model establishment

C57BL/6 mice and SD rats were purchased from Guangdong Medical Laboratory Animal Center. The day of vaginal plug detection was designated as E0.5, and the day that newborns were observed was designated as P0. All animal experiments were approved by the Animal Research Committee of Southern Medical University and performed in accordance with the National Institutes of Health Guide for the Care and Use of Laboratory Animals.

Mouse MI was carried out as described[1]. Briefly, adult male mice (8-10 weeks of ages) were intraperitoneally anesthetized with 3% pentobarbital sodium (40 mg/kg). The left coronary artery was ligated with a silk suture 2 mm distal from the ascending aorta. An analogous surgical operation was performed without occlusion of the coronary artery to generate sham-operated animals. The chests were closed and the mice were warmed for several minutes until recovery. All mice were sacrificed by cervical dislocation at different time points.

Cardiac functions were measured by transthoracic echocardiography, using a Vevo 2100 high-resolution imaging system equipped with a 30-MHz transducer (RMV-707B, VisualSonics, Toronto, ON, Canada).

Cas9 knockin transgenic mouse model

The Cre-dependent Cas9 knockin mouse model was obtained from Shanghai Model Organisms Center, Inc. This model was generated by homologous recombination in JM8A3 embryonic stem (ES) cells and implanted in C57BL/6J blastocysts as standard procedures. Briefly, the targeting vector was designed to contain a ubiquitously expressed CAG promoter, a loxP-flanked PGK-Neo-polyA sequence followed by a Cas9 protein inserted into intron 1 of the Rosa26 locus. The construct was linearized and electroporated into JM8A3 embryonic stem cells. Correctly targeted ES cell colonies were screened by PCR, and PCR products were sequenced of essential regions to verify desired recombination. Correctly targeted ES cells were injected into blastocysts to obtain the chimeric mice. The resulting high-percentage chimeric male was crossed to female C57BL/6J mice to obtain the heterozygous of Cre-dependent Cas9 mice (R26-CAG-LSL-Cas9/+). R26-CAG-LSL-Cas9/+ mice were crossed with EIIa-Cre transgenic mice, generating R26-CAG-Cas9/+ mice. Homozygous of R26-CAG-Cas9 mice were obtained by intercrossing of R26-CAG-Cas9/+ mice and used in later experiments.

Human tissue sampling

Human left ventricular tissue samples were obtained as described previously[2, 3]. Briefly, two samples of adult myocardial tissues were obtained by endomyocardial biopsy from patients with suspected myocardial deposition disease, on the basis of arrhythmia and echocardiographic changes. The pathological findings did not show any evidence of myocardial disease or functional abnormalities. Fetal human

myocardial samples were obtained after the elective termination of pregnancy for nonmedical reasons. The present study conforms to the principles of the Declaration of Helsinki. The study protocol was approved by the Nanfang Hospital ethics committee, and written informed consent was obtained from all subjects.

Real-time polymerase chain reaction

Total RNA from cell or tissue lysates was isolated using TRIzol reagent (R6830-01E.Z.N.A, OMEGA). The nuclear and cytoplasmic fractions were extracted by using NE-PER Nuclear and Cytoplasmic Extraction Reagents (Thermo Scientific) following the manufacturer’s instructions. Reverse transcription was performed by using PrimeScript RT Master Mix (Takara, Dalian, China), and Q-PCR was performed by using SYBR Green PCR Master Mix (Takara, Dalian, China) in a LightCycler 480 System (Roche, Germany). We used stem-loop RT-PCRs to detect the amounts of miRNAs. The primers for RT-PCRs are listed in Supplemental Table 1.

Cell isolation and culture

Neonatal CMs were isolated from 1-day-old (P1), 7-day-old (P7) C57BL/6 mice or P1 SD rats as previously described[3-5]. Briefly, after anaesthesia with 2% isoflurane inhalation, the hearts of neonatal mice were isolated from the atria, splited and digested with 0.25% trysin (Sigma) at at 4 °C for 12h. Then, the hearts were digested again in type II collagenase (Roche), bovine serum albumin (BSA, Sigma) and PBS mixture at 37 °C for 15 min two to three times. The supernatant was collected and

mixed with fetal bovine serum (FBS) after each step. The supernatant was then centrifuged to collect the cells, which were resuspended in DMEM/F12 medium (Life Technologies) supplemented with 10% FBS. Then, the cell suspension was placed onto uncoated 100 mm plastic dishes for 80 min at 37 °C in 5% CO^2 and a humidified atmosphere due to different adhesion between cardiomyocyte and fibroblast. The supernatant, composed mostly of CMs, was then collected and pelleted. The cells were then resuspended in aforementioned medium, counted and plated at the appropriate density. Cultures of ventricular CMs that were prepared using this procedure consistently yielded a purity over 90%.

Adult CMs were isolated from 8 weeks old adult mouse as previously described[5], with minor modifications. Briefly, adult mouse hearts were extracted and mounted on a Langendoft apparatus. Then, hearts were perfused with calcium-free perfusion buffer containing 113 mM NaCl, 4.7 mM KCl, 0.6 mM KH_2PO_4, 0.6 mM Na_2HPO_4, 1.2 mM $MgSO_4$, 10 mM Na-HEPES, 12 mM $NaHCO_3$, 10 mM $KHCO_3$, 0.032 mM phenol red, 30 mM taurine, 10 mM BDM, and 5.5 mM glucose (pH-7.0) for 5 minutes, followed by 50 ml digestion buffer containing 15,000 U of type II collagenase (Roche) and 50 μM $CaCl_2$ for 10 mins. The hearts were teased into small pieces and triturated with a Pasteur pipette to separate individual CM. Cells were then spun at low speed and supernatant was removed. The adult mouse CMs were plated onto laminin (10 μg ml^{-1}, Life technologies, 23017015) coated culture slides with F12 medium with 10% FBS.

The primary neonatal cardiac endothelial cells and neonatal cardiac fibroblasts

were isolated from P7 mice using the Neonatal Cardiac Endothelial Cell Isolation kit (130-104-183, Miltenyi Biotec) and Neonatal Cardiac Fibroblast Cell Isolation kit (130-101-372, Miltenyi Biotec), respectively. The primary adult mouse cardiac endothelial cells and fibroblasts were obtained from Cell Biologics, Inc. (C57-6024 and C57-6049, respectively) and handled according to the company's instruction. Human primary coronary artery endothelial cells (HCAEC) were obtained from the American Type Culture Collection (ATCC) and cells culture was performed according to ATCC protocol. Hl-1 cardiac muscle cells were purchased from EMD Millipore (SCC065).

Small interfering RNAs (siRNAs), adenovirus (AdV) or adeno-associated virus 9 (AAV9) and vectors transfection

SiRNAs, vector harboring Ybx1, miR-214 mimics and inhibitors were purchased from Ribobio Co. Ltd. (Guangzhou, China). RNA interference target sequences are shown in Supplementary Table 2. A scrambled form was the control. Transfections were performed with the Lipofectamine 2000 kit (Invitrogen, Thermo Fisher Scientific) according to the manufacturer's instructions. SiRNAs, miR-214 mimic and inhibitor were transfected at a final concentration of 50 nM for 6 h. The subsequent experiments were performed 48h after transfection.

The ADV and AAV9-containing green fluorescent protein (GFP) vectors for depletion or overexpression of circNfix, the AAV9-containing mCherry for overexpression of Gsk3β, and the ADV-containing mCherry for overexpression Meis1

were synthesized as described previously[3-5]. The cTnT promotor was used to driven the gene and GFP or mCherry's expression. Neonatal mouse CMs were transfected with ADV for 48 h. The multiplicity of infection (MOI) was 10–20, and the CM transfection efficiency was >95%. Adult mice were intramyocardially injected with AAV9 vectors at 5–6 sites with a dose of 5×10^{11} viral genome particles per animal (approximately 20 μl) using an insulin syringe with a 30-gauge needle. For the infarcted mice, vectors were injected into the myocardium bordering the infarct zone. After injection for 14 days or 28 days, the heart tissues were collected. In the P0 neonatal mice, ADV, at a dose of 4×10^{9} viral genome particles per animal (approximately 5 μl), was injected into the heart ventricles at 3 sites by using an insulin syringe with a 30-gauge needle. The hearts of the injected mice were collected 4 or 7 days after ADV injection.

Ad-GFP and Ad-mCherry was used to determine the distribution of ADV or AAV9 vector in the myocardium. Bright field and fluorescence images of the adult mouse and isolated neonatal mouse heart were captured to examine GFP fluorescence using Bruker In-Vivo FX Pro system (Bruker, MA, USA). *In situ* hybridization (ISH) and real-time polymerase chain reaction (RT-qPCR) assays were used to detect circNfix expression after transfection.

Immunofluorescence analysis

Heart tissues were collected and embedded in OCT and frozen at −20 °C. Cultured cells were fixed in 4% polyformaldehyde (10 min). Then tissue sections and cultured

cells were permeabilized with 1% Triton in PBS for 10 min and incubated in 1% Bovine Serum Albumin (BSA) for 1h at room temperature. Then samples were incubated at 4 ℃ overnight with primary antibodies diluted in 1% BSA. The primary antibodies were used as followed: anti-Ki67 antibody (1:200, 275R, Cell Marque), anti- p-Histone H3 (pH3) antibody (ab170904, Abcam), anti-c-TnT (ab33589, Abcam), anti-aurora B (ab2254, Abcam), anti-α-SMA (ab7817, Abcam), anti-IB4 (ab181548, Abcam), anti-Ybx1 (ab76149, Abcam), anti-PCM1 (19856-1-AP, ProteinTech), anti-CD31 (AF3628, R&D Systems), anti-GFP (ab13970, Abcam) and mCherry (ab183628, Abcam). To detect EdU incorporation, cells were stained using a Click-it EdU Imaging Kit (Life Technologies, #C10638) according to the manufacturer's instruction. To detect apoptotic cells, samples were processed using an *In Situ* Cell Death Detection Kit (Roche, Shanghai, China). After washing with PBS, samples were stained with fluorescent secondary antibodies (Alexa Fluor 647, Abcam) for 2h at room temperature followed by 30 min of DAPI (ab104139, Abcam). Unless mentioned otherwise, image acquisition was performed using Carl Zeiss confocal microscopy. Vessel density was calculated by dividing the IB4/CD31/α-SMA positive stained areas by the total tissue area in the corresponding slight field using Image-J software (Wayne Rasband), similar to previously described[6, 7].

Histological examinations

Heart tissues were fixed with 10% formalin overnight, then embedded in wax and sectioned. The sections were deparaffinized through a graded alcohol series. Sections

were boiled in a pressure cooker and then cooled for 30 minutes. Then, specimens were processed as indicated.

To evaluate the capillary density, sections were blocked with 4% goat serum and incubated with anti-CD31 (ab7388, Abcam) and anti-vWF (ab11713, Abcam). The number of capillaries per unit area image from 200X fields was determined using an Olympus BX51 microscope (Olympus Corporation).

To measure the fibrotic area, the sections were stained with Masson trichrome (MST 8004, MST Biotechnology). The percent area of cardiac fibrosis was determined by detecting collagen deposition (blue) using Image J software.

The expression levels of circNfix in humans, rats and mouse heart tissues were detected by ISH. Heart sections were incubated in 3% pepsin dilution in fresh citrate buffer at 37°C for 30 min and then prehybridized with prehybridization solution for 2 h at 37°C. Subsequently, hybridization with DIG-labeled RNA probes (Biosense Bioscience Co., Ltd., Guangzhou, China) was performed overnight at 37°C. Sections were washed through a graded SSC buffer series. After blocking with 3% BSA for 30 min at 37°C, the sections were incubated with alkaline phosphatase-conjugated sheep anti-DIG Fab fragments for 1 h at room temperature. BM Purple AP substrate (Roche, Basel, Switzerland) was used to detect positive staining according to the manufacturer’s instructions.

SgRNA design

CRISPRtool programme was used to design sgRNAs to reduce potential off-target

effects. The sequence of sgRNA are listed in Supplemental Table 3.

Chromosome conformation capture (3C) assay

Samples were prepared as described previously with minor modifications[8]. Briefly, cells (5×10^6) were incubated into 1% formaldehyde and 2.5M glycine. Then, cells were spun and re-suspended in HiC lysis buffer (10mM Tris-HCl pH8.0, 10mM NaCl, 0.2% NP-40) with proteinase inhibitor (Sigma). Next, the cells were incubated into 0.5% sodium dodecyl sulfate (SDS) at 65°C for 5min. Chromatin was digested overnight by MboI and then ligated. Ligated DNA was quantified using QRT-PCR as described above. The anchor primer located at the circNfix promoter. Primer sequences are shown in Supplementary Table 4.

RNase R treatment

RNase R treatment was performed as described previously[9]. Briefly, 2 μg of total RNA was incubated with or without 3 U ug-1 of RNase R (Sigma) at 37°C for 30 min. Then, the treated RNA was purified using an RNeasy MinElute Cleanup Kit (Qiagen).

Western blot analysis

Total cells or tissue extracts were prepared by using RIPA lysis buffer (BestBio) containing protease inhibitor cocktail Set I (BestBio). Protein was fractionated in 10% SDS-PAGE gels and transferred to nitrocellulose membranes. The following primary antibodies were used: Ybx-1 mAb (20339-1-AP, Proteintech), Gsk3β (ab32391,

Abcam), Meis1 (ab19867, Abcam), Nedd4l (13690-1-AP, Proteintech), β-catenin (#8480, CST), Cyclin A2 (ab181591) and Cyclin B1 (55004-1-AP, Proteintech). As a control, we used the mouse anti-β-actin antibody (1:1000 dilution; Santa Cruz, CA, USA). The Alexa Fluor 680 conjugated anti-mouse IgG (1:10000 dilution; Abcam, USA) was used as the secondary antibody. The signal was detected by an Odyssey detection system (LI-COR Biosciences, Lincoln, NE, USA).

RNA Fluorescent in situ hybridization (RNA-FISH)

Cy3-labeled RNA probes against circNfix back-splice sequence, miR-214, or Gsk3β 3'UTR sequence were used in RNA-FISH assays. Cultured cells were washed with PBS and permeabilized with 0.5% Triton X-100. Then, cells were incubated with RNA probes in the hybridization buffer (RiboBio, Guangzhou, China). Nuclei were stained with DAPI. All probes used for RNA-FISH were provided by RiboBio Co., Ltd. (Guangzhou, China).

RNA pulldown assays

Cultured cells were washed in ice-cold phosphate-buffered saline, lysed in 0.5 ml co-IP buffer, and incubated with 3 μg biotinylated DNA oligo probes against circNfix back-splice sequence at room temperature for 4 h. Then, the cells were incubated in 50 μl washed streptavidin-coated magnetic beads (SA1004; Invitrogen) at room temperature for another hour. RNase-free BSA and yeast tRNA (Sigma, Shanghai, China) were used to prevent the nonspecific binding of RNA and protein complexes.

RNA bound to beads was extracted by TRIzol, while the bound protein was analyzed by western blotting. The specific bands were extracted and then analyzed by mass spectrometry. The results of mass spectrometry are present in Supplemental Table 5 and Table 6.

RNA immunoprecipitation (RIP)

RIP assays were performed using a Magna RIPTM RNA-binding Protein Immunoprecipitation Kit (Millipore, Stafford, VA) according to the manufacturer's instructions. The Ybx1 antibody (20339-1-AP, Proteintech) and Nedd4l antibody (13690-1-AP, Proteintech) were used to precipitate RNA. A qRT-PCR analysis was then used to demonstrate the presence of binding.

Triphenyltetrazolium chloride (TTC) staining

The TTC assays were performed as previously described in detail[10]. Briefly, the mouse hearts were harvested and sectioned using metal slicers into 3 mm thick slices. The slices were incubated in 1% TTC (Sigma Aldrich) dissolved in PBS for 15 min at room temperature. The slices were washed with PBS to stop the staining process and then photographed. Image-Pro Plus 6.0 (Media Cybernetics, Bethesda, MD, USA) was used to determine the infarcted area.

Luciferase reporter assay

The circNfix sequence, the 3' UTR of Gsk3β and SE constituent enhancers were

cloned into the luciferase vector psiCHECK-2 (Saicheng Bio Co., Ltd., Guangzhou, China). All constructs were verified by sequencing. For luciferase reporter assays, the miR-214 mimic was cotransfected into CMs with the luciferase reporters described above. Transfection was performed using Lipofectamine 2000 (Invitrogen, Thermo Fisher Scientific). ADV-circNfix transfection was performed for 48 h, while siRNA-Meis1 transfection was performed 24 h before luciferase reporter transfection. Luciferase activity was detected by the Dual Luciferase Reporter Assay System (Promega, Madison, WI).

Chromatin immunoprecipitation (ChIP) assay

ChIP assays were performed as described previously[11]. An EpiQuik Chromatin Immunoprecipitation Assay Kit (EpiGentek, Brooklyn, NY) was used according to the manufacturer's instructions. Ten micrograms of anti-Ybx1 antibody or control IgG antibody was used for immunoprecipitation. Next, qRT-PCR and PCR gel electrophoresis were applied to detect the enrichment of DNA fragments in the predicted Ybx1 binding sites. The primers used are listed in Supplementary Table 1.

Electrophoretic mobility shift assays (EMSA)

EMSA was performed using EMSA Kit (Biosense, Guangzhou) according to the manufacturer’s instructions. The used probes are shown in Supplementary Table 2. One hundred-fold unlabeled wide-type Meis1 probes was added to the binding mixture 10 min before the addition of the labelled probe for competitions experiments.

BioSens Gel Imaging System (BIOTOP, China) was used to detected bands. The sequence of the wide-type probe is 5'-TGACAGGCTGTCACCGCGCGAC-3', and the mutated probe is 5'- TGACGCATGACTCATACGCGAC-3'.

Stereological Analysis

Left ventricle (including the septum) were sampled as previously described[12]. Briefly, heart tissues were embedded in 8% gelatin, and isectors were used to obtain a maximum diameter of 4 mm, isotropic, uniform random alignment of the samples. These isectors were used for stereological analysis. Anti-PCM1 antibody was applied to label CMs nuclei. Wheat germ agglutinin (WGA) was added to identify the cell borders. A minimum of 3–4 isectors were stained, and a minimum of 200 nuclei per animal were counted (nearly 2% of the area of the region of interest). CMs were cut along their longitudinal axis to determine the number of nuclei per cells. The two-step $N_V X\ V_{REF}$ method was utilized to estimate the total numbers of nuclei in the heart, as previously described. N_V was an estimate of the numerical CM density and V_{REF} is the reference left ventricle volume. The total number of CMs was calculated based on the number of CM nuclei and the multinucleation level. The analysis was performed on the confocal laser scanning microscopy (Carl Zeiss).

Volume Analysis of Isolated Cardiomyocytes

The volume of the isolate cardiomyocytes was investigated as previously described with modifications[12]. Z-stack images of CMs were obtained using a Zeiss confocal

LSM 700 microscope, and the individual CM volume was measured by the Imaris 8 (Bitplane) 3D image processing software program.

Flow cytometry

Flow cytometry assays were performed as described previously. Briefly, after transfected with siRNAs, isolated CMs were cultured for 48h and then collected and fixed with cold 70% ethanol. Samples were centrifuged for 15 min at ×1200 g. Cell pellets were re-suspended in FxCycle™ PI/RNase Staining Solution (Thermo Fisher Scientific) and analyzed on MoFlo XDP (Cell Sorter).

Time-lapse videos

Primary P7 CMs were isolated and cultured in confocal dishes with DMEM/F12 medium (Thermo Fisher Scientific) supplemented with 10% FBS. Twenty-four hours after seeding CMs were labelled using TMRE (tetramethylrhodamine, Thermo Fisher Scientific) as a fluorescent dye. Time-lapse videos were acquired for 24 h at 10 min intervals, and acquired at ×40 magnification using Leica (TCS Sp8) confocal microscope. Immunostaining with anti-cTnT antibody was used to determine whether the cells undergoing cell division were CMs.

Positron emission tomography–computed tomography (PET-CT)

PET-CT was performed using NanoScan® PET/CT systems. 18F-FDG (15 MBq) dilated into 100 mL 0.9% saline was injected intravenously 1 h before each scan.

List-mode data were detected for 15 min and subsequently reconstructed into a single image volume of 110X60X60 mm^3, voxel size of 0.4X0.4X0.4 mm^3. The myocardial perfusion score was calculated as previously described[13].

Data availability

CircRNA expression from human tissue samples were obtained from previous publications[9]. CircRNAs expression profile in adult and neonatal rat hearts, and conserved sequence identification from datasets was also previously described[14]. The threshold for up- and down-regulated circRNAs was a fold change of ≥1.5 and FDR-adjusted *P*-value<0.05. Gene expression profile in adult and neonatal (P3) mouse heart were downloaded from GSE51483.

The locations of human SEs and mouse SEs were acquired from previous publications[15, 16]. In agreement with previous studies[15], we used proximity to assign enhancers to the expressed transcript which is nearest to the center of enhancer. The data of ChIP-seq is available through the Gene Expression Omnibus (GEO) using the following accession numbers: adult CMs H3K27ac (GSM1902461), fetal CMs H3K27ac (GSM2108809), Meis1 (GSM819083), E14.5 mouse heart H3K27ac (GSM1264374), P0 mouse heart H3K27ac (GSM1264378), P7 mouse heart H3K27ac (GSM1264380), P56 mouse heart H3K27ac (GSM1264384), fetal human heart H3K27me3 (GSM1598027), adult mouse heart H3K4me1 (GSM769025), fetal mouse heart H3K4me1 (GSE86753), adult mouse heart H3K27me3 (GSM1000131), fetal mouse heart H3K27me3 (GSE86693), adult mouse heart P300 (GSM2346458), fetal

mouse heart P300 (GSM2346444), adult mouse heart DNA hypersensitivity (DHS, GSM1014166), fetal mouse heart DHS (GSM530661), adult human heart H3K27ac (GSE101345), adult human heart H3K4me1 (GSE101156), adult human heart H3K27me3 (GSE101387), fetal human heart H3K27ac (GSM1598027), fetal human heart H3K4me1 (GSM706848), fetal human heart H3K27me3 (GSM621450), The data of multiple tissues H3K27ac are available through ENCODE database. ChIP data was analyzed as previously described[15]. PhastCons scores by chromosome were downloaded from the University of California, Santa Cruz (UCSC) genome browser (https://genome.ucsc.edu/).

Supplementary Table 1: The primers used for qPCR in this study.

Primers name		Sequence (5'-3')
circNfix (divergent)	+	TCCAGCCACATCACATTG
	-	TGAACCAGGTGTAGGAGAA
circNfix (convergent)	+	TCTCCTACACCTGGTTCAA
	-	CAATGTGATGTGGCTGGA
mmu-circslc8a1	+	GTTGTTCAGACGGTATCTTC
	-	GTTGACTGTGAGCATCCA
Nfix mRNA	+	5'-GCATCGATCTCTCACCCTGG-3'
	-	5'- ATTGCTGCTGTGCAACAGTG-3'
Gsk3β	+	GAGACATCACGAGTTCATTG
	-	GGAAGAGAAGGTAACAGAGAT
Pten	+	GTAGATGGCTCAGTTGGAA
	-	AGACAGATGGACAGATGGA
α-catenin 1	+	GGTCTGGAGAAGGAAGGT
	-	TGGTGATTGCTGTAGTAGTC
α-catenin 2	+	AAGCAGTGACTCCTCCAT
	-	GATAGACCAGCAGCATTGA
α-catenin 3	+	GGATGACACAAGCAATGAC
	-	TCTGGACACTGGTTAGCA
Bcl-2	+	CCTCCAATACTCACTCTGTC
	-	TACCTGCGTTCTCCTCTC
Cyclin A2	+	CAGGAAGACCAAGAGAATGT
	-	ATGAAGGACCAGCAGTGA
Cyclin B1	+	CAGCACTACCTATCCTACAG
	-	CTCAGAAGCAACAACATTCA
MiR-214	+	ACGCGACAGCAGGCACAGAC
	-	ATCCAGTGCAGGGTCCGAGG
	R	GTCGTATCCAGTGCAGGGTCCGAGGTATTC GCACTGGATACGACCTGCCT
MiR-761	+	AGC CGG CAG CAG GGT GAA ACT
	-	ATCCAGTGCAGGGTCCGAGG
	R	GTCGTATCCAGTGCAGGGTCCGAGGT ATTCGCACTGGATACGACTGTGTC
U6	+	GCGCGTCGTGAAGCGTTC
	-	GTGCAG GGTCCGAGGT
Gapdh	+	TGACCTCAACTACATGGTCTACA
	-	CTTCCCATTCTCGGCCTTG
RUNX1	+	TGGTGGAGGTACTAGCTGACC
	-	CGAGTAGTTTTCATCGTTGCCTG
α-SMA	+	CCCAGACATCAGGGAGTAATGG
	-	TCTATCGGATACTTCAGCGTCA

Nkx2.5	+	GACAAAGCCGAGACGGATGG
	-	CTGTCGCTTGCACTTGTAGC
DAB2	+	CCTTCATTGCTCGTGATGTGA
	-	CCCCAAACAAATCCATCTGGTC
ChIP-Cyclin A2	+	CTGATGTCACAACCAAGGA
	-	TAAAGGCGTGTCTCTATGC
ChIP-Cyclin B1	+	ACTTCATGCTATCAACCTCA
	-	CACTTGTGTGCTGTCCAA
ChIp-Meis1	+	CTTGGTGTGATCGCATGA
	-	ACTGCTCATCTCCACTCA

Supplementary Table 2: The RNA interference used in this study

siRNA name	Sequence (5’-3’)
ShcircNfix/si-circNfix	CACTCCGGGATGAGTTCCA
Si-Nfix	GTGACAGAGCTGGTGAGAGTA
Si-both	GCACGAGAAGCGGATGTCAAA
Si-Meis1	TTGAGGCTGACATTGGCATTCCAGG
Si-Gsk3β	GUCCUAGGAACACCAACAA
Si-Ybx1	CAACGUCGGUAUCGCCGAAACUUCA

Supplementary Table 3: The sequence of sgRNAs and primers for genomic PCR.

Oligo	Type	Sequence (5'-3')	Application
sgMeis1-1	sgRNA	ATGCGGGTCCCCATACATCG	In vitro screening
sgMeis1-2	sgRNA	TGCATGGACCTGGCTGCATG	In vitro screening
sgMeis1-3	sgRNA	TATGGGGGTATGGATGGAGT	AAV9 vector
sgcircNfix-upstream-1	sgRNA	GAGATTGTTCTACTGAAGTG	AAV9 vector
sgcircNfix-upstream-2	sgRNA	GGAGGGGGAAGACGGGATGA	In vitro screening
sgcircNfix-upstream-3	sgRNA	GCGGGGCGAGGGAGGACGGG	In vitro screening
sgcircNfix-downstream-1	sgRNA	ACTGGCCTCTGCTTCACACG	AAV9 vector
sgcircNfix-downstream-2	sgRNA	AGGCCAGTAGGACAGGCTAC	In vitro screening
sgcircNfix-downstream-3	sgRNA	TTCTGAACAGCACTGCTTCT	In vitro screening
sgMeis1 (F)	Primer	CCAAGTGAAGGCTGTGTGAAGC	
sgMeis1(R)	Primer	AAGCCGGGCGCTGAATCGG	
sgcircNfix-upstream (F)	Primer	GCATGTGTGTGTGTAAAGACGAGAG	
sgcircNfix-upstream (R)	Primer	AGTCAATCCGCCGGATCTTGC	
sgcircNfix-downstream (F)	Primer	GGCTGGACCTGGTCATGGTG	
sgcircNfix-downstream (R)	Primer	CATGGTGTGGTGTGTATCCATCC	

Supplementary Table 4: The primers used for 3C assays

Primers name	Sequence (5’-3’)
Anchor	AGCGAAGTCCAGCAGTTA
A	AGGAGAGGACTCGTTACAG
B	GGAGGACCACTTAGAAGGA
C	TTCCATTGCTACCTGTAGTT
D	TGATGGTGGCAGCCTATG
E	CTGTCCTACAACTAACTCACT
F	AGAGTGGAGTGACTGTGT
G	CGTCACCAGACACATAGC
H	CTGCGTGAACCTGGAAGT
I	TCCATCAGTGACCAGTATTC
J	GGTAACTTGCCAGAGTCAG
Control	TCAATCCTAGTGTTCCCAC

Supplemental Table 5: The results of mass spectrometry analysis of precipitant-1 related to Fig. 7A

Protein	-10lgP	Coverage (%)	Intensity	#Peptides	#Unique	#Spec	Avg. Mass
Ybx1	155.64	52	7.29E+07	9	9	9	35730
Actc1	250.71	58	1.54E+07	21	3	50	42019
Pccb	230.93	35	4.11E+08	12	12	19	54614
Atp5b	209.6	40	4.87E+08	17	17	29	56301
Eno1	182.27	30	1.35E+08	10	7	15	47141
Idh2	182.26	29	8.84E+07	12	10	15	50906
Uqcrc2	178.2	18	1.08E+08	7	7	8	48235
Cs	151.12	16	8.85E+07	6	6	7	51737
Got2	143.35	20	5.67E+07	7	7	9	47411
Vim	140.23	17	3.52E+07	7	5	7	49193
Hnrnpk	129.94	17	6.58E+07	6	6	6	50976
Eef1g	126.94	14	7.03E+07	6	6	6	50061
Cct3	122.63	10	1.54E+07	5	4	5	56527
Hnrnpk	129.94	18	6.58E+07	6	6	6	48562

Supplemental Table 6: The results of mass spectrometry analysis of precipitant-2 related to Fig. 7A.

Protein	-10lgP	Coverage (%)	Intensity	#Peptides	#Unique	#Spec	Avg. Mass
Nedd4l	54.11	1	1.78E+06	1	1	1	115419
Nedd4l	54.11	1	1.78E+06	1	1	1	112220
Pcx	164.81	12	7.82E+07	11	11	12	129701
Pc	164.81	12	7.82E+07	11	11	12	129684
Atp1a1	135.8	10	2.93E+07	8	5	8	112982
Vcl	128.32	8	2.06E+07	7	7	7	116717
Actn4	117.76	10	6.65E+06	7	3	8	107108
Actn4	117.76	10	6.65E+06	7	3	8	104977
Actn2	108.95	7	2.42E+07	5	5	5	103834
Hdlbp	108.8	5	2.35E+06	5	4	6	141742
Dsg1a	100.55	3	3.10E+07	4	4	4	114597
Dsg1b	100.55	3	3.10E+07	4	4	4	114454
Nnt	92.13	5	1.29E+07	5	5	5	113838
Mybpc3	82.76	2	1.09E+07	3	3	3	141395
Mybpc3	82.76	2	1.09E+07	3	3	3	141308
Mybpc3	82.76	2	1.09E+07	3	3	3	140632
Mybpc3	82.76	3	1.09E+07	3	3	3	124146
Parp1	81.26	5	5.14E+06	4	3	5	113100
Parp1	81.26	5	5.14E+06	4	3	5	112721
Col1a1	76.47	4	2.59E+07	4	4	4	138033
Col1a2	70.32	1	0.00E+00	1	1	1	129557
Col6a1	55	2	3.66E+06	2	2	2	108489
Col3a1	52.73	1	1.65E+06	1	1	1	138944

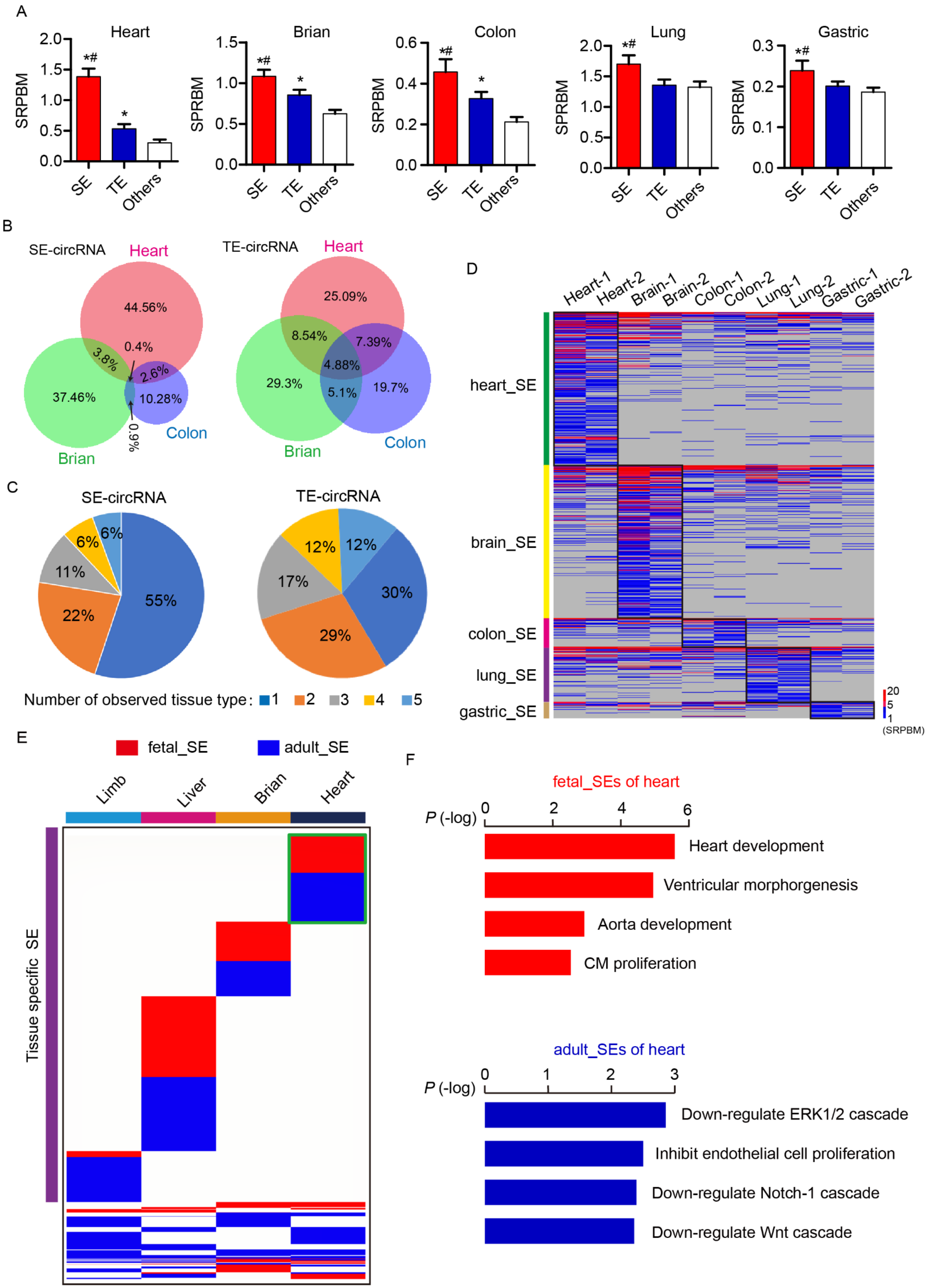

Supplemental Figure 1

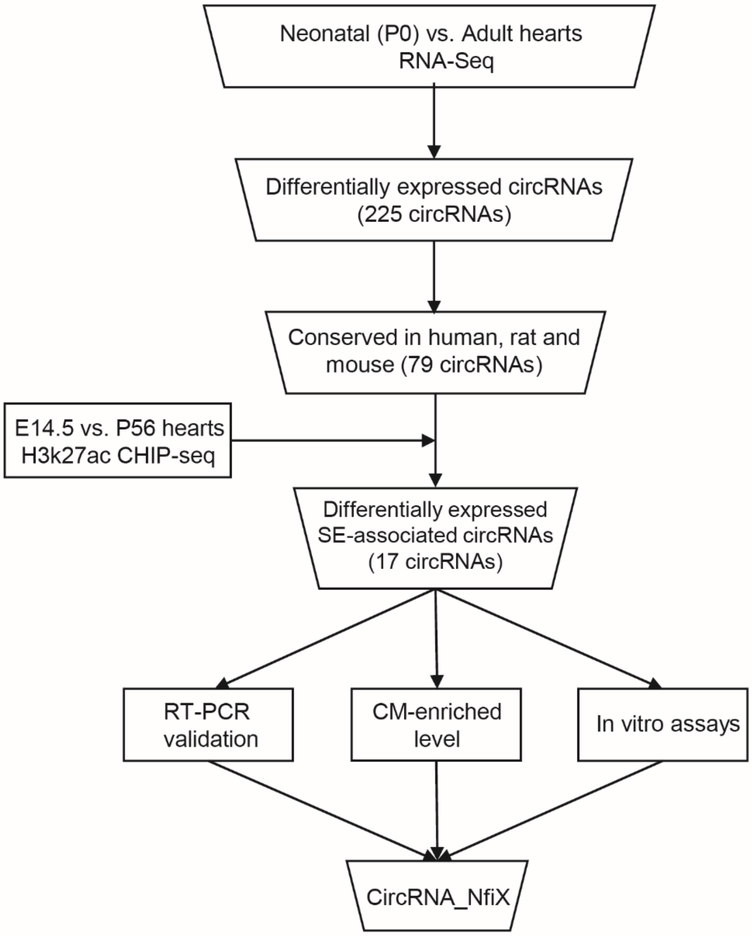

Supplemental Figure 2

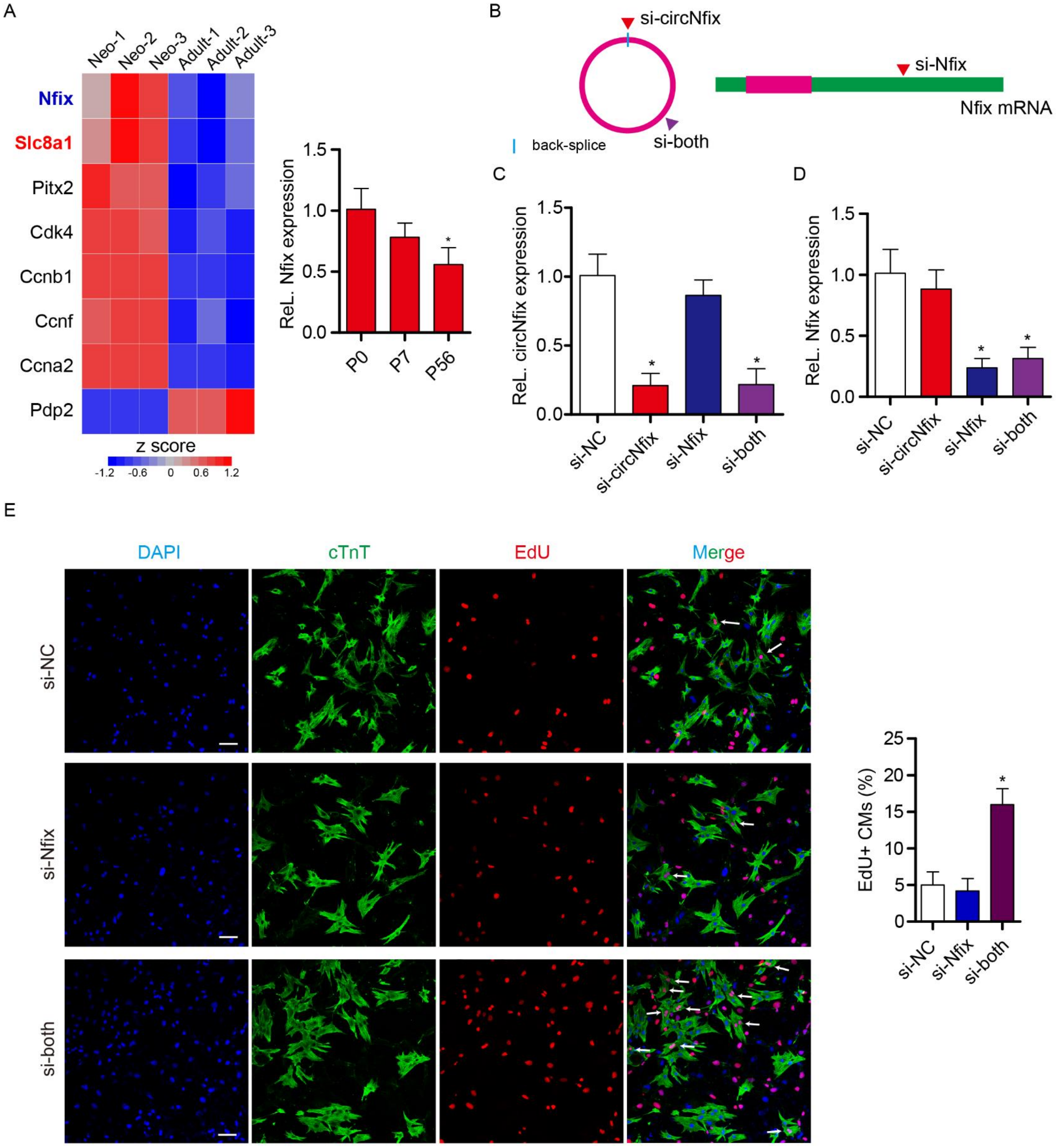

Supplemental Figure 3

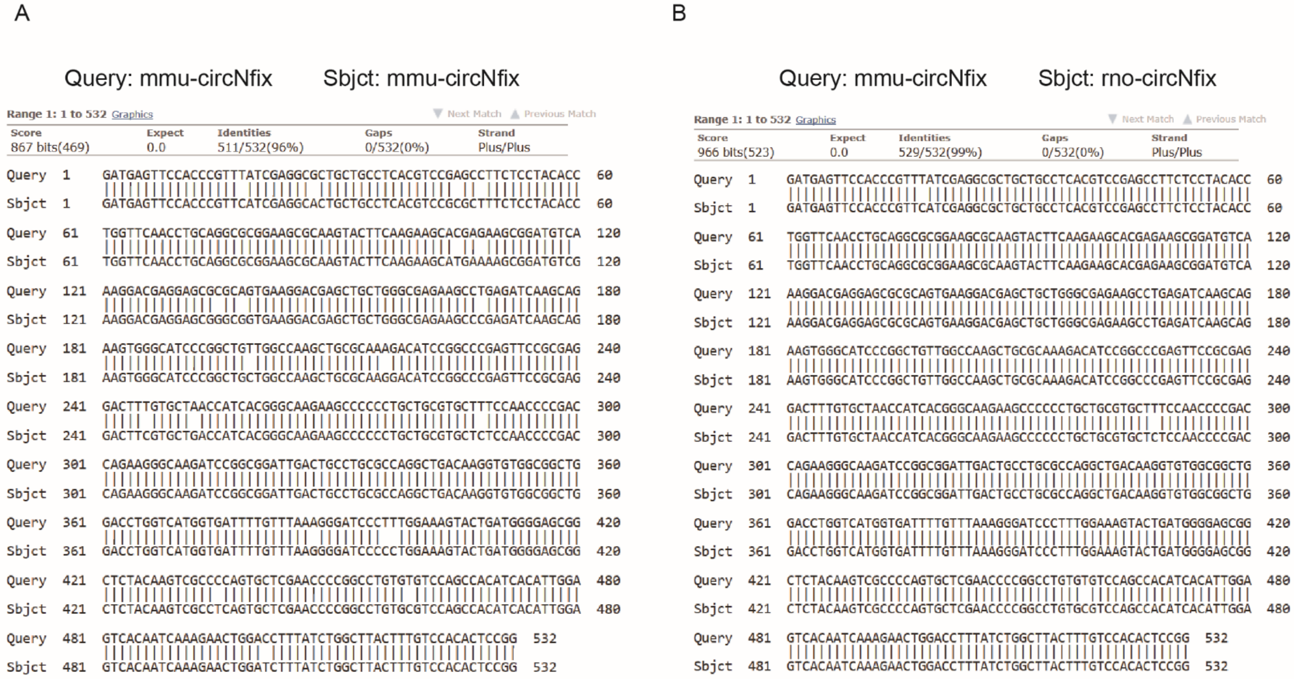

Supplemental Figure 4

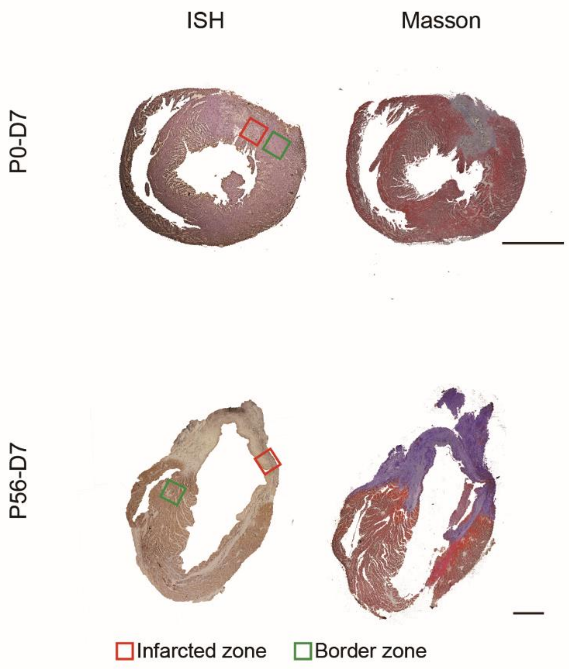

Supplemental Figure 5

A

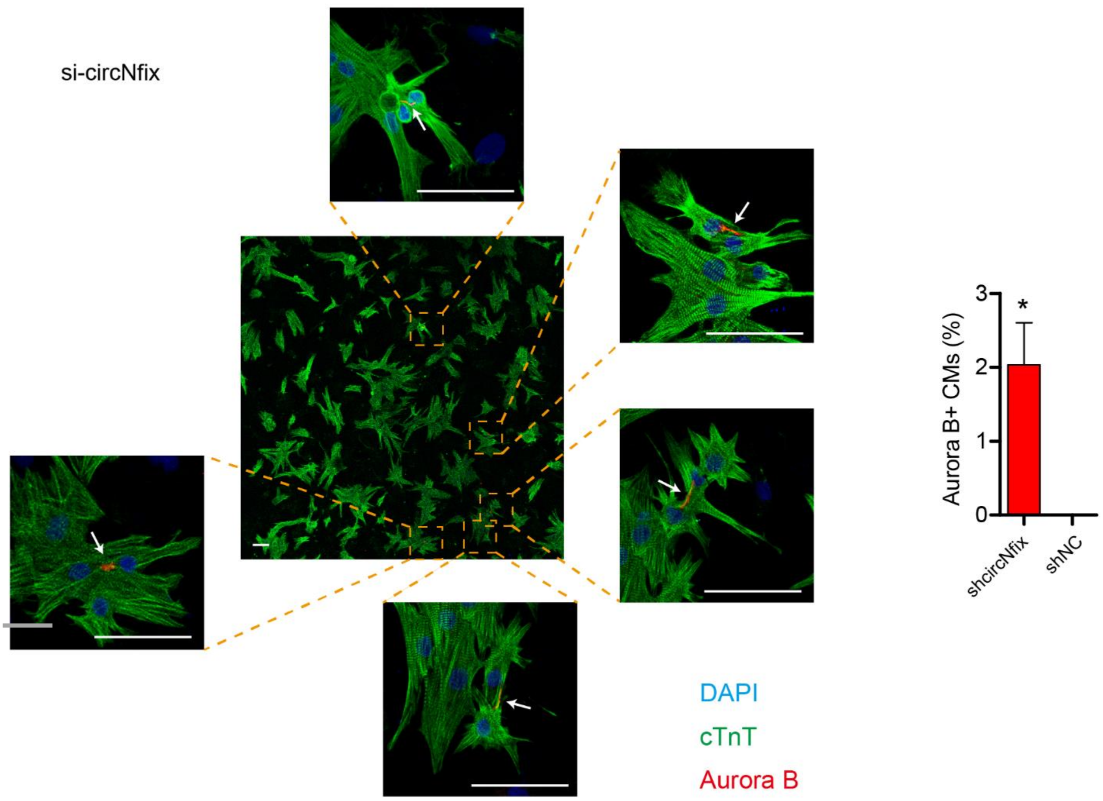

B

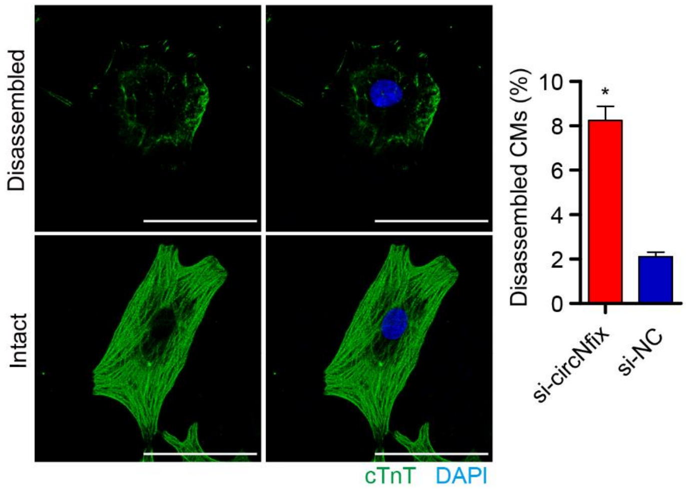

Supplemental Figure 6

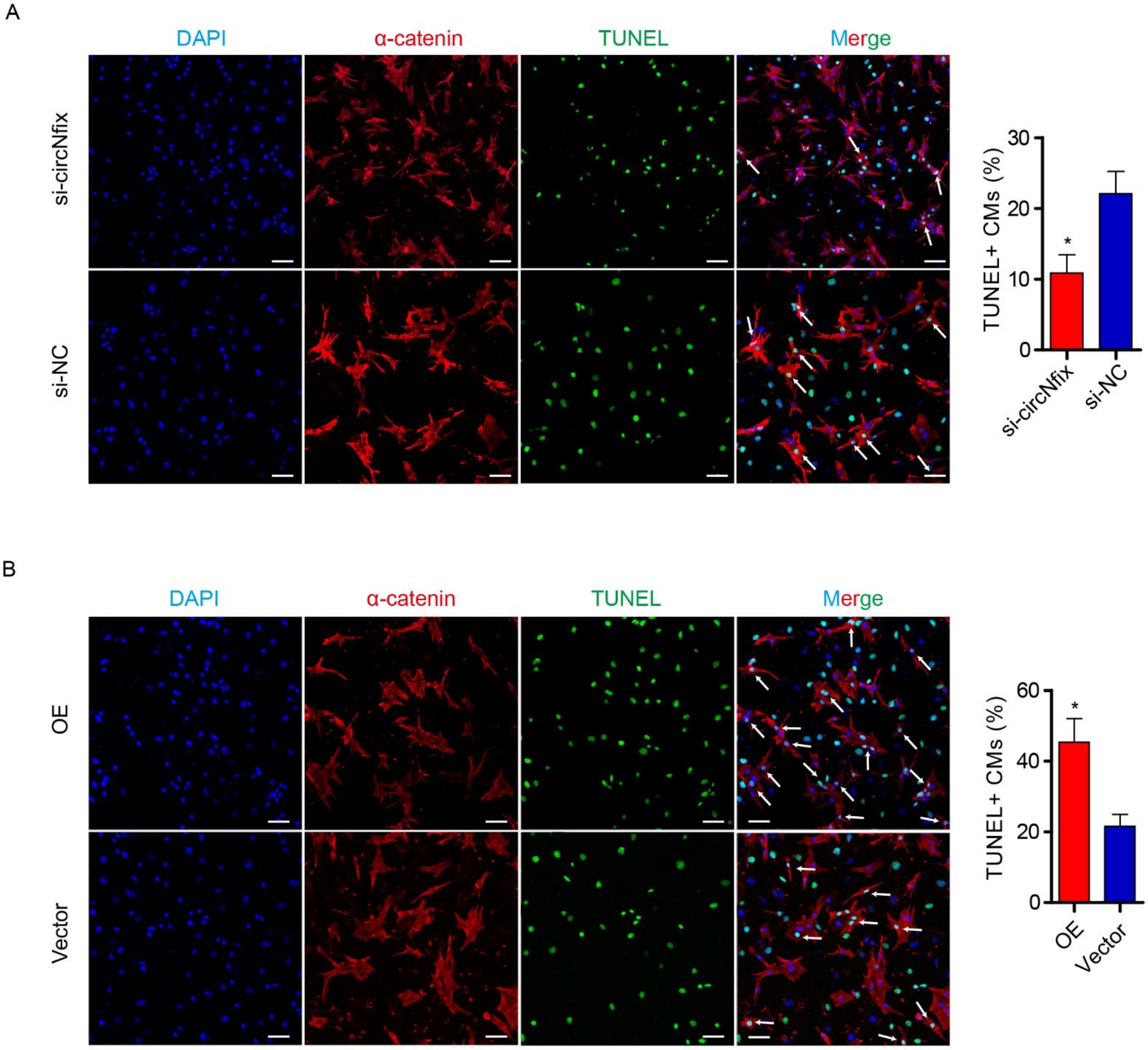

Supplemental Figure 7

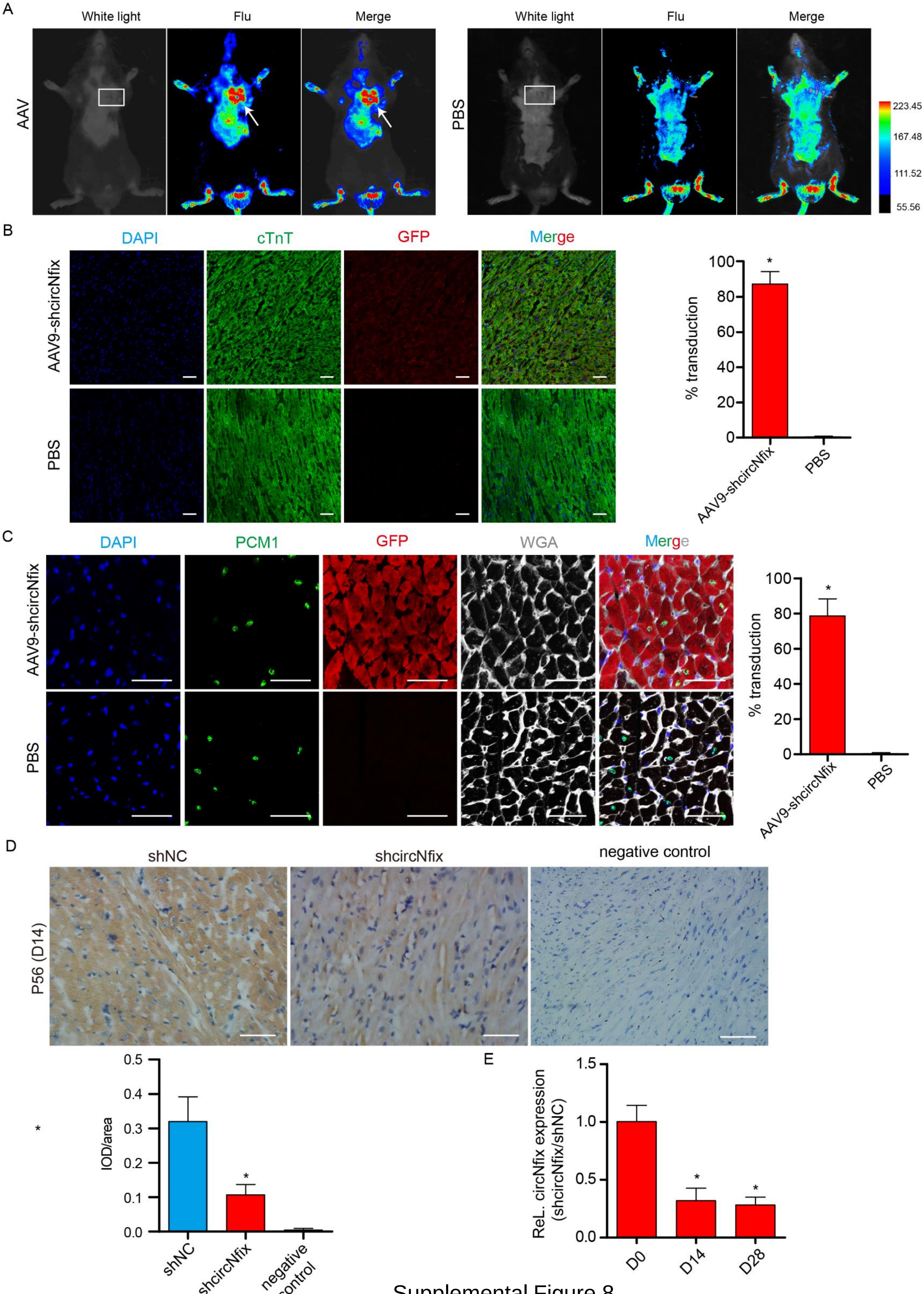

Supplemental Figure 8

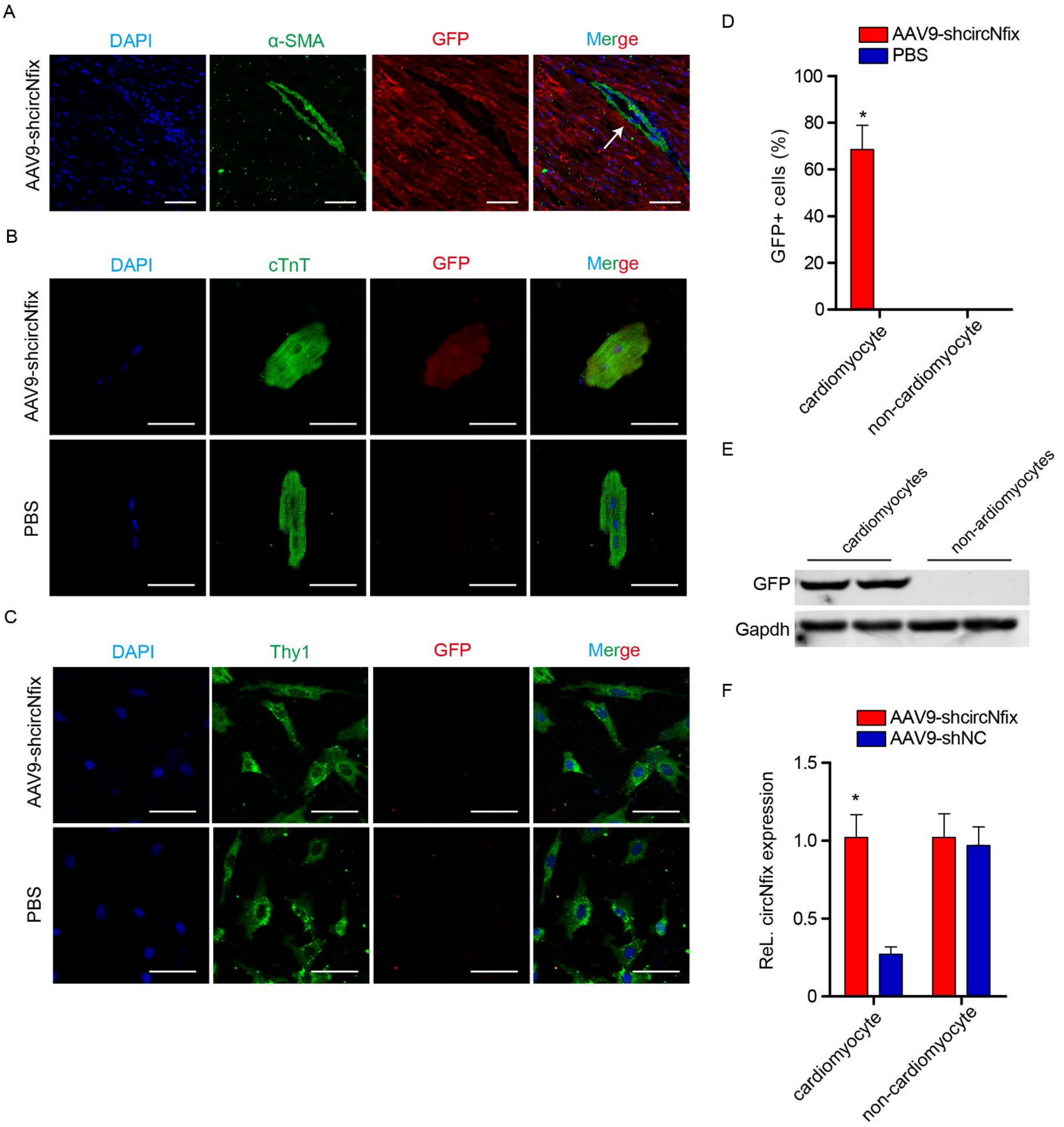

Supplemental Figure 9

A

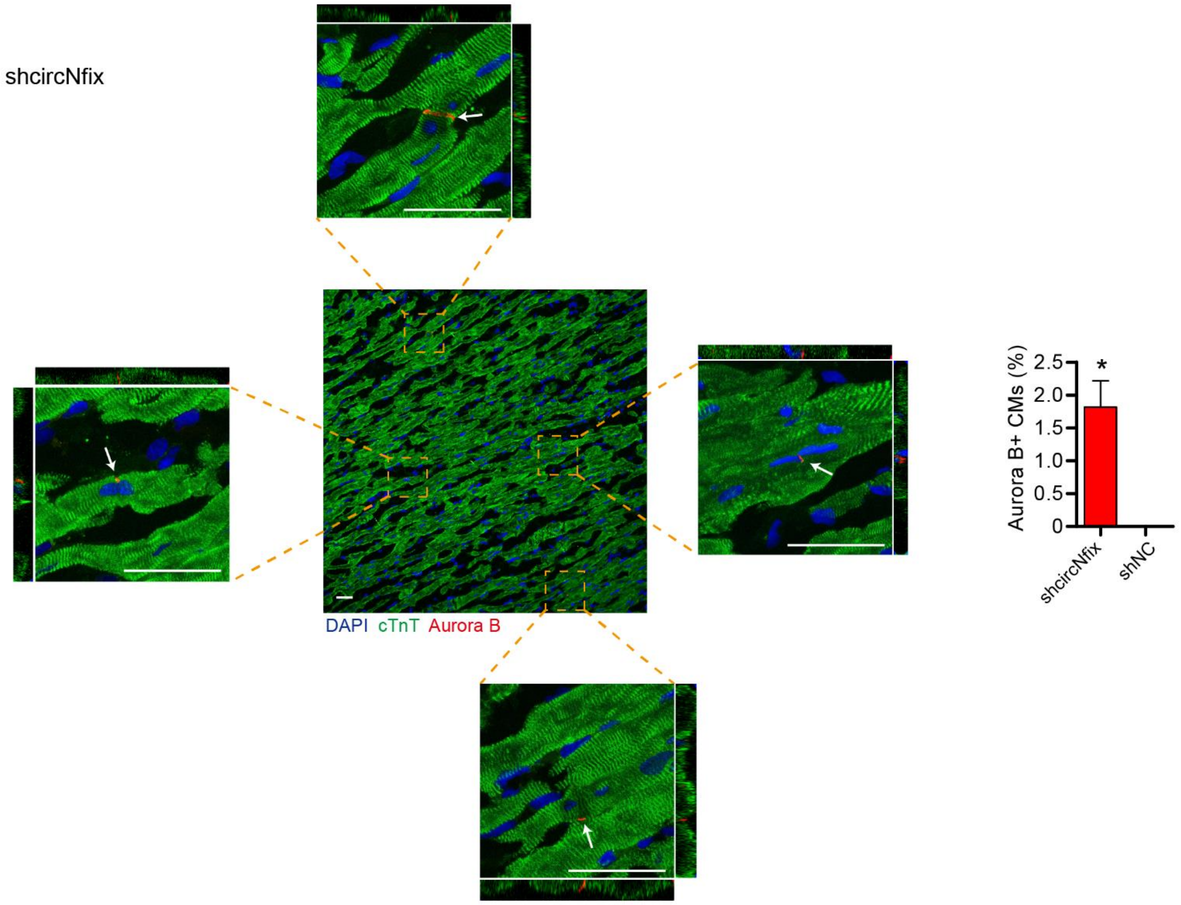

B

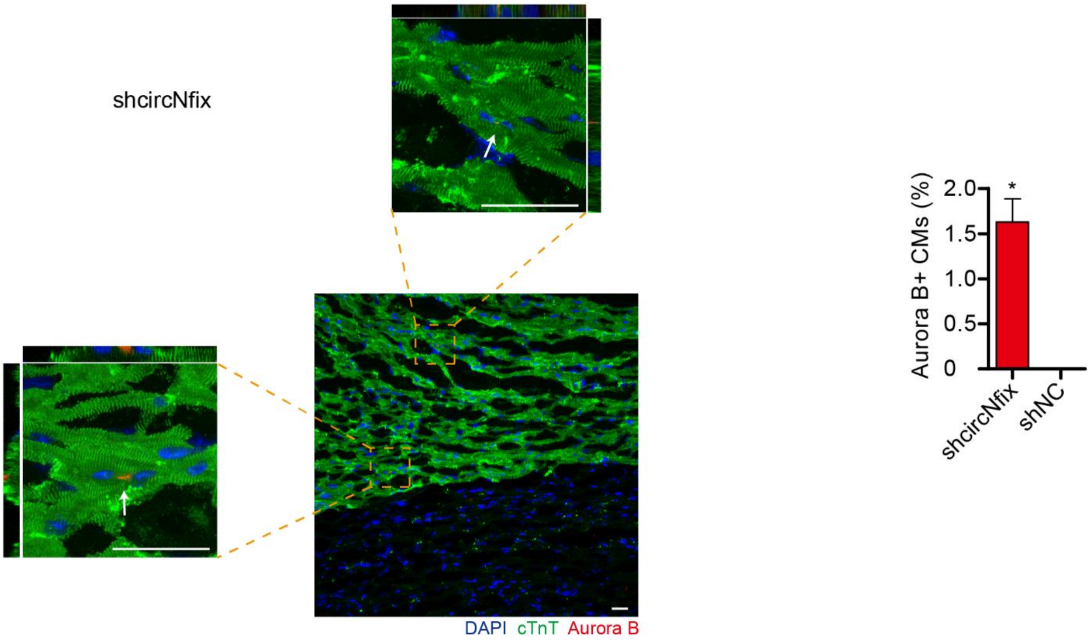

Supplemental Figure 10

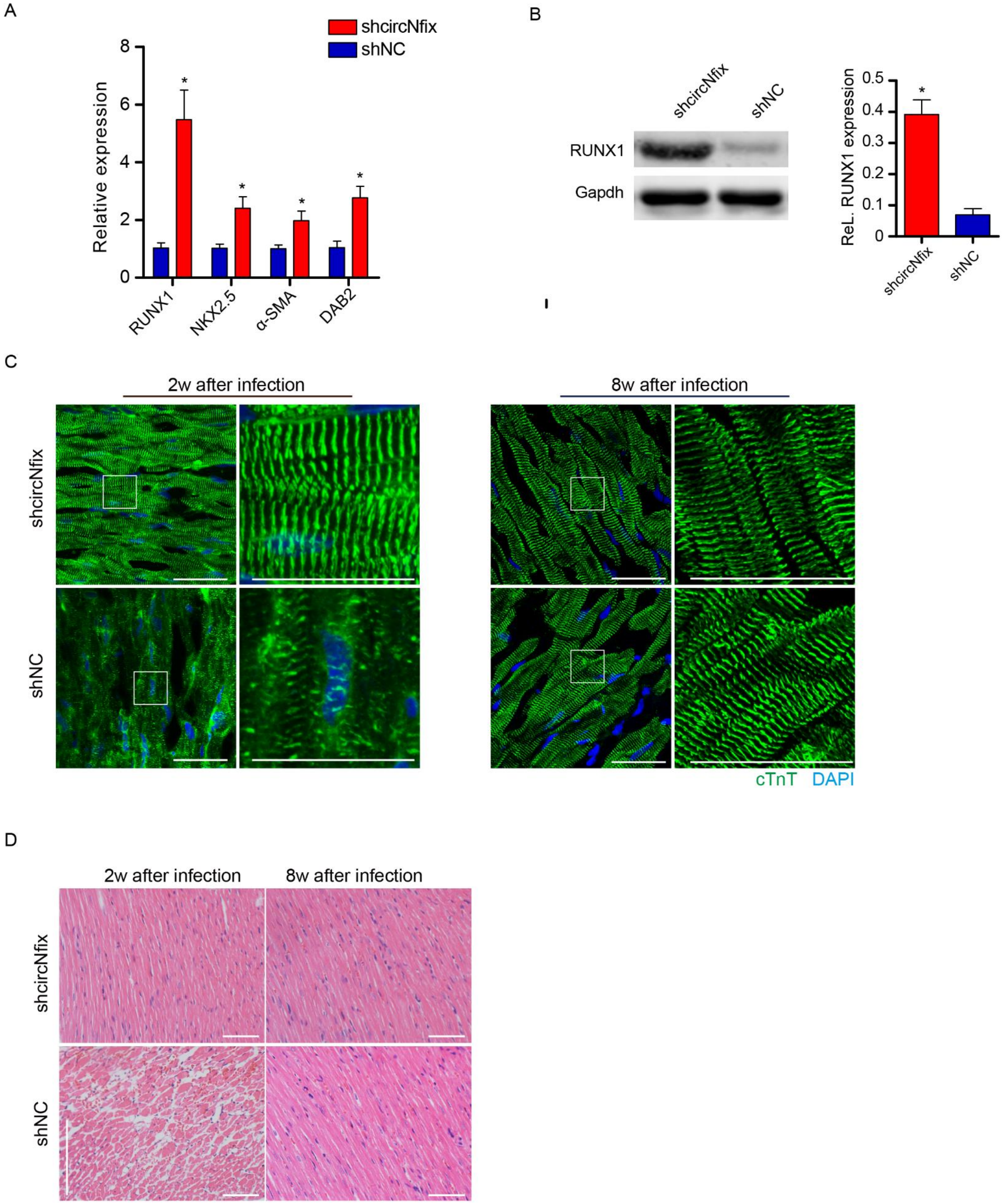

Supplemental Figure 11

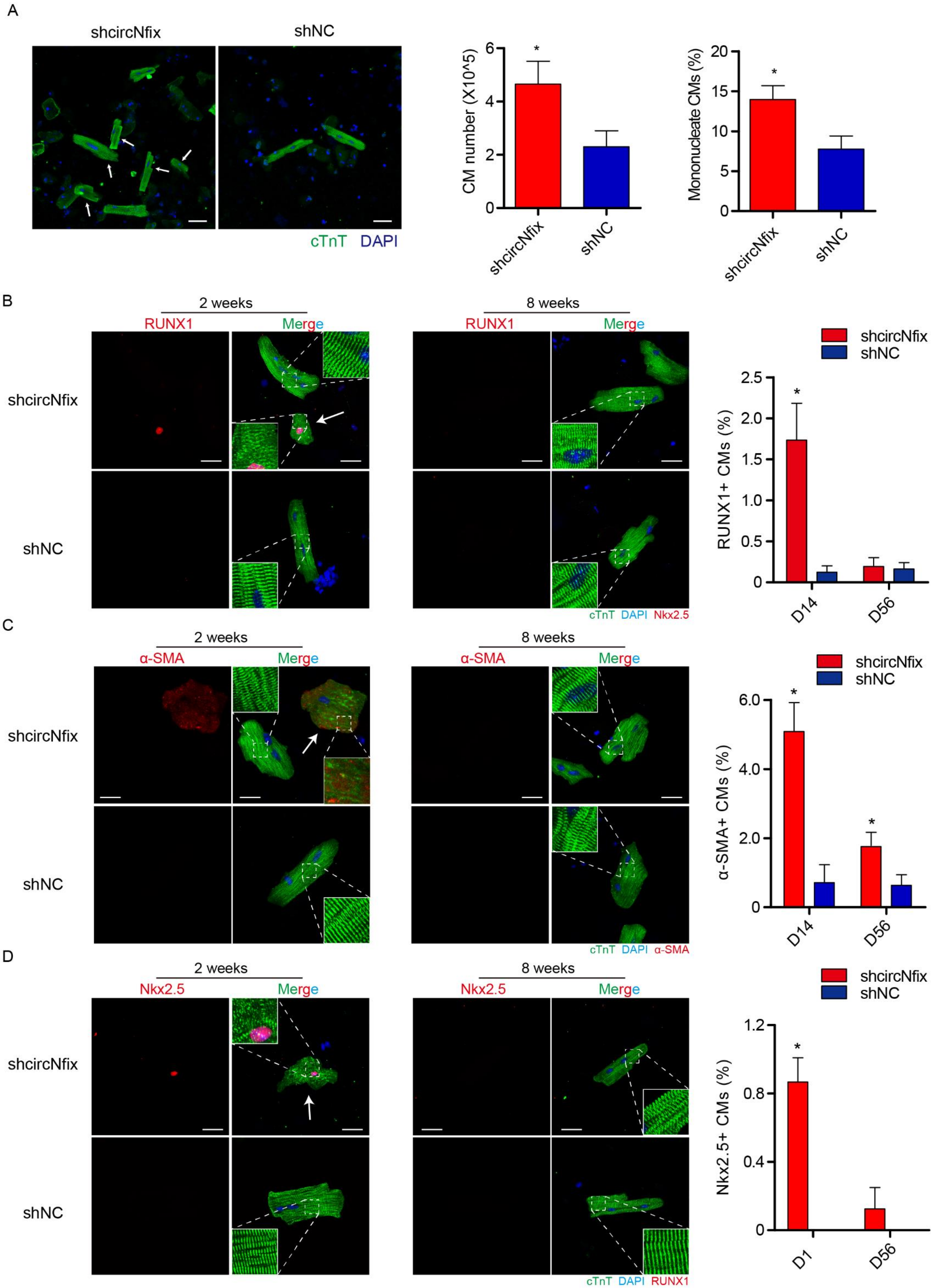

Supplemental Figure 12

E

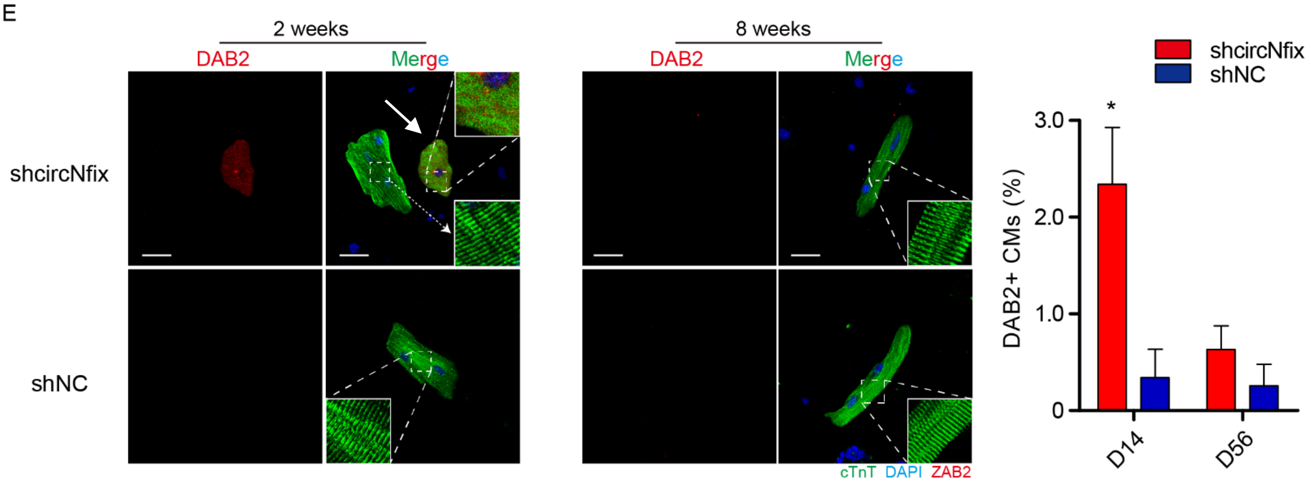

Supplemental Figure 12 Continued

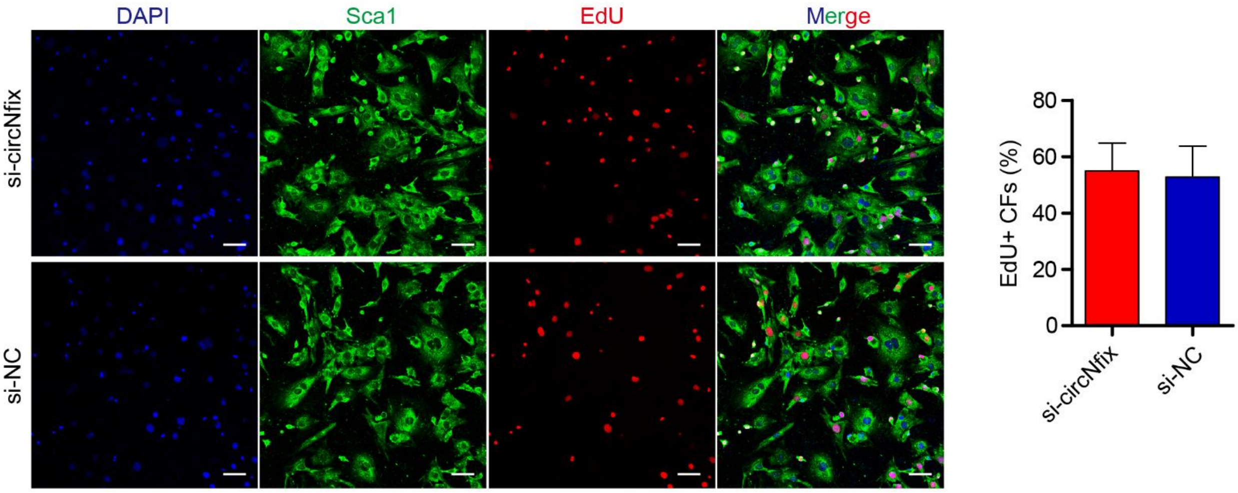

Supplemental Figure 13

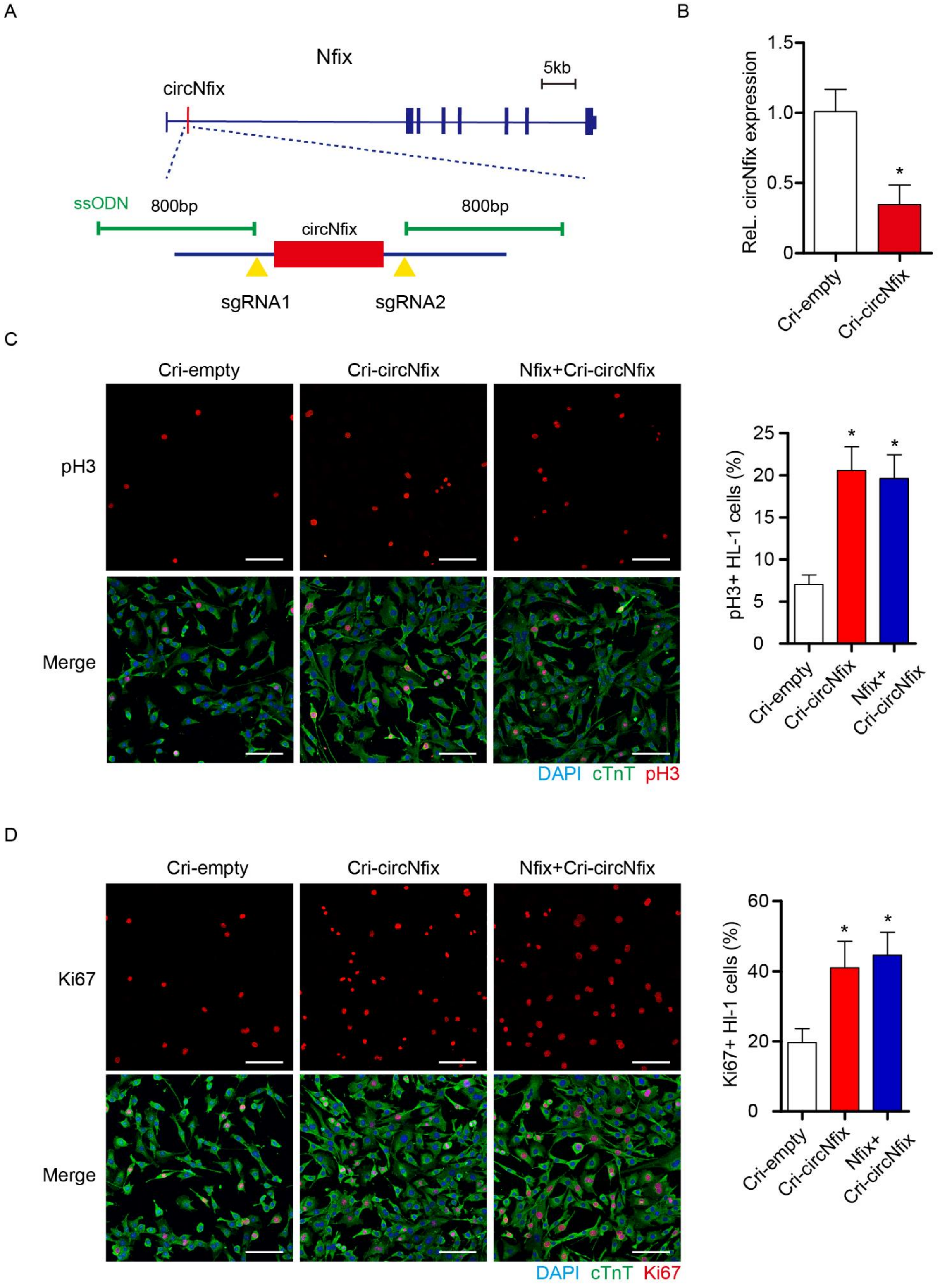

Supplemental Figure 14

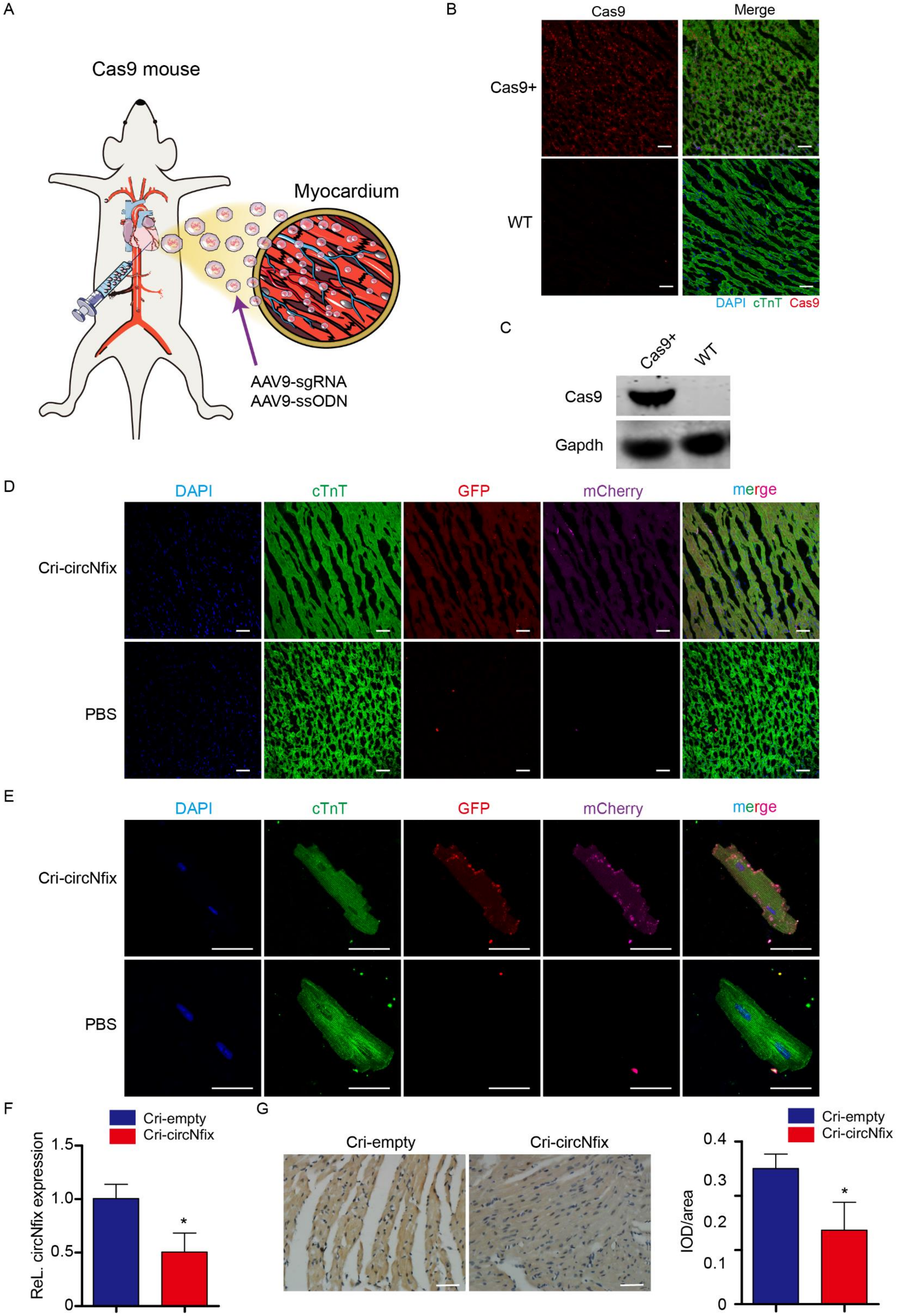

Supplemental Figure 15

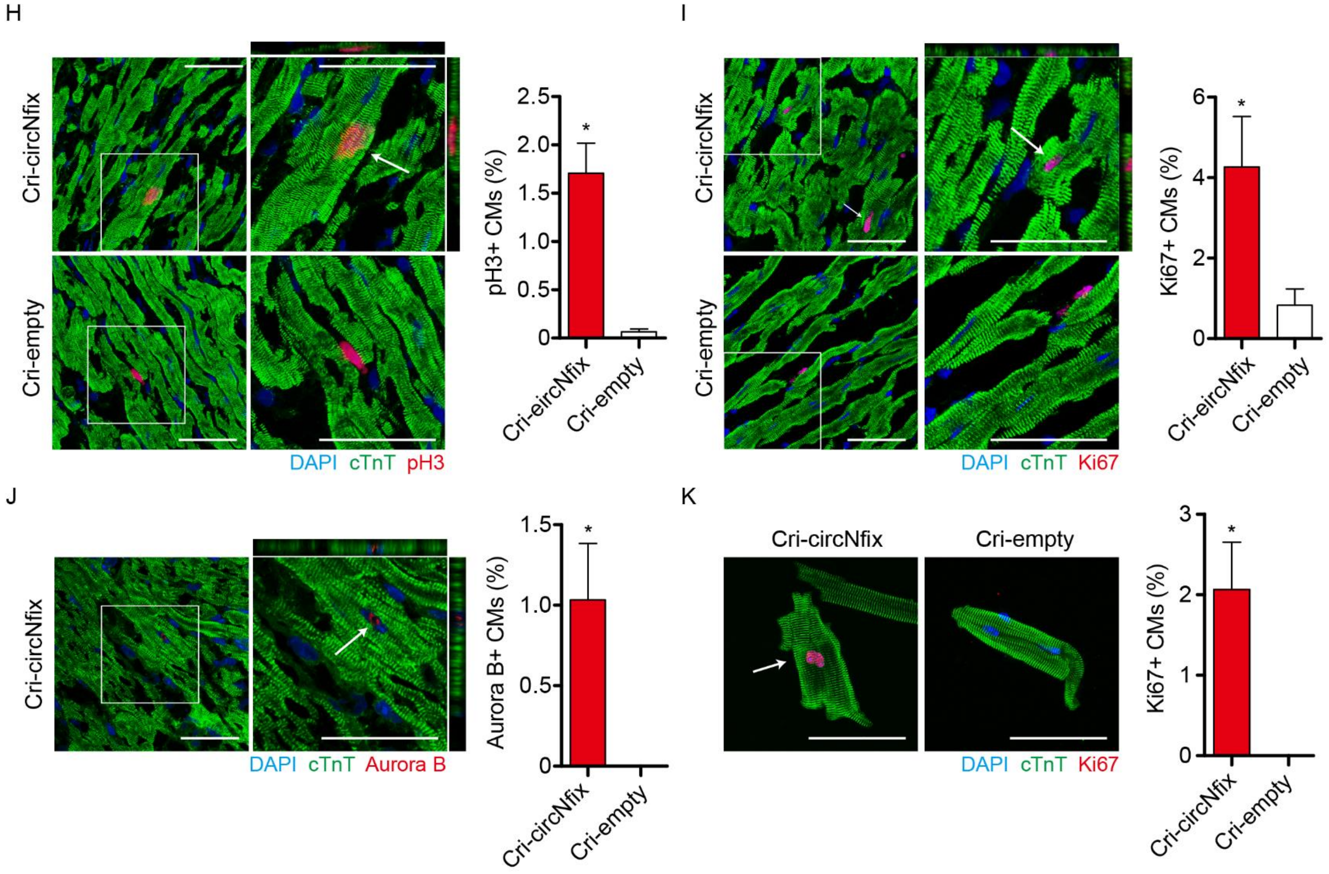

Supplemental Figure 15 Continued

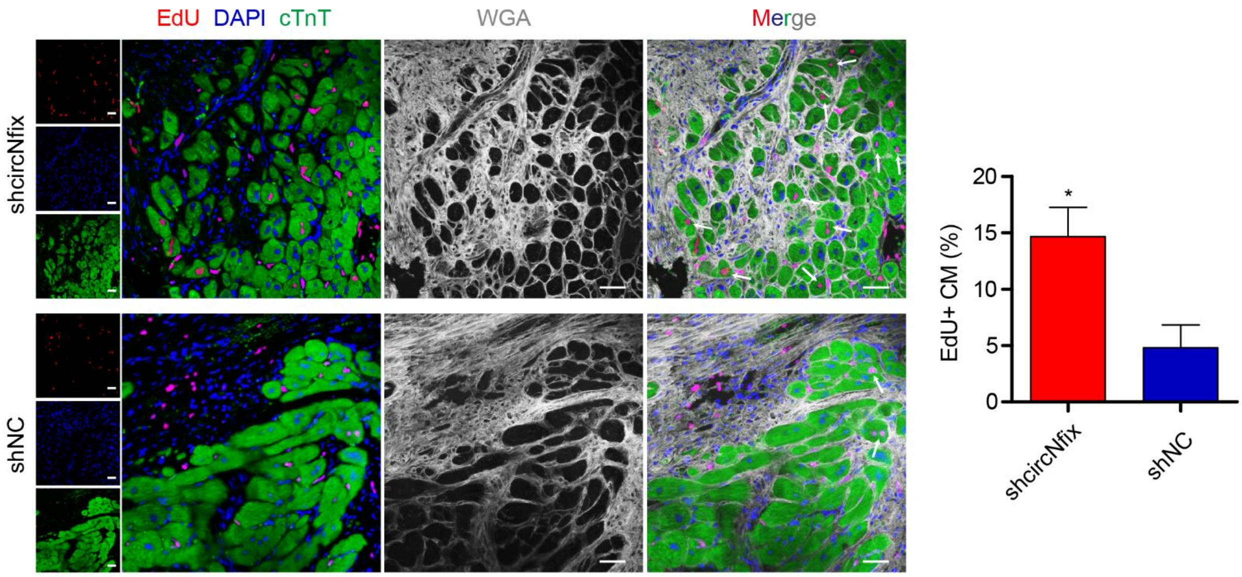

Supplemental Figure 16

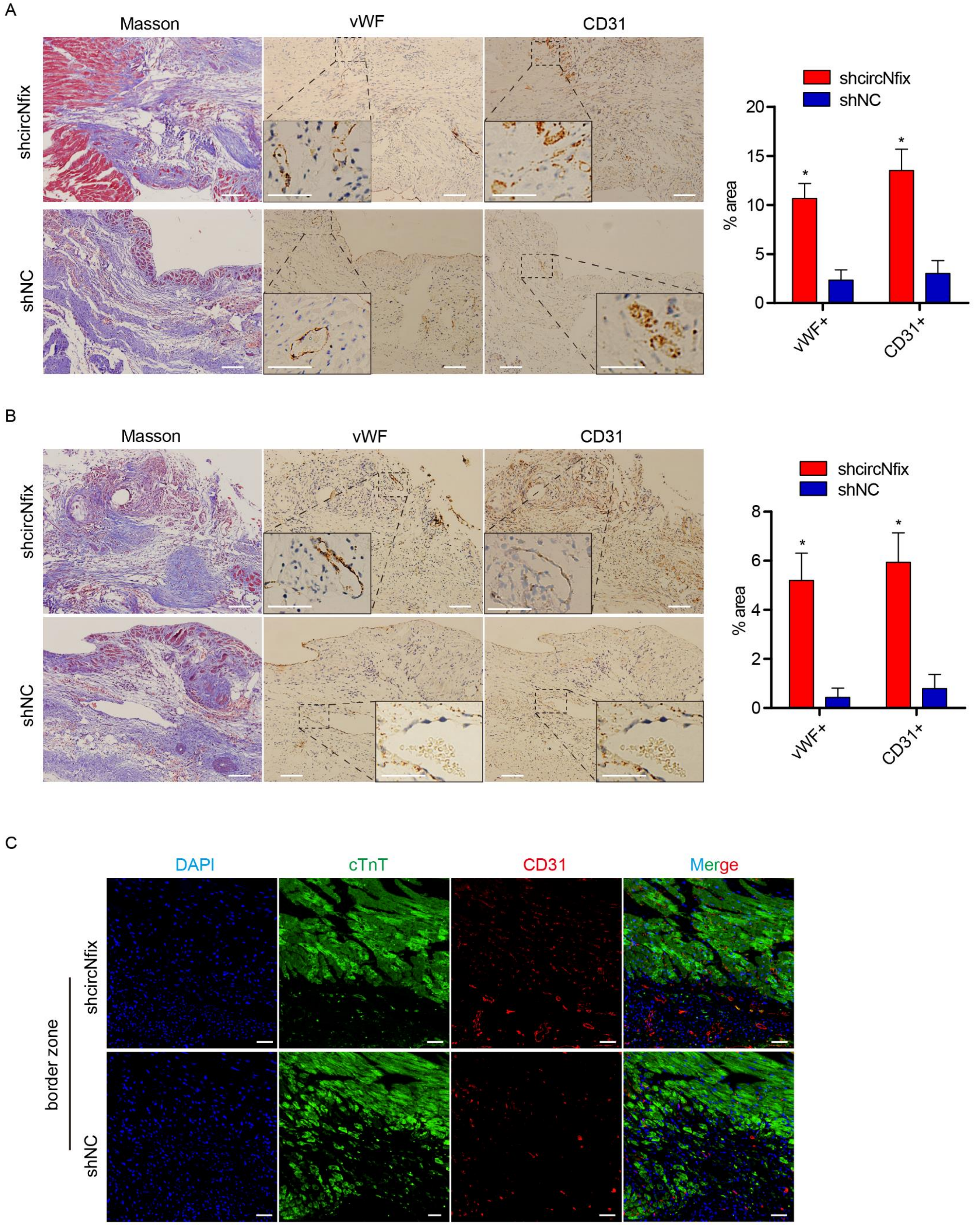

Supplemental Figure 17

D

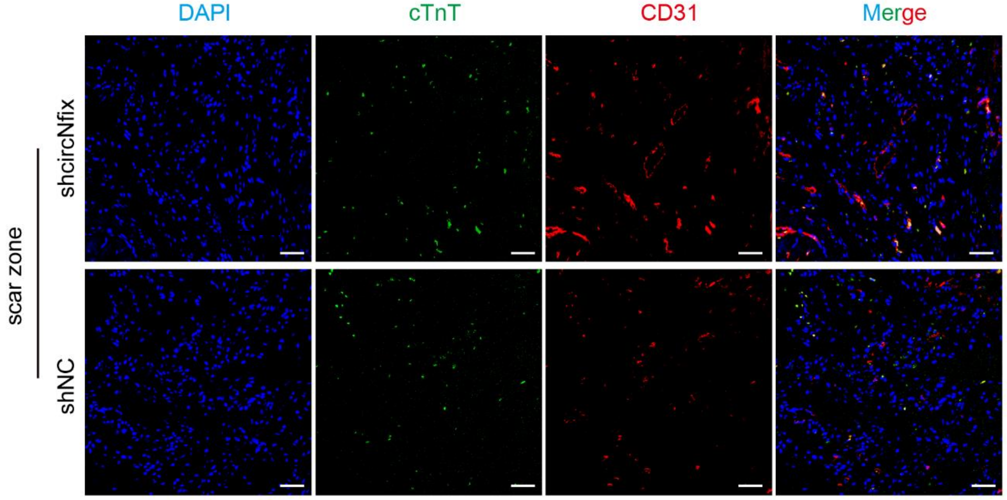

E

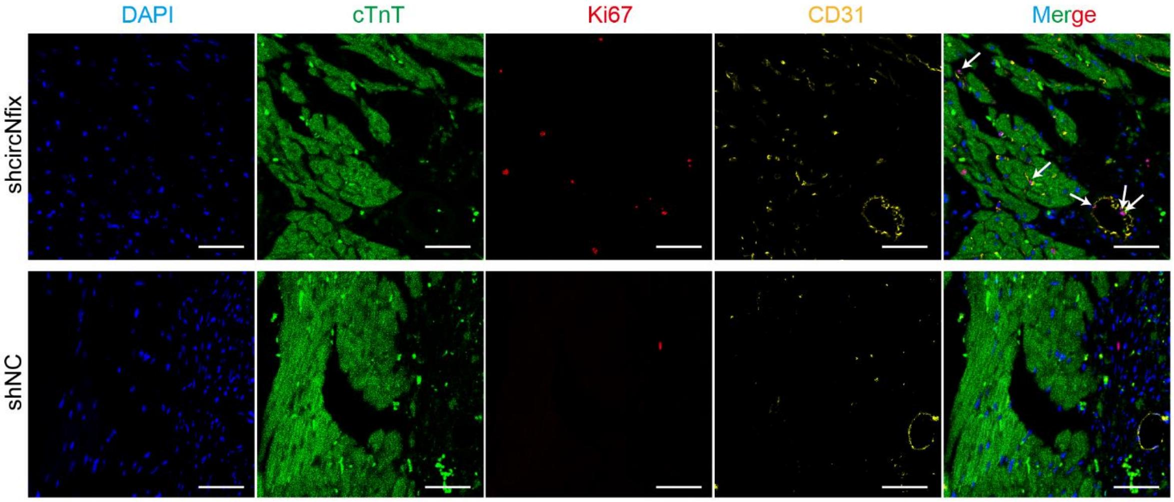

F

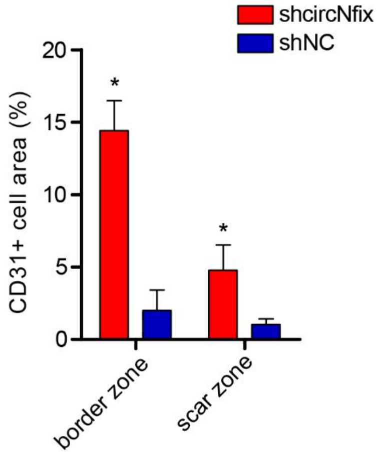

G

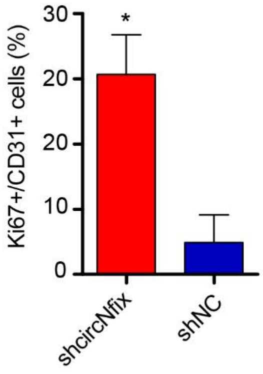

Supplemental Figure 17 Continued

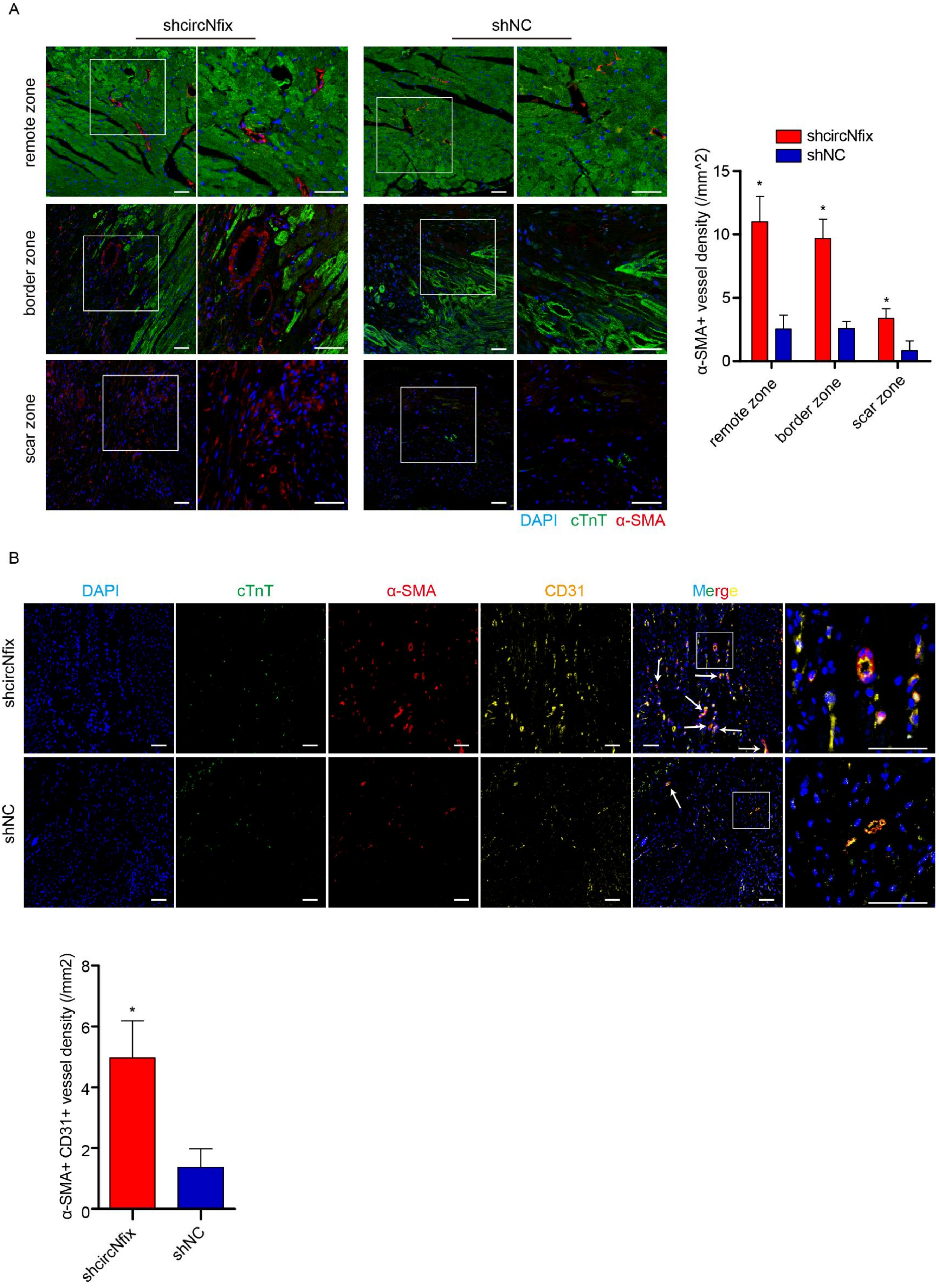

Supplemental Figure 18

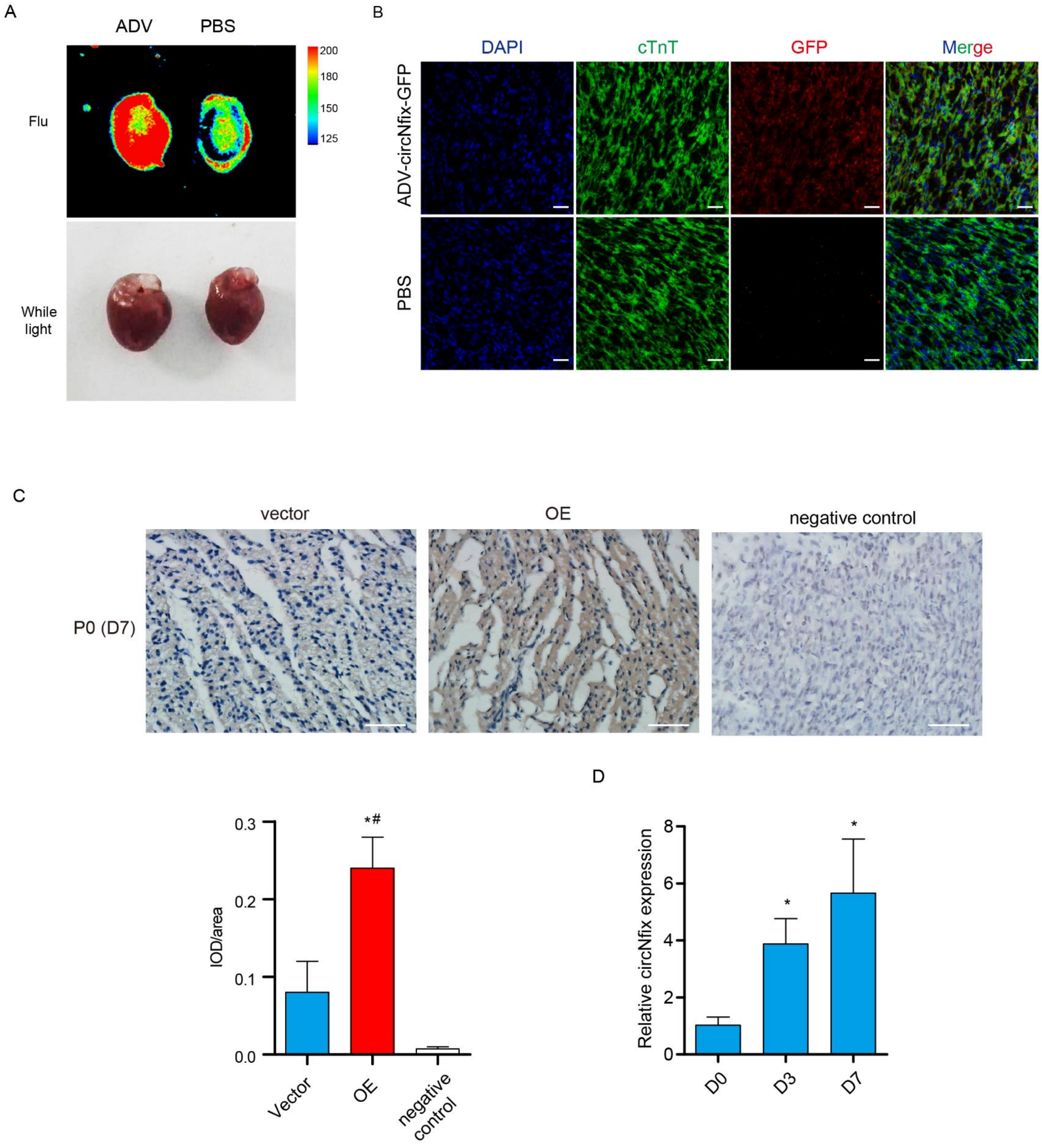

Supplemental Figure 19

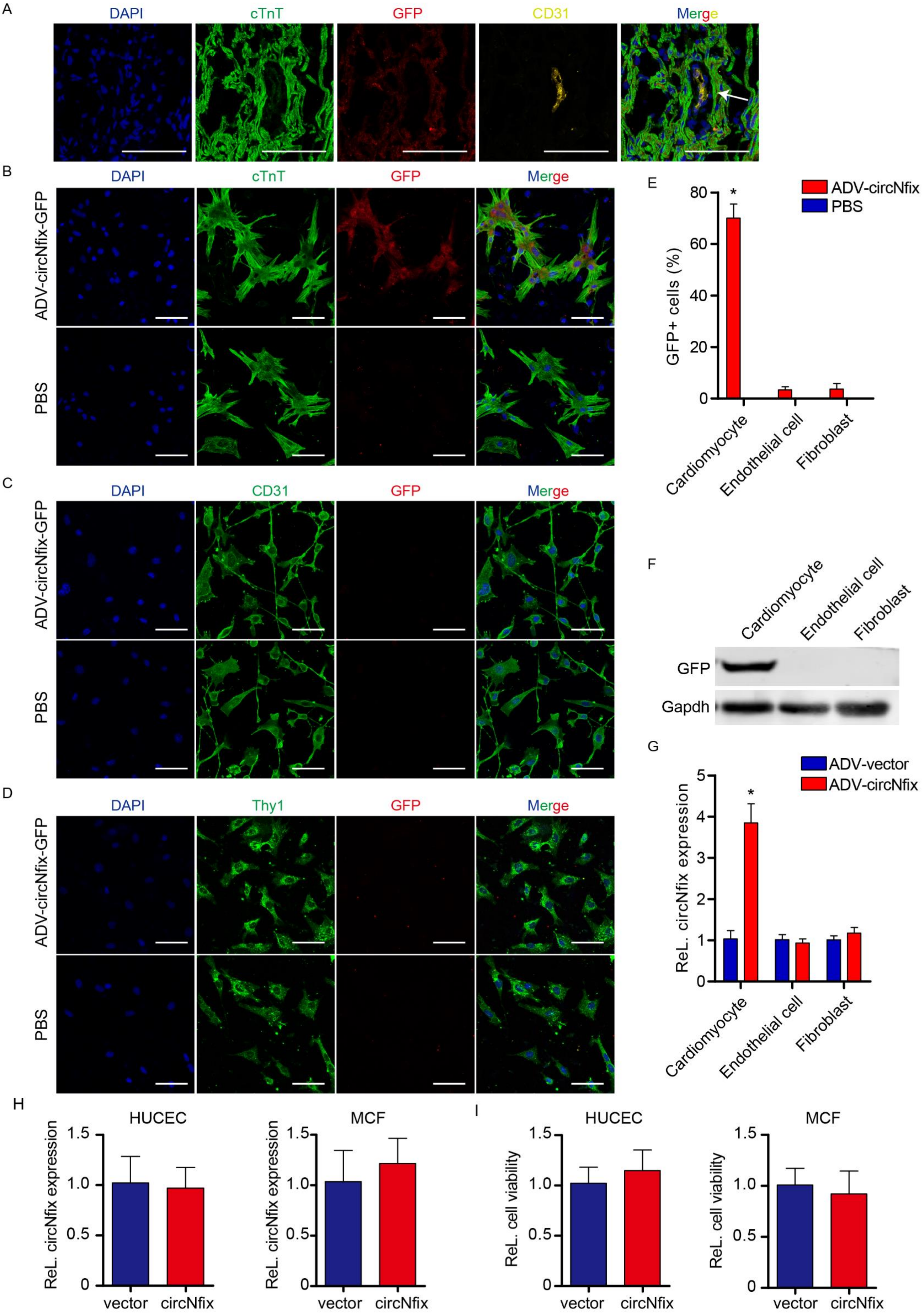

Supplemental Figure 20

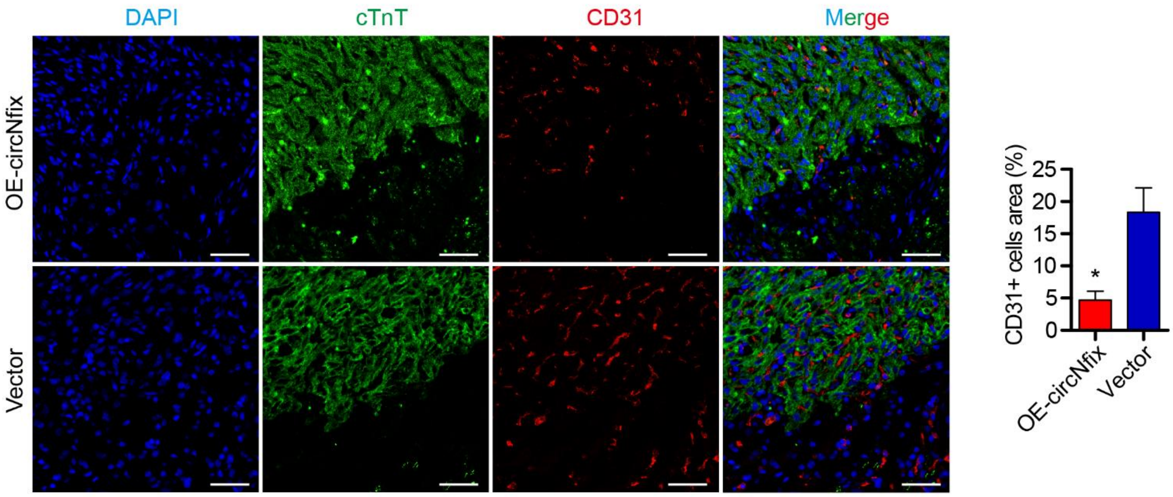

Supplemental Figure 21

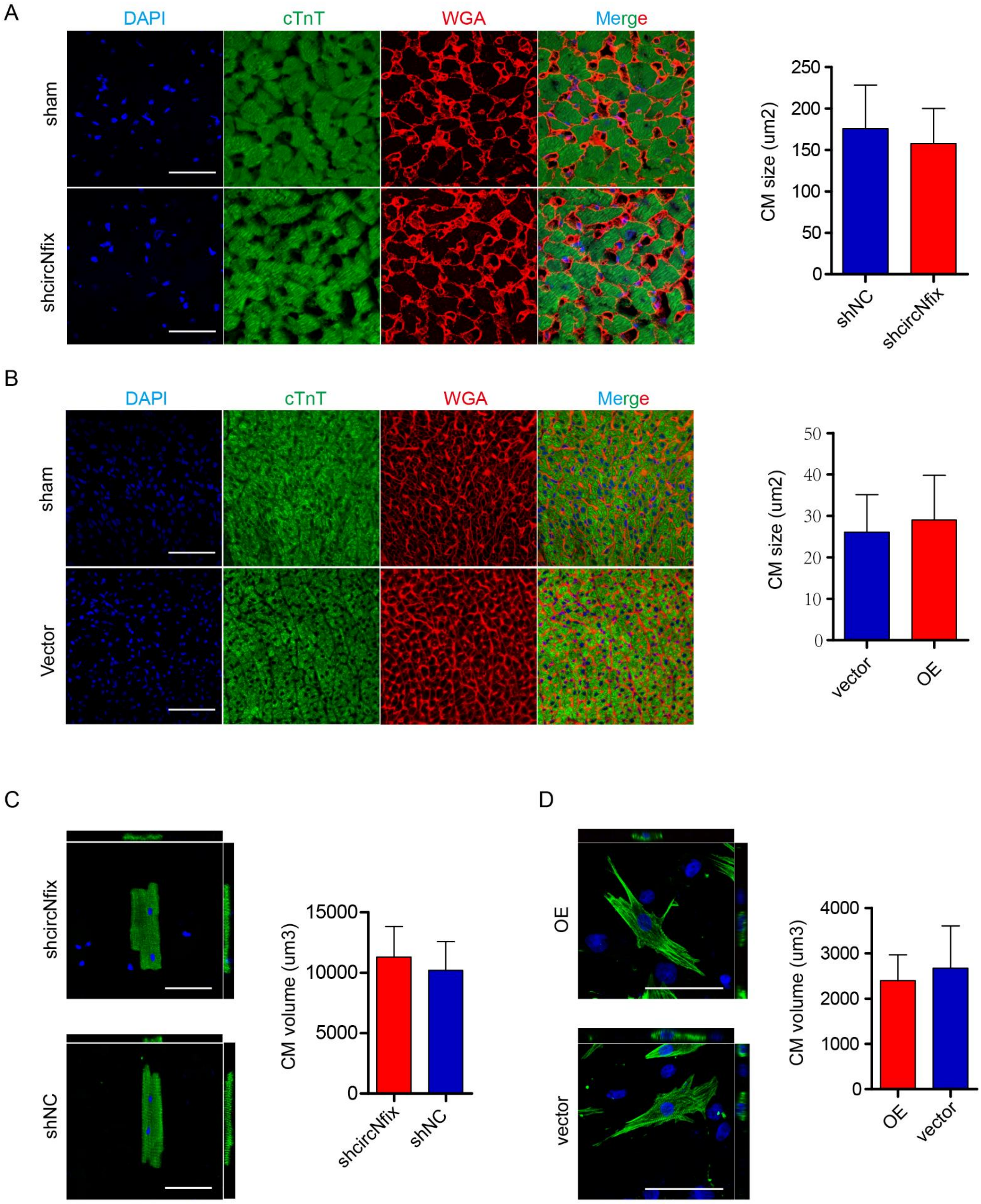

Supplemental Figure 22

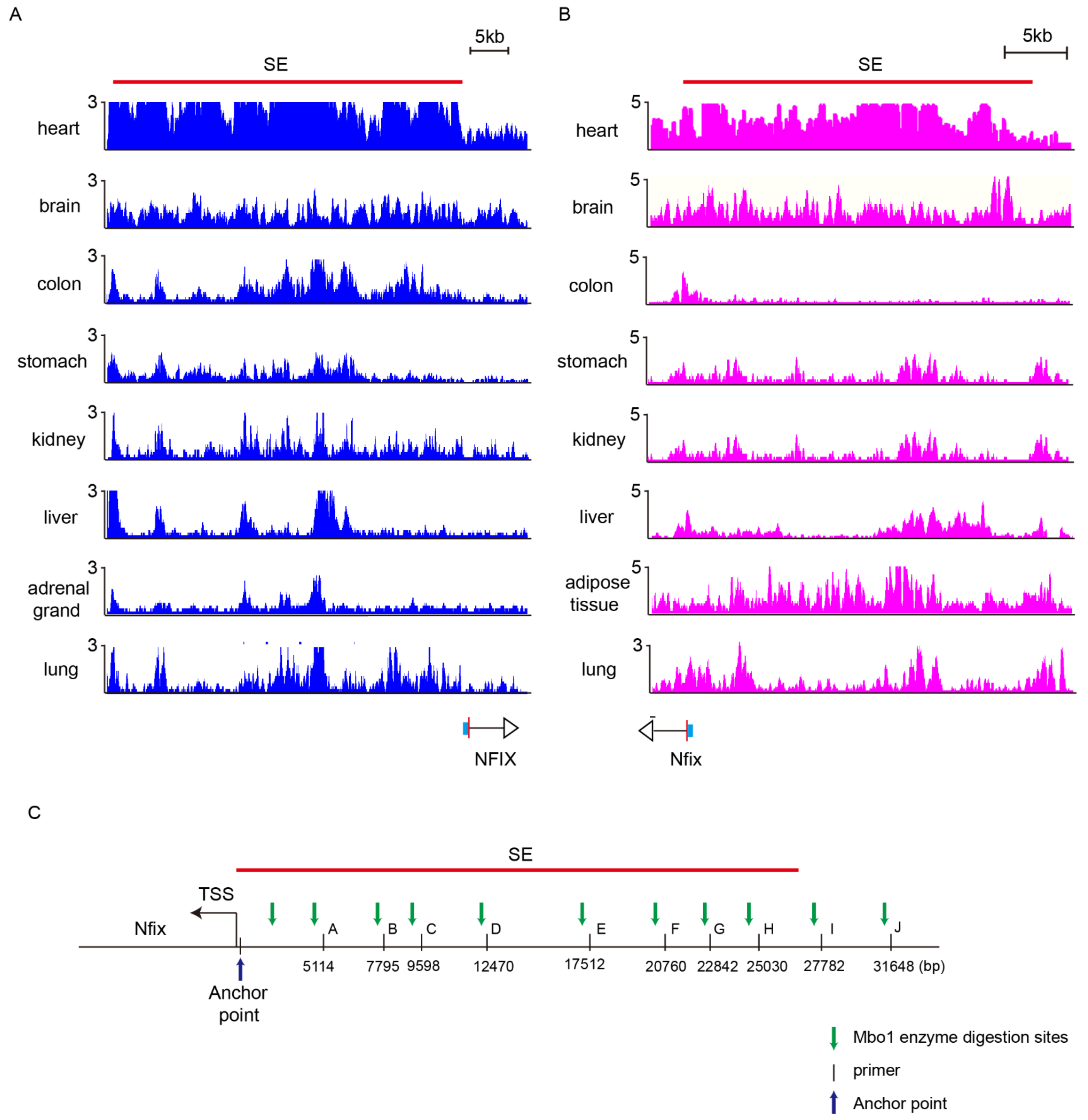

Supplemental Figure 23

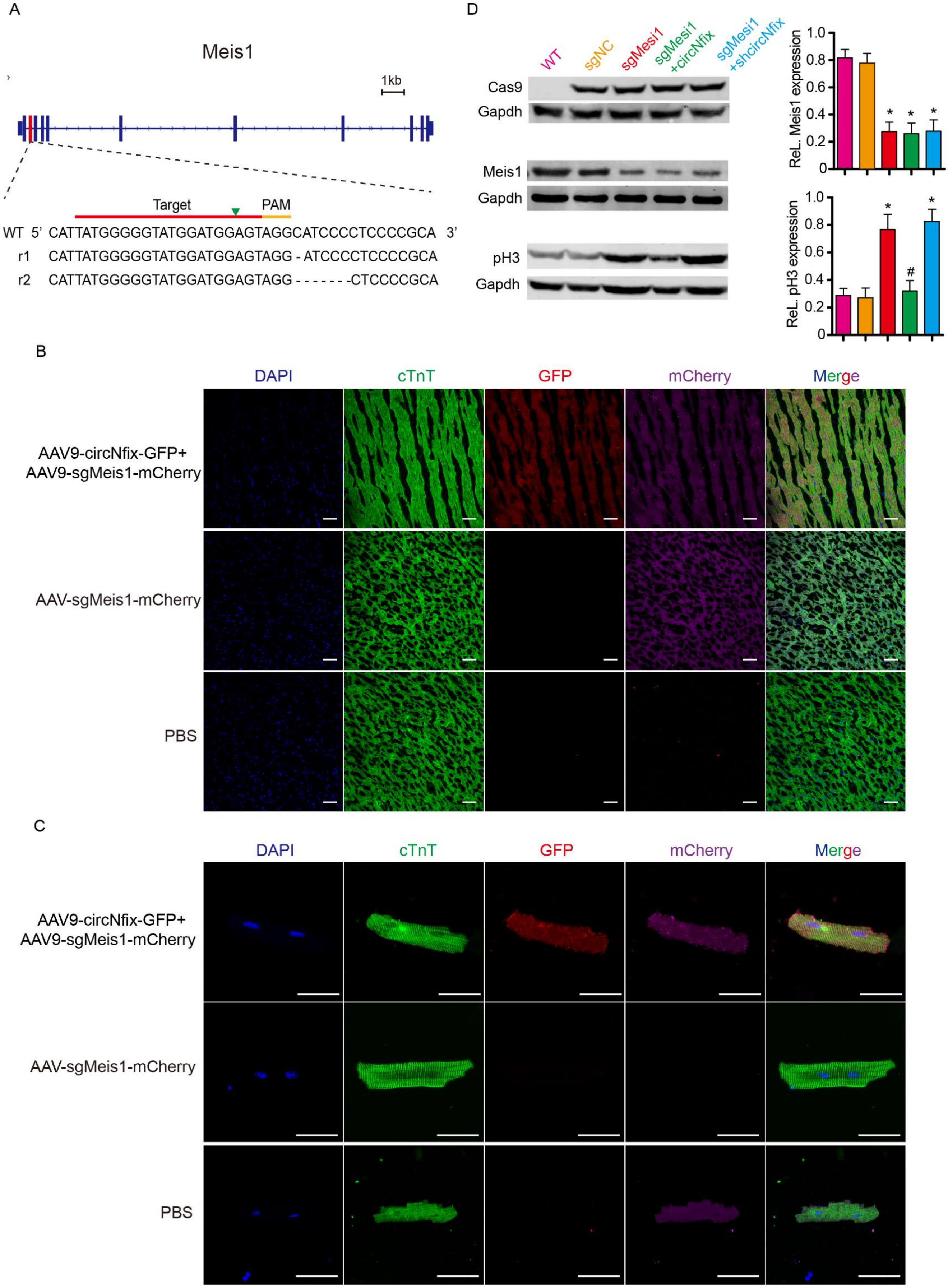

Supplemental Figure 24

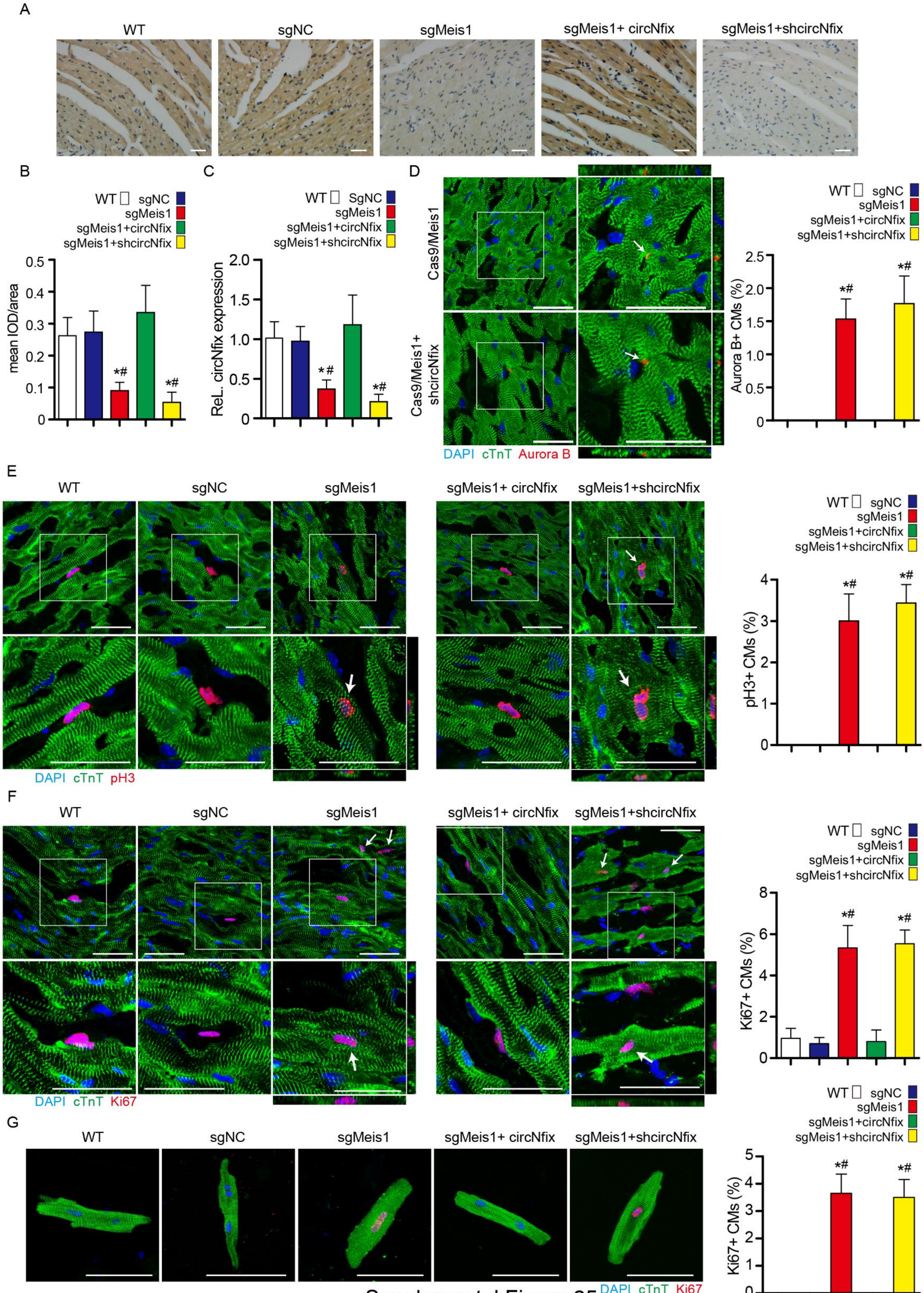

Supplemental Figure 25

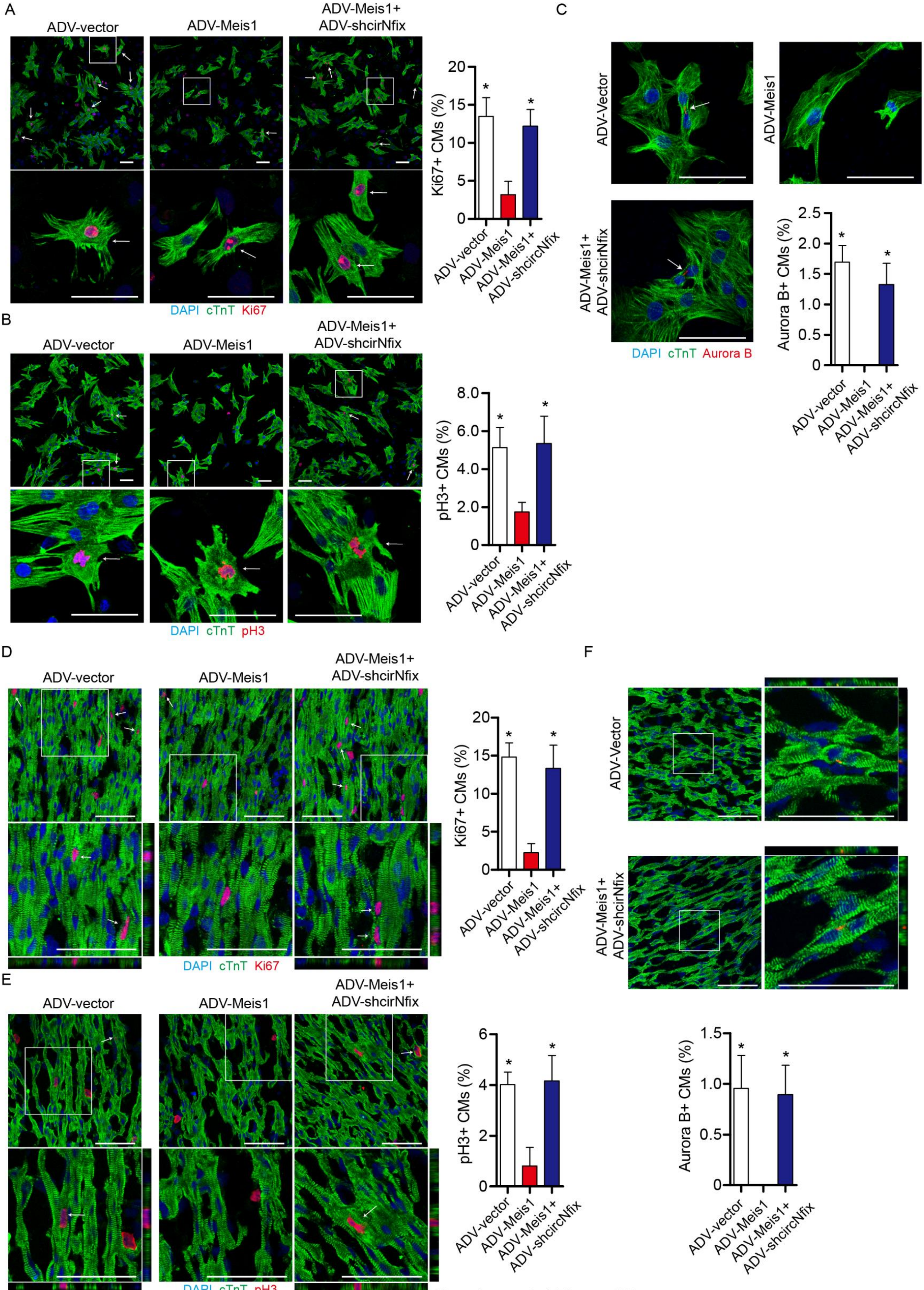

Supplemental Figure 26

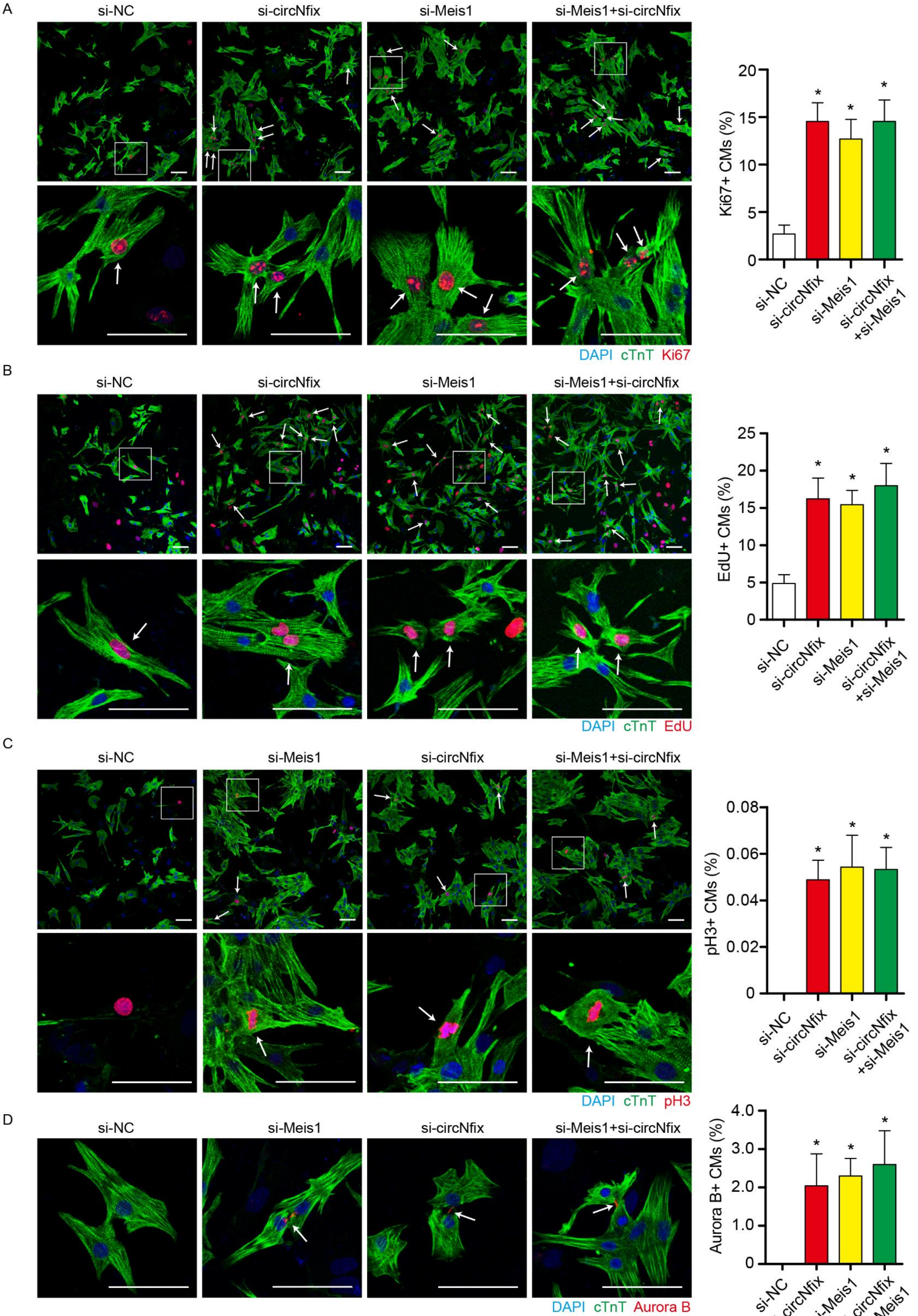

Supplemental Figure 27

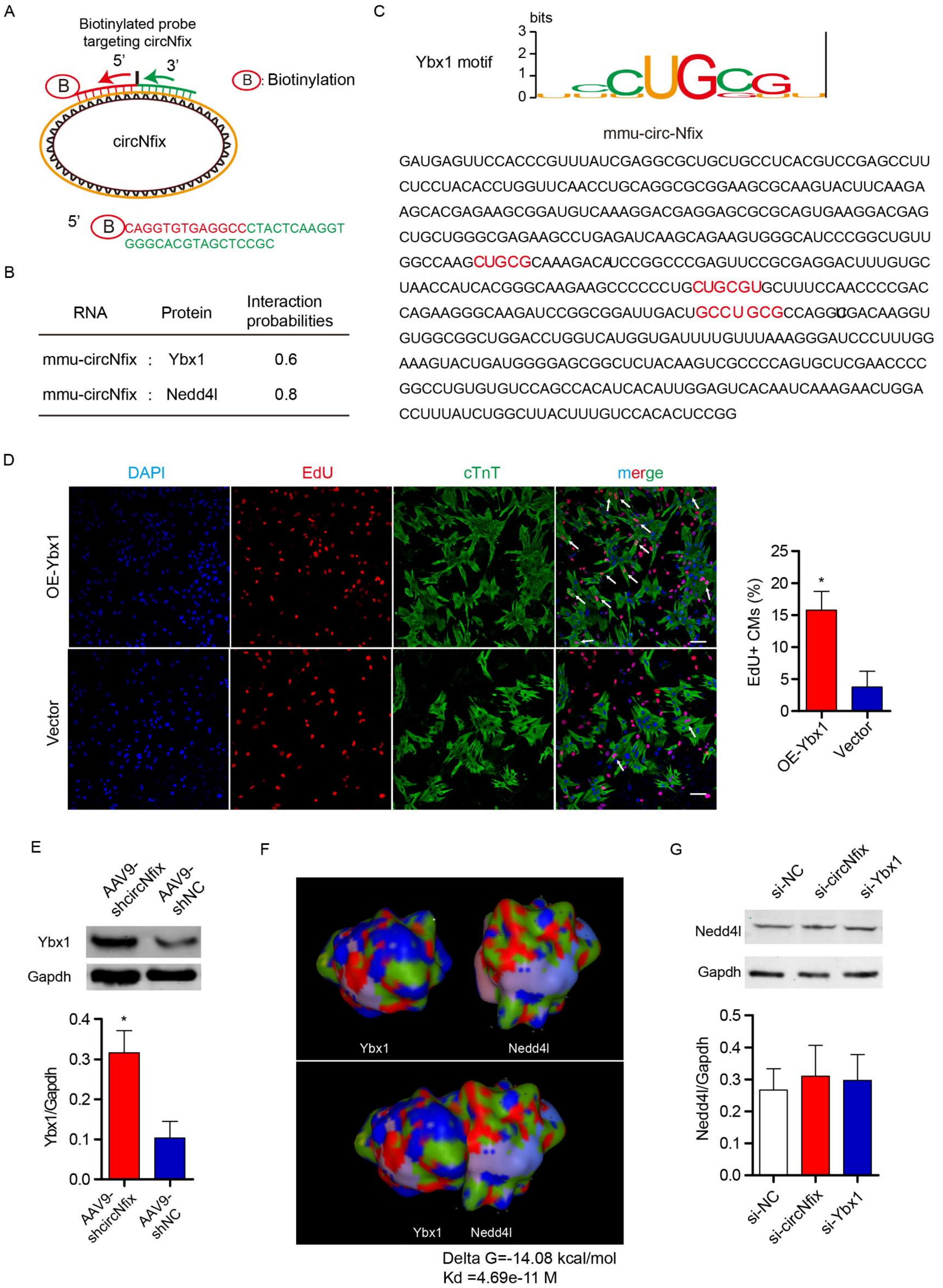

Supplemental Figure 28

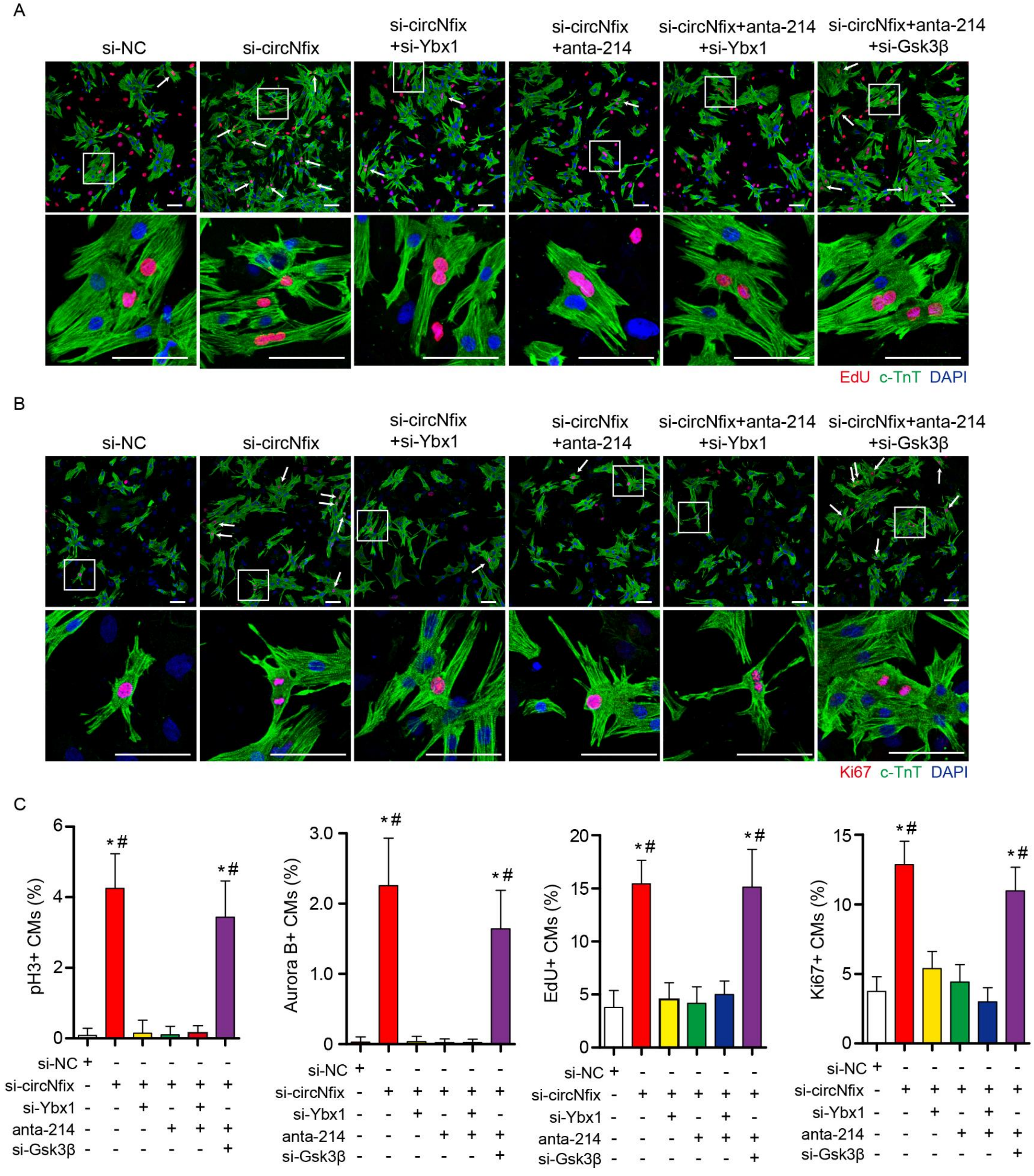

Supplemental Figure 29

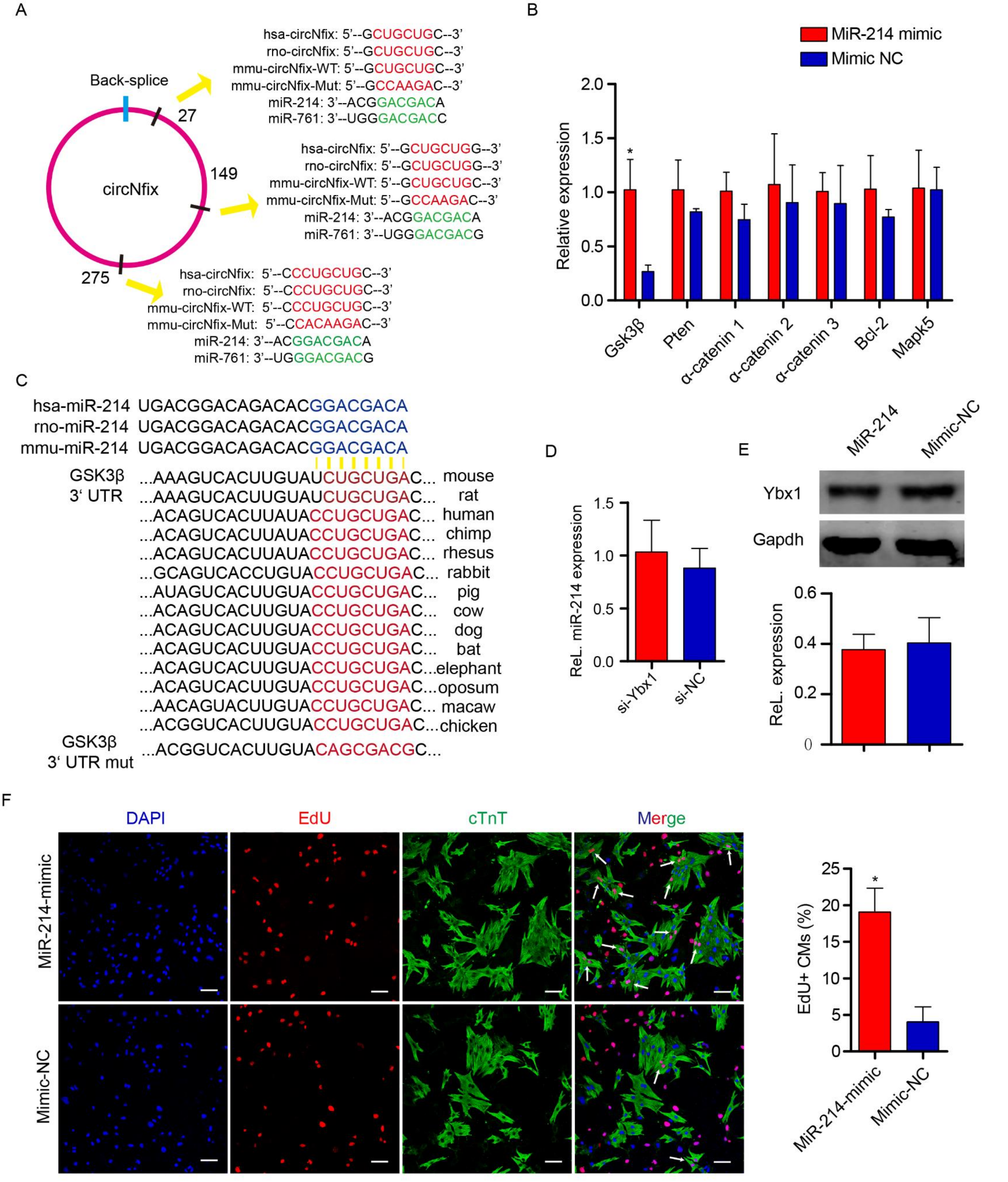

G

Supplemental Figure 30

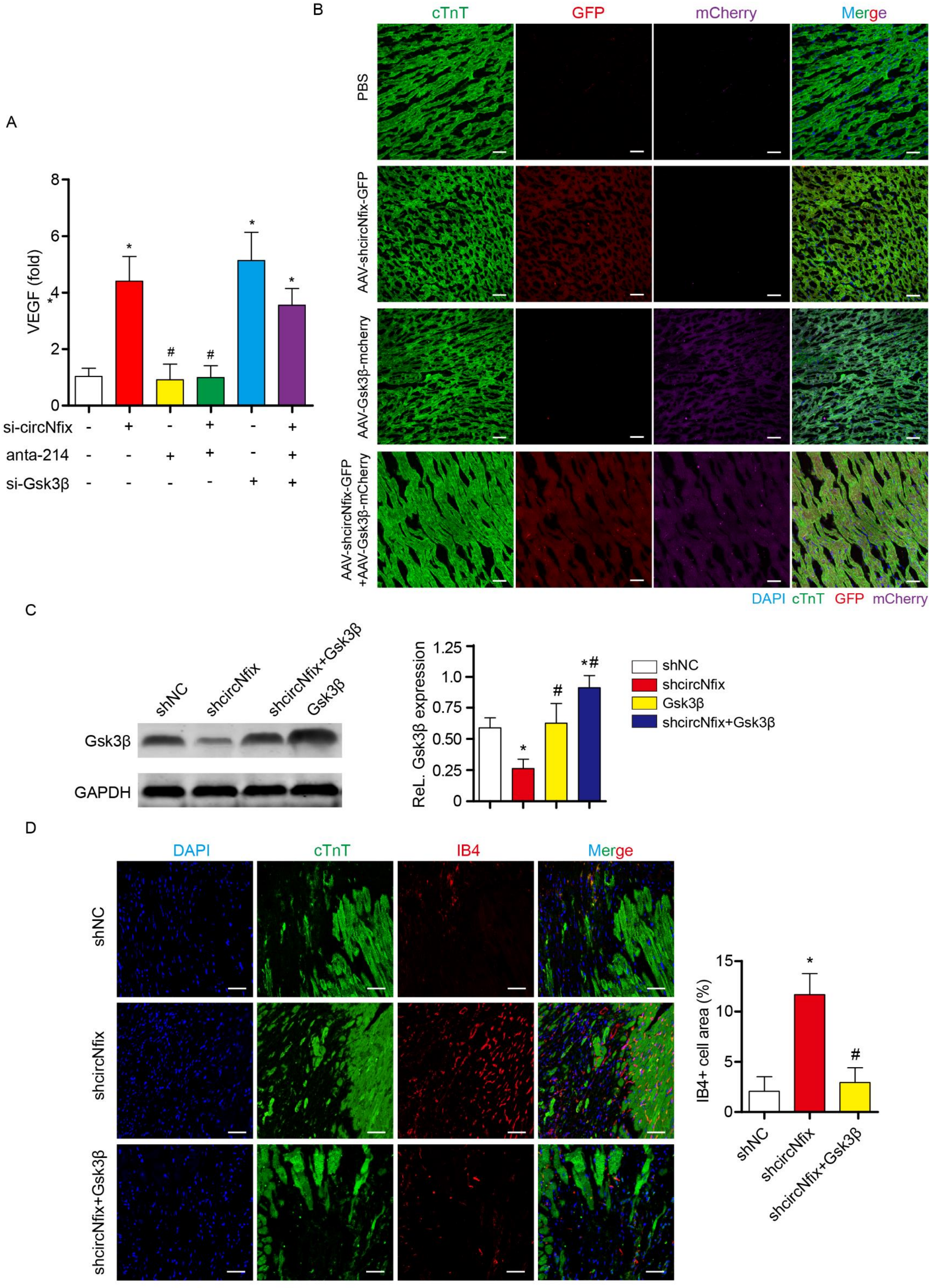

Supplemental Figure 31

E

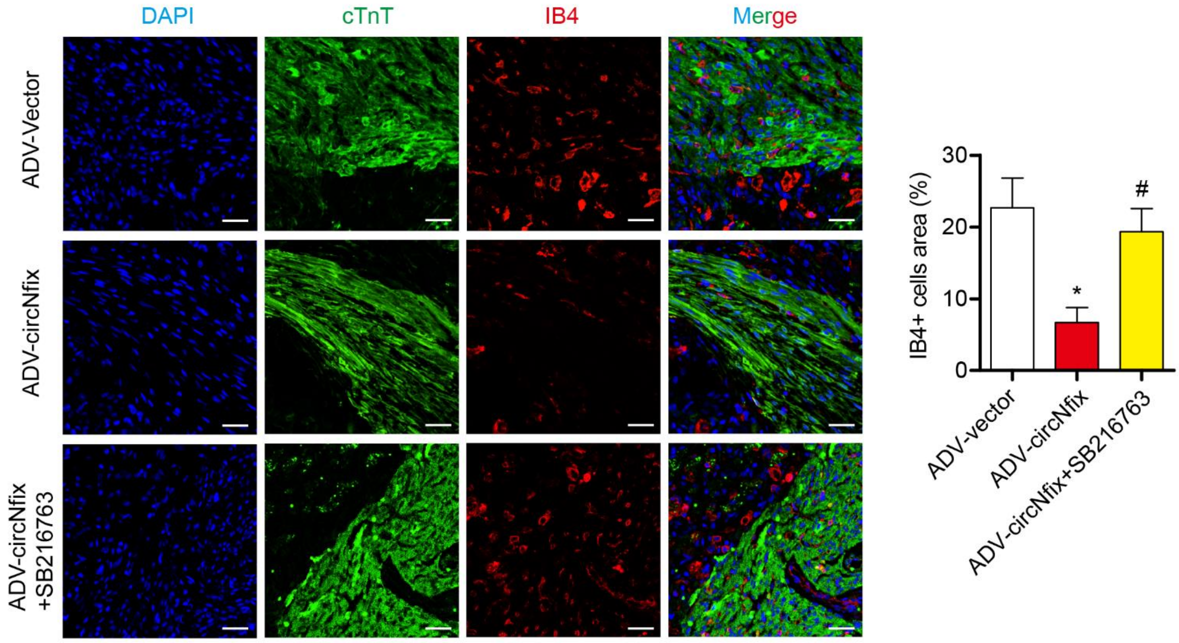

Supplemental Figure 31 Continued

Supplemental figure legends

Supplemental Figure 1 SEs mark tissue-specific and abundant circRNAs. (A) Expression of SE-associated, TE-associated and other circRNAs. **P*<0.05 vs. others, #*P*<0.05 vs. TE, (Kruskal-Wallis test with Dunn's multiple comparison Test). (B) Venn diagrams of SE-circRNAs and TE-circRNAs in heart (red), brain (green), and colon (blue). (C) Pie charts showing percentage of circRNAs and number of observed tissue types when a circRNA is associated with a SE (SE_circRNA) or a TE (TE_circRNA). (D) Heatmap showing expression of circRNAs associated with tissue-specific SEs across 5 human tissue types. Each row is a circRNA, and the black square indicates the association of circRNAs with SEs in the respective tissue. (E) Comparison of distribution of SEs in multiple adult and fetal mouse tissues. (F) Gene ontology terms for cardiac SE-associated genes with corresponding *P* values.

Supplemental Figure 2 Selection strategy of SE-associated circRNAs from a genome-wide profiling of the cardiac transcriptome.

Supplemental Figure 3 Linear Nfix RNA are not involved in CM proliferation. (A)Left: Row normalized Z score heatmap showing Nfix and Slc8a1 mRNA expression level in neonatal (P3) and adult mouse hearts. Right: QRT-PCR assays detecting Nfix mRNA expression in CMs isolated from P0, P7, P56 mouse heart. **P*<0.05 vs. P0, n=6. (B) Illustration of three targeted siRNAs. SiRNA-circNfix (si-circNfix) targets the back-splice junction of circNfix, siRNA-Nfix (si-Nfix) targets

the linear Nfix mRNA, and si-both targets both the linear and circular transcripts. (C) The effect of siRNAs transfection on circNfix expression. **P*<0.05 vs. si-NC, n=5. (D-E) The effect of si-Nfix and si-both transfection on P7 CMs proliferation. White arrows refer EdU-positive CMs. **P*<0.05 vs. si-NC, n=5, bar=50 μm.

Supplemental Figure 4. **CircNfix homology analysis of the mouse, human and rat genome.** (A) CircNfix homology analysis of the mouse and human genome was conducted by using Basic Local Alignment Search Tool (BLAST). (B) CircNfix homology analysis of the mouse and rat genome was conducted by using BLAST.

Supplemental Figure 5 ISH for circNfix in adult and neonatal infarcted mouse heart sections. (A) Detection of circNfix expression by ISH in adult and neonatal infarcted mouse heart sections. Masson staining of corresponding heart sections.

Supplemental Figure 6 Silencing circNfix induces P7 CM proliferation and dedifferentiation. (A) Aurora B immunofluorescence staining in P7 CMs after circNfix knockdown. The images were captured by Leica (TCS Sp8) confocal microscope. Aurora B-positive CMs were indicated by arrows. 507 CMs from 10 mice in si-circNfix group, 458 CMs from 10 mice in si-circNfix group, **P*<0.05, bar=50μm. (B) CM sarcomeric status quantification by cTnT immunofluorescence analysis of P7 CMs transfected with siRNA-circNfix (n=345 CMs pooled from the analysis of 11 mice) and siRNA-NC (n=317 CMs pooled from the analysis of 11

mice); *P<0.05, scale bars=50 μm.

Supplemental Figure 7 The effect of circNfix alteration on CM apoptosis. (A) The effect of circNfix knockdown on CM apoptosis. CM apoptosis detected by TUNEL staining 6h after treated with 50μm H_2O_2. About 300 CMs pooled from the analysis of 11 mice, *P<0.05, bar=50μm. (B) The effect of circNfix overexpression on CM apoptosis. CM apoptosis detected by TUNEL staining 6h after treated with 50μm H_2O_2. About 300 CMs pooled from the analysis of 11 mice, *P<0.05, bar=50μm.

Supplemental Figure 8 The transfection efficiency of AAV9-shcircnfix. (A) *In vivo* bioluminescence images captured on day 14 after injection with GFP-labelled AAV9-shcircNfix virus. Square indicates thoracic incision, arrow indicates the heart with GFP fluorescence. (B) GFP/cTnT double-staining in adult hearts 28 days after injection of AAV9-shcircNfix or PBS. N=6 mice, *P<0.05, bar=50 μm. (C) GFP/PCM1/WGA triple-staining in adult hearts 28 days after injection of AAV9-shcircNfix or PBS. N=6 mice, *P<0.05, bar=50 μm. (D) ISH results for circNfix expression of P56 mouse heart at 14 days after injection with AAV9-shcircNfix or AAV9-shNC virus. *P<0.05 vs shNC, $^{\#}P$<0.05 vs. negative control. N=6 mice, *P<0.05, bar=50 μm. (E) QRT-PCR analysis of circNfix expression in P56 mouse hearts at 0, 14 and 28 days after transfection with AAV9-shcircNfix and AAV9-shNC. The *y* axis shows circNfix expression in AAV9-shcircNfix group versus that in AAV9-shNC group. *P<0.05, n=6.

Supplemental Figure 9 The transfection specificity of AAV9-shcircnfix. (A) GFP/α-SMA double-staining in heart sections illustrating the lack of GFP expression within coronary vessels. The immunostaining was performed in myocardium tissue isolated from adult mouse hearts on day 28 after injection of AAV9-shcircNfix. The coronary vessel was indicated by arrow, bar=50 μm. (B) GFP/cTnT double-staining in cardiomyocytes isolated from adult mouse hearts on day 28 after injection of AAV9-shcircNfix or PBS, bar=50 μm. (C) GFP/Thy1+ double-staining in fibroblasts isolated from adult mouse hearts on day 28 after injection of AAV9-shcircNfix or PBS, bar=50 μm. (D) Quantitative analysis of GFP+ cardiomyocytes (cTnT+) and cardiac fibroblasts (Thy1+) isolated from adult mouse hearts on day 28 after injection of AAV9-shcircNfix or PBS. 375 cardiomyocytes and 326 fibroblasts from 6 mice in AAV9-shcircNfix group, 378 cardiomyocytes and 341 fibroblasts from 6 mice in PBS group. (E) Western blotting for detecting GFP expression in cardiomyocytes and non-cardiomyocytes isolated from adult mouse hearts on day 28 after injection of AAV9-shcircNfix. (F) QRT-PCR for detecting circNfix expression in cardiomyocytes and non-cardiomyocytes isolated from adult mouse hearts on day 28 after injection of AAV9-shcircNfix, n=6, $^*P < 0.05$.

Supplemental Figure 10 Aurora B immunofluorescence staining in adult mouse hearts after circNfix knockdown. (A) Immunostaining of Aurora B in adult mouse hearts at 14 days after injection with AAV9-shcircNfix. The images were captured by

Leica (TCS Sp8) confocal microscope. Aurora B-positive CMs were indicated by arrows. 2642 CMs from 6 mice in shcircNfix group, 2810 CMs from 6 mice in shNC group, *P<0.05, bar=50μm. (B) Immunostaining of Aurora B in at 14 days after injection with AAV9-shcircNfix group 14 days after myocardial infarction (MI). The images were captured by Leica (TCS Sp8) confocal microscope. Aurora B-positive CMs were indicated by arrows. 2381 CMs from 6 mice in shcircNfix group, 2691 CMs from 6 mice in shNC group, *P<0.05, bar=50μm.

Supplemental Figure 11 CircNfix knockdown promotes de-differentiation in adult mouse hearts. (A) QRT-PCR assays detecting the expression of de-differentiation markers, including RUNX1, DAB2, α-SMA and Nkx2.5, n=6, *P<0.05. (B) Western blot assay detecting RUNX1 expression in adult hearts, n=6, *P<0.05. (C) CM sarcomeric status evaluation *In vivo* by immunofluorescence analysis of cTnT in adult mouse heart at 2 weeks and 8 weeks after transfection with AAV9-shcircNfix or AAV9-shNC. Bar=50μm. (D) Histological analysis of adult mouse heart using haematoxylin–eosin (HE) staining at 2 weeks and 8 weeks after transfection with AAV9-shcircNfix or AAV9-shNC. Bar=50μm.

Supplemental Figure 12 CircNfix downregulation promotes de-differentiation in adult CMs. (A) Representative images of isolated adult CMs in P56 mouse hearts 14 days after transfection with AAV9-shcircNfix and AAV9-shNC virus. Scale bars, 50μm. Arrow indicates mononucleate CM. For comparison of total CM number and

mononucleate CM proportion, n=10 mice per group, **P* < 0.05. (B) Adult CM dedifferentiation analysis by immunofluorescence staining for RUNX1 (267 CMs and 288 CMs from 6 mice in shcircNfix group for 14 days and 56 days after transfection, 271 CMs and 284 CMs from 6 mice in shNC group for 14 days and 56 days after transfection, respectively). De-differentiated CMs were indicated by arrows, **P*<0.05 vs. shNC group in corresponding time points, Bar=50 μm. (C) Adult CM dedifferentiation analysis by immunofluorescence staining for α-SMA (261 CMs and 257 CMs from 6 mice in shcircNfix group for 28 days and 56 days, 262 CMs and 250 CMs from 6 mice in shNC group for 28 days and 56 days, respectively). De-differentiated CMs were indicated by arrows, **P*<0.05 vs. shNC group in corresponding time points, Bar=50 μm. (D) Adult CM dedifferentiation analysis by immunofluorescence staining for Nkx2.5 (250 CMs and 259 CMs from 6 mice in shcircNfix group for 28 days and 56 days, 257 and 267 CMs from 6 mice in shNC group for 28 days and 56 days, respectively). De-differentiated CMs were indicated by arrows, **P*<0.05 vs. shNC group in corresponding time points, Bar=50 μm. (E) Adult CM dedifferentiation analysis by immunofluorescence staining for DAB2 (243 CMs and 237 CMs from 6 mice in shcircNfix group for 28 days and 56 days, 250 CMS and 228 CMs from 6 mice in shNC group for 28 days and 56 days, respectively). De-differentiated CMs were indicated by arrows, **P*<0.05 vs. shNC group in corresponding time points, Bar=50 μm.

Supplemental Figure 13 EdU staining in P0 cardiac fibroblasts 24h after

transfection with si-circNfix or si-NC. N=400 fibroblasts pooled from the analysis of 5 mice, bar=50μm.

Supplemental Figure 14 Generation of circNfix-deficient HL-1 CMs using Crispr-Cas9 technology. (A) Single guide RNAs (sgRNAs) designed for targeting the mouse circNfix loci. The single-stranded donor oligonucleotides (ssODN, indicated by green bars) is complementary to the strand in the direction of desired modification. Scale bar, 5kb. (B) QRT-PCR assays detecting circNfix expression in circNfix-deficient HL-1 CMs and control HL-1 CMs. *P < 0.05, n=5 for each group. (C) Immunostaining of pH3 in circNfix-deficient HL-1 CMs. In Cri-circNfix+Nfix group, vector expressing linear Nfix were transfected into circNfix-deficient HL-1 CMs. 4431 CMs for Cri-empty group, 4782 CMs for Cri-circNfix group, 4424 CMs for Cri-circNfix+Nfix group, *P < 0.05 vs. Cri-empty, #P < 0.05 vs. Cri-circNfix; bar=50 μm. (D) Immunostaining of pH3 in circNfix-deficient HL-1 CMs. In Cri-circNfix+Nfix group, vector expressing linear Nfix were transfected into circNfix-deficient HL-1 CMs. 4893 CMs for Cri-empty group, 4672 CMs for Cri-circNfix group, 4618 CMs for Cri-circNfix+Nfix group. *P < 0.05 vs. Cri-empty; bar=50 μm.

Supplemental Figure 15 Generation of circNfix-deficient mice using CRISPR-Cas9 technology. (A) Schematic showing experimental procedure for delivery of AAV9 expressing sgRNA and ssODN into the myocardium tissue of Cas9

knockin mice. (B) Immunostaining of Cas9 in Cas9 transgenic mouse hearts. Cas9+: Cas9 transgenic mice; WT: wide type C57BL/6 mice, bar=50 μm. (C) Western blotting of Cas9 protein in Cas9 mouse hearts. Cas9+: Cas9 transgenic mice; WT: wide type C57BL/6 mice. (D) Immunostaining of GFP in Cas9 mouse hearts at 28 days after injection of AAV9-sgRNA(circNfix)-GFP and AAV9-ssODN(circNfix)-mCherry. Bar=50 μm. (E) Immunostaining of GFP in CMs isolated from Cas9 mouse hearts at 28 days after injection of AAV9-sgRNA(circNfix)-GFP and AAV9-ssODN(circNfix)-mCherry. Bar=50 μm. (F) QRT-PCR assays detecting circNfix expression in Cas9 mouse hearts at 28 days after injection of AAV9-sgRNA(circNfix)-GFP and AAV9-ssODN(circNfix)-mCherry. Cas9 mice injected with AAV9-sgNC were used as control group (Cri-empty). $*P < 0.05$, n=5 for each group. (G) ISH assays detecting circNfix expression in Cas9 mouse hearts at 28 days after injection of AAV9-sgRNA(circNfix)-GFP and AAV9-ssODN(circNfix)-mCherry. Cas9 mice injected with AAV9-sgNC were used as control group (Cri-empty). $*P < 0.05$, n=5 for each group, bar=50 μm. (H) Immunostaining of pH3 in Cas9 mouse hearts at 28 days after injection of AAV9-sgRNA(circNfix)-GFP and AAV9-ssODN(circNfix)-mCherry. Cas9 mice injected with AAV9-sgNC were used as control group (Cri-empty). 3841 CMs from 6 mice in Cri-circNfix group, 3576 CMs from 6 mice in Cri-empty group), $*P<0.05$, bar=50 μm. (I) Immunostaining of Ki67 in Cas9 mouse hearts at 28 days after injection of AAV9-sgRNA(circNfix)-GFP and AAV9-ssODN(circNfix)-mCherry. Cas9 mice injected with AAV9-sgNC were used as control group (Cri-empty). 4001

CMs from 6 mice in Cri-circNfix group, 3934 CMs from 6 mice in Cri-empty group, *P<0.05, bar=50 μm. (J) Immunostaining of Aurora B in Cas9 mouse hearts at 28 days after injection of AAV9-sgRNA(circNfix)-GFP and AAV9-ssODN(circNfix)-mCherry. Cas9 mice injected with AAV9-sgNC were used as control group (Cri-empty). 4001 CMs from 6 mice in Cri-circNfix group, 3934 CMs from 6 mice in Cri-empty group, *P<0.05, bar=50 μm. (K) Immunostaining of Ki67 in CMs isolated from Cas9 mouse hearts at 28 days after injection of AAV9-sgRNA(circNfix)-GFP and AAV9-ssODN(circNfix)-mCherry. Cas9 mice injected with AAV9-sgNC were used as control group (Cri-empty). 461 CMs from 6 mice in Cri-circNfix group, 451 CMs from 6 mice in Cri-empty group, *P<0.05, bar=50 μm.

Supplemental Figure 16 EdU staining in AAV9-shcircNfix and AAV9-shNC groups 14 days after MI. EdU+ CMs were indicated by arrows. *P < 0.05 vs. shNC, n=6, bar=50μm.

Supplemental Figure 17 CircNfix downregulation promotes neovascularization following MI. (A-B) Capillaries densities evaluation by immunohistochemical staining of vWF and CD31 in border zone (A) and scar zone (B) of AAV9-shcircNfix and AAV9-shNC transfected heart sections 2 weeks after MI, n = 40 fields pooled form the analysis of 6 mice. *P < 0.05 vs. shNC group, bar=50μm. (C-D) Capillaries densities evaluated by immunofluorescence analysis of CD31 in border zone (C) and

scar zone (D) of AAV9-shcircNfix and AAV9-shNC transfected heart sections 2 weeks after MI, bar=50μm. (E) Immunofluorescence analysis of CD31/Ki67 for detecting endothelial cells proliferation in AAV9-shcircNfix and AAV9-shNC transfected heart sections 2 weeks after MI, bar=50μm. (F) Quantitative analysis of CD31+ vessel density in AAV9-shcircNfix and AAV9-shNC transfected heart sections 2 weeks after MI, n = 20 fields pooled from the analysis of 6 mice. *P < 0.05 vs. AAV9-shNC group. (G) Quantitative analysis of the ratio of Ki67+ endothelial cells AAV9-shcircNfix and AAV9-shNC transfected heart sections 2 weeks after MI, n = 30 fields pooled from the analysis of 6 mice. *P < 0.05.

Supplemental Figure 18 Silencing circNfix increased arteriolar density following MI (A) Arteriolar densities evaluated by immunofluorescence analysis of α-SMA in AAV9-shcircNfix and AAV9-shNC infected heart sections 2 weeks after MI. n = 20 fields pooled from the analysis of 6 mice. *P < 0.05 vs. shNC group, bar=50μm. (B) Arteriolar densities evaluated by immunofluorescence analysis of α-SMA/CD31 in scar zone of AAV9-shcircNfix and AAV9-shNC group on day 14 after MI. n = 20 fields pooled from the analysis of 6 mice. *P < 0.05 vs. shNC group, bar=50μm.

Supplemental Figure 19 The transfection efficiency of ADV-circNfix. (A) *Ex vivo* bioluminescence images on day 7 after injection with eGFP-labelled ADV-circNfix. (B) GFP/cTnT double-staining in P0 hearts on day 7 after injection of ADV-circNfix, bar=50 μm. (C) ISH results for circNfix expression of P0 mouse hearts on day 7 after

injection with ADV-circNfix. *P<0.05 vs. vector, #P<0.05 vs. negative control (NC), n=6, Bar=50μm. (D) QRT-PCR analysis of circNfix expression of P0 mouse hearts on day 0, 3, 7 after transfection with ADV-circNfix and ADV-vector. The *y* axis shows circNfix expression in ADV-circNfix group versus that in ADV-vector group. *P<0.05, n=6.

Supplemental Figure 20 The transfection specificity of ADV-circNfix. (A) Representative image illustrating the lack of GFP expression within coronary vessels. The immunostaining was performed in P0 hearts on day 7 after injection of ADV-circNfix, bar=50 μm. The white arrow refers the coronary vessels. (B) GFP/cTnT double-staining in cardiomyocytes isolated from P0 hearts on day 7 after injection of ADV-circNfix, bar=50 μm. (C) GFP/CD31 double-staining in endothelial cells isolated from P0 hearts on day 7 after injection of ADV-circNfix, bar=50 μm. (D) GFP/Thy1 double-staining in fibroblasts isolated from P0 hearts on day 7 after injection of ADV-circNfix, bar=50 μm. (E) Quantitative analysis of GFP+ cardiomyocytes, endothelial cells and fibroblasts isolated from P0 hearts on day 7 after injection of ADV-circNfix. 446 cardiomyocytes, 397 endothelial cells and 467 fibroblasts from 20 mice in ADV-circNfix group, 428 cardiomyocytes, 387 endothelial cells and 429 fibroblasts from 20 mice in PBS groups. *P<0.05 vs. PBS group for each cell type. (F) Western blotting for detecting GFP expression in cardiomyocytes, fibroblasts and endothelial cells isolated from P0 hearts on day 7 after injection of ADV-circNfix. (G) QRT-PCR for detecting circNfix expression in

cardiomyocytes, endothelial cells and fibroblasts isolated from P0 hearts on day 7 after injection of ADV-circNfix and ADV-vector. *P<0.05, n=6 for each group. (H) QRT-PCR for detecting circNfix expression in human coronary artery endothelial cells (HCAEC) and mouse cardiac fibroblasts (MCF) on day 4 after transfection of ADV-circNfix *in vitro*. *P<0.05, n=5 for each group. (I) Cell viability of HCAEC and MCF on day 4 after transfection of ADV-circNfix *in vitro*. *P<0.05, n=5 for each group.

Supplemental Figure 21 Immunofluorescence analysis of CD31 in border zone of ADV-circNfix and ADV-vector group on day 7 after MI. N = 25 fields pooled from the analysis of 6 mice. *P < 0.05, bar=50μm.

Supplemental Figure 22 CircNfix had no significant effect on CM hypotrophy. (A) CM size evaluation by WGA staining in in adult mouse hearts 28 days after transfection with AAV9-shcircNfix and AAV9-shNC. (148 CMs from 5 mice in shcircNfix group, 142 CMs from 5 mice in shNC group). bar=50μm. (B) CM size evaluation by WGA staining in in P0 neonatal mouse hearts 7 days after transfection with ADV-circNfix and ADV-vector. (124 CMs from 5 mice in OE group, 131 CMs from 5 mice in vector group), bar=50μm. (C) Adult CMs volume evaluation by cTnT immunofluorescence. The adult CMs were isolated from adult mouse hearts 28 days after transfection with AAV9-shcircNfix and AAV9-shNC (n = 4 for both groups, total of 200 cardiomyocytes analyzed), bar=50μm. (D) Neonatal CMs volume evaluation

by cTnT immunofluorescence. The neonatal CMs were isolated from P0 neonatal mouse hearts 7 days after transfection with ADV-circNfix and ADV-vector (n = 4 for both groups, total of 240 cardiomyocytes analyzed), bar=50μm.

Supplemental Figure. 23 H3K27ac signature of circNfix-SE locus in different tissues. (A) H3K27ac signature of the locus encompassing circNfix-SE in different human tissues. The red bar highlights circNFIX-SE region. (B) H3K27ac signature of the locus encompassing circNfix-SE in different mouse tissues. The red bar highlights circNfix-SE region. (C) Diagrams of mouse Nfix genomic regions with its SE, which was related to Fig. 6C. The stick bars depict position of primers (named A-J) used for detection of chromatin looping and the green arrows indicate Mbo1 enzyme digestion sites. Constant primer at the anchor point is also indicated. TSS indicates the Nfix gene transcription starting site.

Supplemental Figure 24 Generation of Meis1 deficient mice using Crispr-Cas9 technology. (A) SgRNA designed for targeting the mouse Meis1 loci and representative sequencing results from Cas9 mouse hearts injected with AAV9-sgRNA(Meis1). Scale bar, 1kb. (B) Immunostaining of GFP in Cas9 mouse hearts at 28 days after injection with AAV9-sgRNA(Meis1)-mCherry or AAV9-circNfix-GFP, bar=50 μm. (C) Immunostaining of GFP in CMs isolated from Cas9 mouse hearts at 28 days after injection with AAV9-sgRNA(Meis1)-mCherry or AAV9-circNfix-GFP, bar=50 μm. (D) Western blotting of Cas9, Meis1 and pH3 in

Cas9 mouse hearts at 28 days after injection with AAV9-sgNC, AAV9-sgRNA(Meis1), AAV9-circNfix or AAV9-shcircNfix. *P<0.05 vs. SgNC, #P<0.05 vs. sgMeis1+circNfix, 6 mice for each group, bar=50 μm.

Supplemental Figure 25 Detection of CM proliferation in Meis1-deficient mice. (A-B) Detection of circNfix expression by ISH assays (A) in Cas9 mouse hearts at 28 days after injection of AAV9-sgNC, AAV9-sgRNA(Meis1), AAV9-circNfix or AAV9-shcircNfix, and the corresponding quantitative analysis (B). WT: wide type C57BL/6 mice. *P<0.05 vs. SgNC, #P<0.05 vs. sgMeis1+circNfix, n=5 for each group. (C) Detection of circNfix expression by qPCR assays for Cas9 mouse hearts at 28 days after injection of AAV9-sgNC, AAV9-sgRNA(Meis1), AAV9-circNfix or AAV9-shcircNfix; *P<0.05 vs. SgNC, #P<0.05 vs. sgMeis1+circNfix, n=5 for each group. (D) Immunostaining of Aurora B in Cas9 mouse hearts at 28 days after injection of AAV9-sgNC, AAV9-sgRNA(Meis1), AAV9-circNfix or AAV9-shcircNfix; *P<0.05 vs. SgNC, #P<0.05 vs. sgMeis1+circNfix (3697 CMs from 6 mice in WT group, 3252 CMs from 6 mice in SgNC group, 3905 CMs from 6 mice in SgMeis1 group, 3655 CMs from 6 mice in SgMeis1+AAV9-circNfix, 3517 CMs from 6 mice in SgMeis1+AAV9-shcircNfix), *P<0.05 vs. SgNC, #P<0.05 vs. sgMeis1+circNfix, white arrows refer aurora B-positive CMs, bar=50 μm. (E) Immunostaining of pH3 in Cas9 mouse hearts at 28 days after injection with AAV9-sgRNA-Meis1 or AAV9-circNfix (3111 CMs from 6 mice in WT group, 3383 CMs from 6 mice in sgNC group, 3919 CMs from 6 mice in sgMeis1 group, 3955 CMs from 6 mice in

sgMeis1+circNfix, 3790 CMs from 6 mice in SgMeis1+AAV9-shcircNfix), **P*<0.05 vs. sgNC, #*P*<0.05 vs. sgMeis1+circNfix, white arrows refer pH3-positive CMs, bar=50 µm. (F) Immunostaining of Ki67 in Cas9 mouse hearts at 28 days after injection with AAV9-sgRNA-Meis1 or AAV9-circNfix (3265 CMs from 6 mice in wide type group, 3057 CMs from 6 mice in sgNC group, 3469 CMs from 6 mice in sgMeis1 group, 3625 CMs from 6 mice in sgMeis1+AAV9-circNfix, 3893 CMs from 6 mice in SgMeis1+AAV9-shcircNfix), **P*<0.05 vs. SgNC, #*P*<0.05 vs. sgMeis1+circNfix, white arrows refer Ki67-positive CMs, bar=50 µm. (G) Immunostaining of Ki67 in CMs isolated from Cas9 mouse hearts at 28 days after injection with AAV9-sgRNA-Meis1 or AAV9-circNfix (420 CMs from 6 mice in wide type group, 485 CMs from 6 mice in sgNC group, 461 CMs from 6 mice in sgMeis1 group, 434 CMs from 6 mice in SgMeis1+AAV9-circNfix, 501 CMs from 6 mice in sgMeis1+AAV9-shcircNfix), **P*<0.05 vs. SgNC, #*P*<0.05 vs. sgMeis1+circNfix, bar=50 µm.

Supplemental Figure 26 The effect of Meis1 overexpression and circNfix knockdown on CM proliferation. (A) Immunostaining of Ki67 in P0 CMs after circNfix and Meis1 interference. White arrows refer Ki67-positive CMs. 406 CMs from 10 mice in ADV-vector group, 437 CMs from 10 mice in ADV-Meis1 group, 494 CMs from 10 mice in ADV-shcircNfix+ADV-Meis1. **P* < 0.05 vs. ADV-Meis1; bar=50 µm. (B) Immunostaining of pH3 in P0 CMs after circNfix and Meis1 interference. White arrows refer pH3-positive CMs. 503 CMs from 10 mice in

ADV-vector group, 414 CMs from 10 mice in ADV-Meis1 group, 457 CMs from 10 mice in ADV-shcircNfix+ADV-Meis1. **P* < 0.05 vs. ADV-Meis1; bar=50 μm. (C) Immunostaining of Aurora B in P0 CMs after circNfix and Meis1 interference. White arrows refer Aurora B-positive CMs. 413 CMs from 10 mice in ADV-vector group, 376 CMs from 10 mice in ADV-Meis1 group, 395 CMs from 10 mice in ADV-shcircNfix+ADV-Meis1. **P* < 0.05 vs. ADV-Meis1; bar=50 μm. (D) Immunostaining of Ki67 in P0 mouse hearts 4 days after circNfix and Meis1 interference (2538 CMs from 6 mice in ADV-vector group, 2794 CMs from 6 mice in ADV-Meis1 group, 2819 CMs from 6 mice in ADV-shcircNfix+ADV-Meis1). White arrows refer Ki67-positive CMs. **P* < 0.05 vs. ADV-Meis1; bar=50 μm. (E) Immunostaining of pH3 in P0 mouse hearts 4 days after circNfix and Meis1 interference (2160 CMs from 6 mice in ADV-vector group, 2134 CMs from 6 mice in ADV-Meis1 group, 2521 CMs from 6 mice in ADV-shcircNfix+ADV-Meis1). White arrows refer pH3-positive CMs. **P* < 0.05 vs. ADV-Meis1; bar=50 μm. (F) Immunostaining of Aurora B in P0 mouse hearts 4 days after circNfix and Meis1 interference (2631 CMs from 6 mice in ADV-vector group, 2148 CMs from 6 mice in ADV-Meis1 group, 2785 CMs from 6 mice in ADV-shcircNfix+ADV-Meis1). White arrows refer pH3-positive CMs. **P* < 0.05 vs. ADV-Meis1; bar=50 μm.

Supplemental Figure 27 The synergistic effect of Meis1 and circNfix double knockdown on CM proliferation. (A) Immunostaining of Ki67 in P7 CMs after circNfix and Meis1 interference. White arrows refer Ki67-positive CMs. 475 CMs

from 10 mice in si-NC group, 502 CMs from 10 mice in si-circNfix group, 469 CMs from 10 mice in si-Meis1 group, 437 CMs from 10 mice in si-circNfix+si-Meis1. *P < 0.05 vs. si-NC, bar=50 μm. (B) Immunostaining of EdU in P7 CMs after circNfix and Meis1 interference. White arrows refer EdU-positive CMs. (462 CMs from 10 mice in si-NC group, 521 CMs from 10 mice in si-circNfix group, 490 CMs from 10 mice in si-Meis1 group, 541 CMs from 10 mice in si-circNfix+si-Meis1). *P < 0.05 vs. si-NC, bar=50 μm. (C) Immunostaining of pH3 in P7 CMs after circNfix and Meis1 interference. White arrows refer pH3-positive CMs. 559 CMs from 10 mice in si-NC group, 533 CMs from 10 mice in si-circNfix group, 516 CMs from 10 mice in si-Meis1 group, 545 CMs from 10 mice in si-circNfix+si-Meis1. *P < 0.05 vs. si-NC, bar=50 μm. (D) Immunostaining of Aurora B in P7 CMs after circNfix and Meis1 interference. White arrows refer Aurora B-positive CMs. 402 CMs from 10 mice in si-NC group, 447 CMs from 10 mice in si-circNfix group, 431 CMs from 10 mice in si-Meis1 group, 480 CMs from 10 mice in si-circNfix+si-Meis1. *P < 0.05 vs. si-NC, bar=50 μm.

Supplemental Figure 28 The association between circNfix and Ybx1. (A) Structures and probe sequence of circNfix. (B) The predicted interaction between Ybx1 and circNfix, Nedd4l and circNfix using RNA-Protein interaction Prediction (RPISeq) programme. Predictions with probabilities > 0.5 were considered "positive," i.e., indicating that the corresponding RNA and protein are likely to interact. (C) Several Ybx1 motifs was observed in circNfix. Upper, the Ybx1 motif; Bottom, the

Ybx1 motifs in mmu-circNfix sequence. (D) EdU staing in P7 CMs after Ybx1 overexpression. White arrows refer EdU-positive CMs. 384 CMs from 7 mice in OE-Ybx1 group, 365 CMs from 7 mice in vector group, *P<0.05, bar=50 μm. (E) Western blotting analysis and quantitative analyses of Ybx1 protein levels in adult mouse heart at 4 weeks after transfection with AAV9-shcircNfix or AAV9-shNC. *P<0.05, n=4. (F) 3D viewing of the interaction between Ybx1 and Nedd4l using Protein docking program (Hex Service). Free energy (Delta G) for protein binding and Ionization constant (Kd) was calculated using PPA-Pred program. (G) Western blotting analysis and quantitative analyses of Nedd4l protein levels in CMs after circNfix or Ybx1 interference. *P<0.05 vs si-NC, n=5.

Supplemental Figure 29 The effect of circNfix, Ybx1, miR-214 and Gsk3β interference on CM proliferation. (A-B) Immunostaining of EdU (A) and Ki67 (B) in P7 CMs after circNfix, Ybx1, miR-214 and Gsk3β interference. White arrows refer EdU-positive or Ki67-positive CMs. bar=50 μm. (C) Statistical analysis of the ratio of pH3+, Aurora B+, Edu+ and Ki67+ CMs after circNfix, Ybx1, miR-214 and Gsk3β interference. *P < 0.05 vs. siNC, #P < 0.05 vs. si-circNfix+si-Ybx1+anta-214; 400 CMs from 10 mice for each group.

Supplemental Figure 30 The association between circNfix, miR-214 and Gsk3β. (A) Illustration of the putative binding sites of miR-214/761 associated with circNfix. The mutated sequence was related to LUC-circNfix-mut that was used in Fig. 8C. (B)

Expression of target genes after miR-214 expression according to qRT-PCR. These target genes for miR-214 were predicted by TargetScan programme. *P<0.05 vs. mimic-NC group, n=4. (C) The conservation of the putative binding sites of miR-214 associated with Gsk3β. The mutated sequence was related to LUC-circNfix-mut that was used in Fig. 8G. (D) The effect of miR-214 mimics on Ybx1 expression. *P<0.05, n=4. (E) The effect of Ybx1 overexpression on miR-214 expression. *P<0.05, n=4. (F) EdU staining in P7 CMs after miR-214 overexpression. White arrows refer EdU-positive CMs, 295 CMs from 6 mice in miR-214-mimic group, 274 CMs from 6 mice in mimic-NC group, EdU-positive CMs were indicated by arrows, *P<0.05, bar=50 μm.

Supplemental Figure 31 The effect of circNfix and Gsk3β interference on angiogenesis. (A) ELISA assays showing the effect of circNfix, miR-214 and Gsk3β interference on CMs secreting Vegf. *P<0.05 vs. control group, $^{\#}P$<0.05 vs. si-circNfix group, n=4. (B) Immunostaining of GFP in adult mouse hearts at 28 days after injection of PBS, AAV9-shcircNfix-GFP, AAV9-Gsk3β-mCherry and AAV9-shcircNfix-GFP+AAV9-Gsk3β-mCherry, bar=50 μm. (C) Western blotting of GFP in adult mouse hearts at 28 days after injection of AAV9-shNC, AAV9-shcircNfix-GFP, AAV9-Gsk3β-mCherry and AAV9-shcircNfix-GFP+AAV9-Gsk3β-mCherry. *P < 0.05 vs. shNC; #P < 0.05 vs. shcircNfix; 5 mice for each group, bar=50 μm. (D) IB4 immunostaining in adult mouse hearts in AAV9-shNC, AAV9-shcircNfix and AAV9-shcircNfix+AAV9-Gsk3β

group on day 28 after MI. *P < 0.05 vs. shNC; 6 mice for each group, bar=50 μm. (E) IB4 immunostaining in P0 mouse hearts in ADV-vector, ADV-circNfix and ADV-circNfix+ SB216763 group on day 7 after MI. SB216763 was intraperitoneally injected for one week at a dose of 10 mg/kg/d. *P < 0.05 vs. ADV-vector; 6 mice for each group, bar=50 μm.

Supplemental References:

1. Bajpai G, Bredemeyer A, Li W, Zaitsev K, Koenig AL, Lokshina I, Mohan J, Ivey B, Hsiao H, Weinheimer C. Tissue resident CCR2− and CCR2+ cardiac macrophages differentially orchestrate monocyte recruitment and fate specification following myocardial injury. CIRC RES. 2019;124;263-278.
2. Guojun C, Hairui L, Xinzhong L, Bing L, Lintao Z, Senlin H, Hao Z, Mengsha L, Guoqing J, Wangjun L. Loss of long non-coding RNA CRRL promotes cardiomyocyte regeneration and improves cardiac repair by functioning as a competing endogenous RNA. Journal of Molecular and Cellular Cardiology. 2018;122;152-164.
3. Li X, He X, Wang H, Li M, Huang S, Chen G, Jing Y, Wang S, Chen Y, Liao W. Loss of AZIN2 splice variant facilitates endogenous cardiac regeneration. CARDIOVASC RES. 2018;114;1642-1655.
4. Li B, Hu Y, Li X, Jin G, Chen X, Chen G, Chen Y, Huang S, Liao W, Liao Y. Sirt1 Antisense Long Noncoding RNA Promotes Cardiomyocyte Proliferation by Enhancing the Stability of Sirt1. J AM HEART ASSOC. 2018;7;e9700.
5. Chen Y, Li X, Li B, Wang H, Li M, Huang S, Sun Y, Chen G, Si X, Huang C. Long Non-coding RNA ECRAR Triggers Post-natal Myocardial Regeneration by Activating ERK1/2 Signaling. MOL THER. 2019;27;29-45.
6. Chen Z, Xie J, Hao H, Lin H, Wang L, Zhang Y, Chen L, Cao S, Huang X, Liao W, Bin J, Liao Y. Ablation of periostin inhibits post-infarction myocardial regeneration in neonatal mice mediated by the phosphatidylinositol 3 kinase/glycogen synthase kinase 3β/cyclin D1 signalling pathway. CARDIOVASC RES. 2017;113;620-632.
7. Yukawa K, Tanaka T, Kishino M, Yoshida K, Takeuchi N, Ito T, Takamatsu H, Kikutani H, Kumanogoh A. Deletion of Sema4D gene reduces intimal neovascularization and plaque growth in apolipoprotein E-deficient mice. INT J MOL MED. 2010;26;39-44.
8. Korostowski L, Sedlak N, Engel N. The Kcnq1ot1 Long Non-Coding RNA Affects Chromatin Conformation and Expression of Kcnq1, but Does Not Regulate Its Imprinting in the Developing Heart. PLOS GENET. 2012;8;e1002956.
9. Zheng Q, Bao C, Guo W, Li S, Chen J, Chen B, Luo Y, Lyu D, Li Y, Shi G. Circular RNA profiling reveals an abundant circHIPK3 that regulates cell growth by sponging multiple miRNAs. NAT COMMUN. 2016;7;112-125.
10. Li H, Lu Y, Sun Y, Chen G, Wang J, Wang S, Huang C, Zhong L, Si X, Liao W. Diagnostic Ultrasound and Microbubbles Treatment Improves Outcomes of Coronary No-Reflow in Canine Models by Sonothrombolysis. CRIT CARE MED. 2018;46;e912-e920.
11. Huang C, Huang S, Li H, Li X, Li B, Zhong L, Wang J, Zou M, He X, Zheng H. The effects of ultrasound exposure on P-glycoprotein-mediated multidrug resistance in vitro and in vivo. J EXP CLIN CANC RES. 2018;37;232.
12. Alkass, Kanar, Panula, Joni, Westman, Mattias, Wu, TingDi, GuerquinKern, JeanLuc. No Evidence for Cardiomyocyte Number Expansion in Preadolescent Mice. CELL. 2015;163;1026-1036.
13. Tao Z, Chen B, Tan X, Zhao Y, Wang L, Zhu T, Cao K, Yang Z, Kan YW, Su H. Coexpression of VEGF and angiopoietin-1 promotes angiogenesis and cardiomyocyte proliferation reduces

apoptosis in porcine myocardial infarction (MI) heart. P NATL ACAD SCI USA. 2011;108;2064-2069.
14. Werfel S, Nothjunge S, Schwarzmayr T, Strom TM, Meitinger T, Engelhardt S. Characterization of circular RNAs in human, mouse and rat hearts. Journal of Molecular & Cellular Cardiology. 2016;98;103-107.
15. Hnisz D, Abraham B, Lee TI, Lau A, Saint-André V, Sigova A, Hoke H, Young R. Super-Enhancers in the Control of Cell Identity and Disease. CELL. 2013;155;934-947.
16. Khan A, Zhang X. dbSUPER: a database of super-enhancers in mouse and human genome. NUCLEIC ACIDS RES. 2016;44;D164-D171.

Supplemental Movie. 1: Time-lapse imaging of P7 CMs transfected with si-circNfix, corresponding to Fig. 2E. Time-lapse imaging started 12hr post-transfection (1/3 hr/Frame).

Supplemental Movie. 2: Time-lapse imaging of P7 CMs transfected with si-NC, corresponding to Fig. 2E. Time-lapse imaging started 12hr post-transfection (1/3 hr/Frame).
